# Supplementary material for: First-Order Symmetry-Adapted Perturbation Theory with Double Exchange for Multireference Systems
Source: J Chem Theory Comput. 2025 Aug 20;21(17):8362–74. doi: 10.1021/acs.jctc.5c00629 (PMC12424174; doi:10.1021/acs.jctc.5c00629)
Supplement: Supplementary file 1 [file ct5c00629_si_001.pdf]

Supporting Information: First-Order Symmetry-Adapted Perturbation Theory with Double-Exchange for Multireference Systems

Dominik Cieśliński,* Michał Przybytek, Grzegorz Chałasiński, and Michał Hapka

University of Warsaw, Faculty of Chemistry, ul. L. Pasteura 1, 02-093 Warsaw, Poland

E-mail: d.cieslinski@uw.edu.pl

Contents

SAPT(FCI) implementation for two-electron monomers **S-3**

Be$\cdots$Be frozen-core approximation S-5

Second-quantized derivation of first-order exchange energy in SAPT(APSG) **S-8**

1 Diagramatic representation of operators **S-10**

2 Diagrammatic representation of arbitrary geminals **S-17**

2.1 Example S-30

3 Simplifications for APSG wave function **S-30**

3.1 Example S-35

4 Diagrammatic representation in tensor product of Fock-spaces **S-36**

5 Diagrammatic representation of SAPT operators **S-38**

6 Rules for evaluating first-order SAPT exchange diagrams **S-43**

7 Examples **S-45**

7.1 Example 1: $\langle \hat{\mathcal{P}}_2 \rangle$ S-45

7.2 Example 2: An example diagram in $\langle \hat{V}_{\text{ee}} \hat{\mathcal{P}}_2 \rangle$ S-46

7.3 Example 3: $\langle \hat{V}_A \hat{\mathcal{P}}_2 \rangle$ S-47

7.4 Examples of equivalency in diagrams S-48

References **S-50**

Working expressions and diagrams **S-51**

SAPT(FCI) implementation for two-electron monomers

Since the nonrelativistic Born-Oppenheimer Hamiltonian for an arbitrary molecular system does not explicitly depend on spin variables, it is possible to perform SAPT(FCI) calculations using the spin-free approach. The spatial wave function of a ground state closed-shell two-electron monomer forms a basis of the one-dimensional irreducible representation (irrep) [2] of the S_2 symmetric group. Thus, we can assume that the monomer Hamiltonians $\hat{H}_A$ and $\hat{H}_B$ are defined in the Hilbert spaces $\mathcal{H}_A$ and $\mathcal{H}_B$, respectively, of spin-independent functions the symmetry of which is characterized by the Young diagram:

$$\yng(2). \tag{0.1}$$

The unperturbed Hamiltonian, $\hat{H}_0 = \hat{H}_A + \hat{H}_B$, is acting in the Hilbert space $\mathcal{H}_A \otimes \mathcal{H}_B$ spanned by all product functions $f^A(\mathbf{r}_1, \mathbf{r}_2) f^B(\mathbf{r}_3, \mathbf{r}_4)$, where $f^A(\mathbf{r}_1, \mathbf{r}_2) \in \mathcal{H}_A$ and $f^B(\mathbf{r}_3, \mathbf{r}_4) \in \mathcal{H}_B$. However, the full Hamiltonian $\hat{H}$ of the interacting system is invariant with respect to all possible permutations of the $\mathbf{r}_i$, $i = 1, \ldots, 4$, variables. Therefore, its eigenfunctions and eigenvalues belong to various irreps $[\lambda]$ of the S_4 group. This allows for a decomposition of $\mathcal{H}_A \otimes \mathcal{H}_B$ into a direct sum of subspaces characterized by well-defined irrep labels,

$$\mathcal{H}_A \otimes \mathcal{H}_B = \mathcal{H}^{[4]} \oplus \mathcal{H}^{[31]} \oplus \mathcal{H}^{[2^2]}, \tag{0.2}$$

which can be deduced from the induced product decomposition:

$$\left(\yng(2) \otimes \yng(2)\right) \uparrow S_4 = \yng(4) \oplus \yng(3,1) \oplus \yng(2,2). \tag{0.3}$$

Only functions of the $[2^2]$ symmetry can form the spatial part of complete antisymmetric wave functions for electrons (spin-$\frac{1}{2}$ fermions). It is possible to access the $\mathcal{H}^{[2^2]}$ subspace by acting on all functions from $\mathcal{H}_A \otimes \mathcal{H}_B$ space with the appropriate projection operator of the

S_4 group,[S1]

$$\hat{Q}^{[2^2]} = \frac{1}{6}(2 + \hat{\mathcal{P}}_2 + 2\hat{\mathcal{P}}_4), \tag{0.4}$$

where $\hat{\mathcal{P}}_2$ and $\hat{\mathcal{P}}_4$ are single and double exchange operators, respectively:

$$\begin{aligned} \hat{\mathcal{P}}_2 &= -(13) - (14) - (23) - (24), \\ \hat{\mathcal{P}}_4 &= (13)(24). \end{aligned} \tag{0.5}$$

The spatial FCI wave function of monomer A can be written as

$$\Psi_A(\mathbf{r}_1, \mathbf{r}_2) = \sum_{1 \le i \le j \le M} c^A_{ij}\, \psi^A_{ij}(\mathbf{r}_1, \mathbf{r}_2), \tag{0.6}$$

where two-electron basis functions $\psi^A_{ij}(\mathbf{r}_1, \mathbf{r}_2)$ adapted to the irrep [2] of S_2 are constructed using a set of M Hartree-Fock orbitals $\phi^A_i(\mathbf{r})$:

$$\psi^A_{ij}(\mathbf{r}_1, \mathbf{r}_2) = \frac{1}{\sqrt{2(1+\delta_{ij})}} \left(\phi^A_i(\mathbf{r}_1)\phi^A_j(\mathbf{r}_2) + \phi^A_j(\mathbf{r}_1)\phi^A_i(\mathbf{r}_2) \right). \tag{0.7}$$

The c^A_{ij} coefficients in Eq. (0.6) are obtained by diagonalization of the Hamiltonian,

$$\hat{H}_A = V_A + h^A(\mathbf{r}_1) + h^A(\mathbf{r}_2) + r_{12}^{-1}, \tag{0.8}$$

where V_A is the nuclear repulsion term, $h^A(\mathbf{r})$ is the standard one-electron Hamiltonian that consists of the kinetic energy operator $t^A(\mathbf{r})$ of an electron and the electron-nuclei attraction potential $v^A(\mathbf{r})$, and $r_{12} = |\mathbf{r}_1 - \mathbf{r}_2|$ is the interelectron distance. The definitions for monomer B are analogous and can be obtained by replacing $A \to B$ and $\mathbf{r}_1, \mathbf{r}_2 \to \mathbf{r}_3, \mathbf{r}_4$.

The first-order interaction energy can be now calculated from the formula

$$E^{(1)}_{\text{int}} = \frac{\langle \Psi_A \Psi_B | \hat{V} \hat{Q}^{[2^2]} | \Psi_A \Psi_B \rangle}{\langle \Psi_A \Psi_B | \hat{Q}^{[2^2]} | \Psi_A \Psi_B \rangle}, \tag{0.9}$$

where $\hat{V}$ is the intermolecular interaction operator

$$\hat{V} = V_{AB} + v^A(\mathbf{r}_3) + v^A(\mathbf{r}_4) + v^B(\mathbf{r}_1) + v^B(\mathbf{r}_2) + r_{13}^{-1} + r_{14}^{-1} + r_{23}^{-1} + r_{24}^{-1}, \tag{0.10}$$

with V_{AB} being the intermonomer nuclear repulsion term. Combining Eqs. (0.4) and (0.9), we find the following explicit expressions for the electrostatic energy and leading terms of the exchange energy in the overlap expansion:

$$E^{(1)}_{\text{elst}} = \langle \hat{V} \rangle, \tag{0.11}$$

$$E^{(1)}_{\text{exch}}(\propto S^2) = \frac{1}{2}\langle \hat{V}\hat{\mathcal{P}}_2 \rangle - \frac{1}{2}\langle \hat{V} \rangle \langle \hat{\mathcal{P}}_2 \rangle \tag{0.12}$$

$$E^{(1)}_{\text{exch}}(\propto S^4) = \langle \hat{V}\hat{\mathcal{P}}_4 \rangle - \frac{1}{4}\langle \hat{V}\hat{\mathcal{P}}_2 \rangle \langle \hat{\mathcal{P}}_2 \rangle - \langle \hat{V} \rangle \langle \hat{\mathcal{P}}_4 \rangle + \frac{1}{4}\langle \hat{V} \rangle \langle \hat{\mathcal{P}}_2 \rangle^2, \tag{0.13}$$

where the shorthand notation $\langle \hat{X} \rangle = \langle \Psi_A \Psi_B | \hat{X} | \Psi_A \Psi_B \rangle$ was used. The presence of factors $\frac{1}{2}$ and $\frac{1}{4}$ is a consequence of using the spin-free approach.

Be$\cdots$Be frozen-core approximation

The beryllium atom has four electrons. After freezing the 1s orbital, it becomes a two-electron system that can be treated as described in the previous section. In our implementation of the frozen-core approximation we employ the index-range restriction (IRR) approach by Patkowski and Szalewicz.[S2]

Let us label the core orbital as 1. In consequence, in the wave function expansion of Eq. (0.6), the summation over i and j is restricted to $2 \leq i \leq j \leq M$. The Hamiltonian $\hat{H}^A$ in Eq. (0.8) is modified as follows

$$\begin{aligned} V_A \to V_A^{\text{fc}} &= \langle \phi_1^A | 2h^A + 2J_1^A - K_1^A | \phi_1^A \rangle, \\ h^A(\mathbf{r}) \to h^{A,\text{fc}}(\mathbf{r}) &= h^A(\mathbf{r}) + 2J_1^A(\mathbf{r}) - K_1^A(\mathbf{r}), \end{aligned} \tag{0.14}$$

where the action of the Coulomb $J_1^A(\mathbf{r})$ and exchange $K_1^A(\mathbf{r})$ operators on an arbitrary

function are defined as

$$J_1^A(\mathbf{r})f(\mathbf{r}) = f(\mathbf{r}) \int \frac{|\phi_1^A(\mathbf{r}')|^2}{|\mathbf{r}-\mathbf{r}'|}\mathrm{d}\mathbf{r}',$$
$$K_1^A(\mathbf{r})f(\mathbf{r}) = \phi_1^A(\mathbf{r}) \int \frac{\phi_1^{A*}(\mathbf{r}')f(\mathbf{r}')}{|\mathbf{r}-\mathbf{r}'|}\mathrm{d}\mathbf{r}'. \tag{0.15}$$

The necessary modifications of the interaction operator $\hat{V}$ in Eq. (0.10) include

$$V_{AB} \rightarrow V_{AB}^{\mathrm{fc}} = \frac{16}{R} + \langle \phi_1^B | 2v^A + 2J_1^A | \phi_1^B \rangle + \langle \phi_1^A | 2v^B + 2J_1^B | \phi_1^A \rangle, \tag{0.16}$$

$$v^A(\mathbf{r}) \rightarrow v^{A,\mathrm{fc}}(\mathbf{r}) = v^A(\mathbf{r}) + 2J_1^A(\mathbf{r}), \tag{0.17}$$

$$v^B(\mathbf{r}) \rightarrow v^{B,\mathrm{fc}}(\mathbf{r}) = v^B(\mathbf{r}) + 2J_1^B(\mathbf{r}), \tag{0.18}$$

where R is the Be–Be distance.

Table S1: First-order exchange energy in the S^2 and $\propto S^4$ approximations for the Be$\cdots$Be dimer computed at different levels of SAPT. Δ_{fc} denotes the fc-correction obtained using HF monomer wave functions: $\Delta_{\mathrm{fc}}(S^{2n}) = E^{(1)}_{\mathrm{exch}}(S^{2n})[\mathrm{HF}] - E^{(1)}_{\mathrm{exch}}(S^{2n})[\mathrm{HF,fc}]$. The equilibrium distance is $R_{\mathrm{eq}} = 4.6\ a_0$. fc denotes the frozen-core approximation. CAS(n,m) refers to the active space on the Be atom. The energy unit is milliHartree. The basis set is aug-cc-pVDZ.

	SAPT(HF)		Δ_{fc}	Δ_{fc}	SAPT(CAS[2,4])		SAPT(CAS[2,45])		SAPT(FCI)	
R/R_{eq}	S^2	$\propto S^4$	S^2	$\propto S^4$	S^2	$\propto S^4$	S^2	$\propto S^4$	S^2	$\propto S^4$
0.90	90.63	15.27	27.30	5.77	80.18	19.67	81.64	20.96	83.37	19.31
0.95	75.68	10.71	19.44	3.52	67.11	14.14	68.33	15.22	69.88	14.25
1.00	62.54	7.37	13.78	2.11	55.50	9.95	56.53	10.81	57.89	10.26
1.05	51.19	4.99	9.73	1.26	45.40	6.86	46.27	7.52	47.42	7.22
1.10	41.54	3.32	6.85	0.73	36.76	4.63	37.50	5.14	38.45	4.98
1.15	33.45	2.18	4.80	0.42	29.49	3.08	30.11	3.45	30.89	3.37
1.20	26.74	1.41	3.36	0.24	23.46	2.01	23.98	2.28	24.61	2.24
1.25	21.23	0.90	2.33	0.13	18.51	1.29	18.95	1.49	19.44	1.46
1.30	16.76	0.57	1.62	0.07	14.51	0.82	14.87	0.96	15.25	0.94
1.35	13.15	0.36	1.11	0.04	11.29	0.52	11.60	0.60	11.88	0.60
1.40	10.27	0.22	0.75	0.02	8.74	0.32	8.99	0.38	9.20	0.38
1.45	7.98	0.14	0.51	0.01	6.72	0.20	6.93	0.23	7.09	0.23
1.50	6.18	0.08	0.35	0.01	5.15	0.12	5.32	0.14	5.43	0.14
2.00	0.40	0.00	0.00	0.00	0.29	0.00	0.31	0.00	0.31	0.00

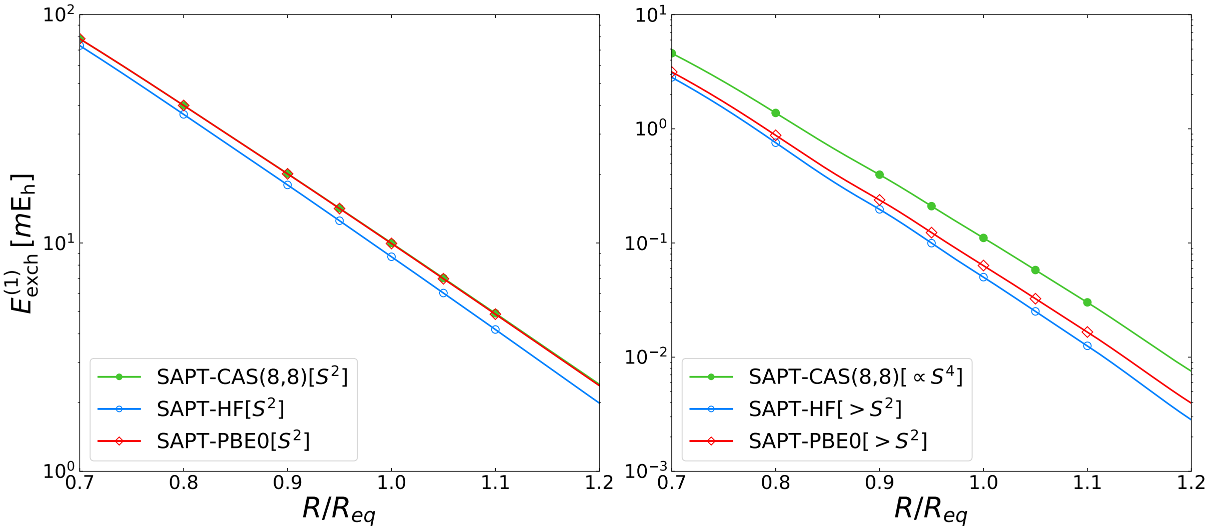

Figure S1: Water dimer: first-order exchange energy terms in the S^2 approximation (left panel) and terms beyond S^2 (right panel) obtained at SAPT(HF), SAPT(CAS), and SAPT(PBE0) levels of theory. SAPT(HF) and SAPT(PBE0) results in the right panel include all terms beyond S^2 to infinite order in overlap, whereas SAPT(CAS) results refer to $\propto S^4$ terms only. Geometries were taken from the S66x10 dataset.[S3] The basis set is aug-cc-pVTZ.

Second-quantized derivation of first-order exchange energy in SAPT(APSG)

We present an overview of the diagrammatic formulation of the first-order exchange energy in SAPT(APSG) beyond the S^2 approximation. This approach is closely related to the second-quantized (SQ) SAPT method proposed by Moszynski et al.[S4] The key difference is that we apply Wick theorem with respect to the physical vacuum rather than the Fermi vacuum, as in Ref. S4. Furthermore, for single-determinant wave functions it is possible to explicitly cancel the disconnected terms in the $E^{(1)}_{\rm exch}(S^2)$ energy expression using the resolution of the identity (RI) on the tensor product of Hilbert spaces of the monomers, $\mathcal{H}_A \otimes \mathcal{H}_B$. This results in the summation over all excited Slater determinants. Since an analogous procedure for the APSG wave function is not straightforward, we avoid employing RI in the derivation. In consequence, the diagrammatic SAPT(APSG) formulation yields energy expressions identical to those obtained from the density-matrix approach presented in the main text. The resulting SQ formulas for the first-order exchange are valid both in the dimer- and monomer-centered basis sets in contrast to the SQ approach of Moszynski et al.[S4] which is restricted to dimer-centered basis sets.

The structure of the overview is as follows. Section 1 introduces the basics of diagrammatic representation of operators and its application to calculating expectation value with respect to the physical vacuum. Section 2 presents a diagrammatic approach to obtaining the expectation value of an arbitrary spin-independent n-body operator with an N-geminal wave function. Section 3 demonstrates how this approach simplifies for the special case of strongly orthogonal geminal wave functions, specifically APSG and GVB-PP. Section 4 extends the formalism to the SQ formulation of SAPT by introducing the diagrammatic representation of operators in the tensor-product space of two Fock spaces corresponding to individual monomers. Section 5 contains some considerations about the diagrammatic representation of the interaction and exchange operator. The final procedure for constructing

SAPT(APSG) diagrams and deriving the corresponding algebraic expressions is presented in Section 6. Section 7 provides example applications of the diagrammatic approach to SAPT(APSG).

Section 1 is based on the Nijmegen lectures of J. Paldus (p. 58-84).[S5] Sections 2 and 3 review the diagrammatic approach introduced by Paldus in Refs. S6 and S7, respectively. Compared to the original formulation, we emphasize the role of the natural orbital representation of geminals and the implications of strong orthogonality for the diagrammatic approach.

1 Diagramatic representation of operators

The goal of this section is to introduce a diagrammatic method of evaluating the expectation value of an arbitrary operator. We begin by recalling the definition of a normal product and Wick theorem. We will use normal product with respect to the physical vacuum.

Consider a product of operators:

$$M_{p_1} M_{p_2} \dots M_{p_k},$$

where each M_{p_i}, $i = 1, \dots, k$, is either a creation $X^\dagger_{p_i}$ or an annihilation X_{p_i} operator. The normal product of these operators, $N[M_{p_1} M_{p_2} \dots M_{p_k}]$, is defined as a product of the same operators reordered so that all creation operators are to the left of all annihilation operators. A phase factor of $(+1)$ or (-1) is assigned depending on whether an even or odd number of transpositions is required to achieve the necessary ordering. For example:

$$N[X^\dagger_p X_q X^\dagger_r] = (-1) X^\dagger_p X^\dagger_r X_q.$$

Additionally, we assume:

$$N[\emptyset] = 1. \tag{1.1}$$

Note that the normal product of one kind of operators does not change that product, that is:

$$N[X^\dagger_{p_1} X^\dagger_{p_2} \dots X^\dagger_{p_k}] = X^\dagger_{p_1} X^\dagger_{p_2} \dots X^\dagger_{p_k},$$

and

$$N[X_{p_1} X_{p_2} \dots X_{p_k}] = X_{p_1} X_{p_2} \dots X_{p_k}.$$

It is also worth noticing that for $k > 0$ the vacuum expectation value of the normal product of operators vanishes:

$$\langle 0 | N[M_{p_1} M_{p_2} \dots M_{p_k}] | 0 \rangle = 0. \tag{1.2}$$

Recall the definition of a contraction of two operators M_{p_1} and M_{p_2} as:

$$\wick{\c M_{p_1} \c M_{p_2}} = M_{p_1} M_{p_2} - N[M_{p_1} M_{p_2}].$$

With the use of the anticommutation relations, it is easy to see that:

$$\wick{\c X_{p_1} \c X_{p_2}} = 0, \quad \wick{\c X^\dagger_{p_1} \c X_{p_2}} = 0, \quad \wick{\c X_{p_1} \c X^\dagger_{p_2}} = \delta_{p_1 p_2}, \quad \wick{\c X^\dagger_{p_1} \c X^\dagger_{p_2}} = 0.$$

Now, we can define the contraction inside the normal product as:

$$\wick{N[\dots \c1 M_{p_1} \dots \c2 M_{p_2} \dots \c3 M_{p_k} \dots \c1 M_{q_1} \dots \c3 M_{q_k} \dots \c2 M_{q_2} \dots]} = (-1)^P \wick{\c M_{p_1} \c M_{q_1}} \wick{\c M_{p_2} \c M_{q_2}} \dots \wick{\c M_{p_k} \c M_{q_k}} N[\dots],$$

where the normal product on the right hand side collects all operators that do not participate in any contraction, all contracted operators are outside of this normal product, and $(-1)^P$ is the sign of the permutation that gives the required ordering of the operators. The next step is to introduce Wick theorem:

Wick Theorem

An arbitrary product of creation and annihilation operators $M_{p_1} M_{p_2} \dots M_{p_k}$ is equal to the sum of the normal product of these operators and all possible normal products with contractions of these operators.

Using this theorem and Eqs. (1.1) and (1.2), we find that $\langle 0| M_{p_1} M_{p_2} \dots M_{p_k} |0\rangle$ is equal to the sum of all possible fully contracted terms. For example:

$$\begin{aligned} \langle 0| X_p X_q X_r^\dagger X_s^\dagger |0\rangle &= \langle 0| \wick{N[\c1 X_p \c2 X_q \c2 X_r^\dagger \c1 X_s^\dagger]} |0\rangle + \langle 0| \wick{N[\c1 X_p \c2 X_q \c1 X_r^\dagger \c2 X_s^\dagger]} |0\rangle \\ &= \wick{\c X_p \c X_s^\dagger} \wick{\c X_q \c X_r^\dagger} - \wick{\c X_p \c X_r^\dagger} \wick{\c X_q \c X_s^\dagger} = \delta_{ps}\delta_{qr} - \delta_{pr}\delta_{qs}. \end{aligned}$$

It is worth mentioning the rule for assigning the correct sign factor in a fully contracted normal product:

The sign factor is equal to $(-1)^Q$, where Q is the number of intersections of the pairing symbols inside the fully contracted normal product.

For example:

$$N[X_p X_q X_r X_q^\dagger X_p^\dagger X_r^\dagger] = (-1)^4 N[\emptyset] = 1. \tag{1.3}$$

This rule will be useful in the diagrammatic representation of Wick theorem. Finally, recall Generalized Wick theorem:

Generalized Wick theorem

An arbitrary product of creation operators, annihilation operators, and normal products of arbitrary products of creation and anihilation operators is equal to the normal product of all these operators plus the sum of normal products with all possible contraction schemes except of these which contain contractions between the operators originating from the same normal product.

The next step is to translate contractions into a diagrammatic representation. We achieve this by representing creation and annihilation operators as solid oriented lines (lines with an arrow on top), referred to as "fermion lines". To track their origin, we attach them to specific vertices, depicted as dots, or sets of vertices interconnected by nonoriented vertical lines; such objects will be referred to as supervertices (s-vertices). A solid line with index p leaving a vertex represents a creation operator $X_p^\dagger$, while a line labeled p entering a vertex represents an annihilation operator X_p. A vertex or s-vertex with attached fermion lines represents a basic operator. If each vertex has one incoming and one outgoing line, the operator is said to preserve the total number of electrons in the system. As an example, consider representation of a typical two-body operator:

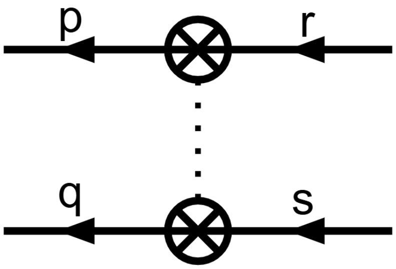

With this diagram we associate a scalar $d = \langle pq|rs\rangle = (pr|qs)$ and an operator $\hat{D}_V$, which is the product of the scalar d and the operator $X_p^\dagger X_q^\dagger X_s X_r = N[X_p^\dagger X_q^\dagger X_s X_r]$, i.e. $\hat{D}_V(p,q,r,s) = (pr|qs)X_p^\dagger X_q^\dagger X_s X_r$. Reading the operator part from an s-vertex has a particular order: we read operators from the left to the right; we start with writing down creation operators, which we do going from the top to the bottom, and then we write down annihilation operators, which we do going from the bottom to the top.

When an algebraic expression contains multiple operators, we associate s-vertices to each of them and arrange them from left to right, in the order they appear in the algebraic expression. If the position of some operators is ambiguous, they can be stacked verically. This is the case, for example, when two operators commute or are a part of the same normal product. Connections between lines inside a diagram correspond to contractions; thus drawing all possible connections between diagrams represents Wick theorem. As discussed earlier, when calculating the expectation value with respect to the true vacuum state, only the fully contracted contributions matters. In the diagrammatic representation, this means that the diagram must have no external lines. It also becomes necessary to somehow get the proper phase of a contraction directly from the diagram. To do this, we have to say that the parity of the number of intersections of fermion lines in a diagram is the same as the parity of the number of intersections in the pairing symbols. To see this, let us consider the following expression that can be evaluated using Eq. (1.3)

$$\langle 0|\, N[X_p X_q X_r] N[X_q^\dagger X_p^\dagger X_r^\dagger]\, |0\rangle = \langle 0|\, N[X_p X_q X_r X_q^\dagger X_p^\dagger X_r^\dagger]\, |0\rangle = 1.$$

This correspond to the following diagram:

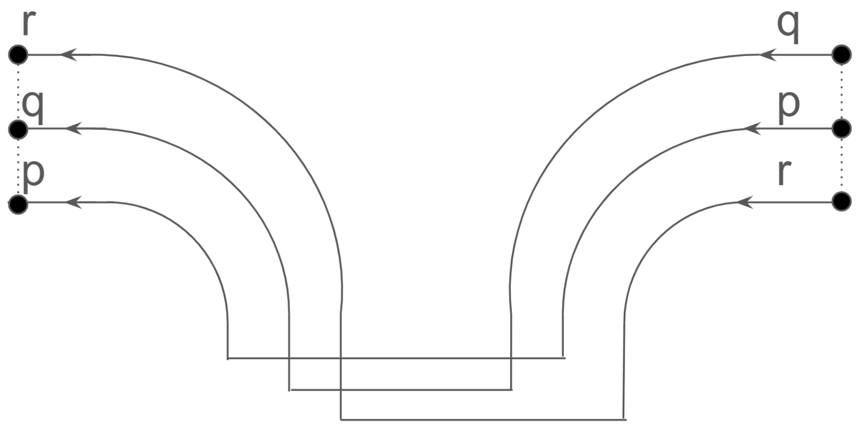

With this diagrammatic representation, there is clearly a correspondence between the number of crossings in the diagram and the algebraic expression. However, this diagram can be drawn in a more convenient way:

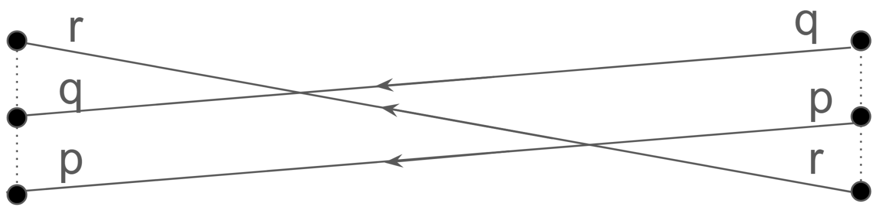

Importantly, the parity of the number of crossings stays the same. It can be shown that the parity of the number of crossings is independent of the way how a diagram is drawn.

From this point moving forward, we assume that all spin-orbital labels on diagrams are free labels, i.e., in the final expression, when we calculate the expectation value w.r.t. the physical vacuum state, we perform summations over the whole range of spin-orbitals for each free label appearing in the diagram.

A general operator $\hat{\Omega}$ associated with the diagram $\Omega(\omega)$, where ω designates the list of spin-orbital labels carried by the external lines, is given as:

$$\hat{\Omega} = w_\Omega \sum_\omega \hat{D}_\Omega(\omega),$$

where w_Ω is a numerical factor, discussed below, the summation extends over all external lines, and

$$\hat{D}_\Omega(\omega) = d_\Omega(\omega) \prod(\omega)$$

is the operator associated with the diagram $\Omega(\omega)$ with $d_\Omega(\omega)$ beeing the scalar factor and $\prod(\omega)$ designating the appropriate product of creation and anihilation operators.

We can also add internal lines: operator $\hat{\Omega}$ associated with the diagram $\Omega(\omega, \chi)$, where ω designates the list of spin-orbital labels carried by external lines and χ denotes labels carried by internal lines of $\hat{\Omega}$, is given as:

$$\hat{\Omega} = w_\Omega \sum_{\omega} \hat{D}_\Omega(\omega), \tag{1.4}$$

where

$$\hat{D}_\Omega(\omega) = \sum_{\chi} d_\Omega(\omega, \chi) \prod(\omega) \tag{1.5}$$

is the operator associated with the diagram $\Omega(\omega, \chi)$, $d_\Omega(\omega, \chi)$ is the scalar factor and summations in Eqs. (1.4) and (1.5) extend over all external and internal lines, respectively. $\prod(\omega)$ designates the appropriate product of creation and anihilation operators, and w_Ω is a numerical factor. We see that the only place where internal lines are present is the scalar factor $d_\Omega(\omega, \chi)$.

We now focus on determining the numerical factor w_Ω. As established, the diagram is a graph in which every fermion line carries a label. One defines the skeleton of a diagram as the diagram with all free labels removed. For example, the skeleton of the $\hat{V}$ operator has a form:

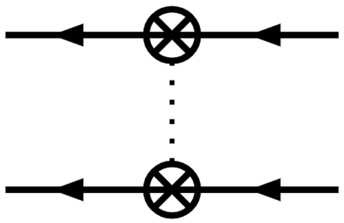

This skeleton has a pair of equivalent vertices attached to equivalent pairs of fermion lines, which can be transformed one into the other by symmetry operations. The same can be seen in the matrix element connected with the $\hat{V}$ diagram, i.e., $\langle pq|rs\rangle = \langle qp|sr\rangle$. This symmetry is related to the factor $\frac{1}{2}$ present in the final expression of $\hat{V}$:

$$\hat{V} = \frac{1}{2}\sum_{pqrs}\langle pq|rs\rangle X_p^\dagger X_q^\dagger X_s X_r.$$

In a more formal way, we can define a group $\mathcal{G}$ of automorphisms of the skeleton that do not change the left-right order of s-vertices; then, the weight factor is equal to $(\text{rank}(\mathcal{G}))^{-1}$. In the final expression for the evaluated quantity, we need to sum only over topologically non-equivalent skeletons. The importance of this requirement comes from the following theorem:

Let A, B, and R be skeletons with weights w_A, w_B, and w_R, respectively. Let R be the resulting skeleton obtained by joining a certain number of oriented lines of A and B pairwise. Then, the number M of distinct ways in which skeleton R can be obtained from skeletons A and B is given by

$$M = \frac{w_R}{w_A w_B}. \tag{1.6}$$

A consequence of this theorem is that when we include all possible skeletons R resulting from connecting skeletons A and B, we may count the same algebraical expression multiple times.

To summarize, to calculate the expectation value of a product of operators $\langle\hat{\Omega}_1\hat{\Omega}_2\ldots\hat{\Omega}_N\rangle$:

1. Draw all skeletons of diagrams corresponding to $\hat{\Omega}_1, \hat{\Omega}_2, \ldots, \hat{\Omega}_N$ from left to right.

2. Draw all resulting non-equivalent R-skeletons, which are obtained by connecting all external lines of skeletons $\Omega_1, \Omega_2, \ldots, \Omega_N$ such that the R-skeletons have no external lines.

3. Label all lines in R-skeletons and find the appropriate scalar factor d_R, which is obtained as:

$$d_R(r,\chi_r) = w_R(r)(-1)^{n_{\text{is}}} \prod_{i=1}^{N} d_{\Omega_i}(\omega_i)\delta_{\omega_i\chi_r},$$

where

- r enumerates which of non-equivalent $R-$skeletons is considered,
- $w_R(r)$ is the weight of the resulting diagram,
- n_{is} is the number of intersections of fermion lines in the diagram,
- $\prod_{i=1}^{N} d_{\Omega_i}(\omega_i)\delta_{\omega_i\chi_r}$ represents a product of all scalar parts of $\hat{D}_{\Omega_i}$ operators with appropriate contractions due to Wick theorem. The notation $\delta_{\omega_i\chi_r}$ indicates that the set of external indices appearing in the diagram of the $\hat{\Omega}_i$ operator is a subset of internal indices of the R-diagram and effectively represents a particular contraction scheme.

4. Get the final expression:

$$\langle \hat{\Omega}_1\hat{\Omega}_2 \ldots \hat{\Omega}_N \rangle = \sum_r \sum_{\chi_r} d_R(r,\chi_r),$$

where $\sum_{\chi_r}$ denotes the summation over all internal labels of lines.

2 Diagrammatic representation of arbitrary geminals

We begin by defining the k-th geminal depending on a full set of coordinates of two electrons, $1 \equiv (\mathbf{r}_1, \sigma_1)$ and $2 \equiv (\mathbf{r}_2, \sigma_2)$ as:

$$g^{(k)}(1,2) = \sum_{i_k,j_k} A^{(k)}_{i_k j_k}\, \varphi_{i_k}(\mathbf{r}_1)\varphi_{j_k}(\mathbf{r}_2)\, \chi(\sigma_1,\sigma_2), \tag{2.1}$$

where $A^{(k)}_{i_k j_k}$ are geminal expansion coefficients, $\varphi_i(\mathbf{r})$ are one-electron spatial functions and $\chi(\sigma_1, \sigma_2)$ is the antisymmetric singlet spin function,

$$\chi(\sigma_1, \sigma_2) = \alpha(\sigma_1)\beta(\sigma_2) - \beta(\sigma_1)\alpha(\sigma_2).$$

To assure the antisymmetry of the geminal with respect to exchange of both spatial and spin coordinates of electrons,

$$g^{(k)}(1,2) = -g^{(k)}(2,1),$$

the coefficients $A^{(k)}_{i_k j_k}$ must form a symmetric matrix. Then, we define an N-geminal wave function for a $2N$-electron system as:

$$\begin{aligned} |\Phi\rangle &= \mathcal{N}\hat{\mathcal{A}} \prod_{k=1}^{N} g^{(k)}(2k-1, 2k) \\ &= \mathcal{N} \sum_{\substack{i_1 i_2 \cdots i_N \\ j_1 j_2 \cdots j_N}} \left(\prod_{k=1}^{N} A^{(k)}_{i_k j_k} \right) \hat{\mathcal{A}} \prod_{k=1}^{N} \Big([\varphi_{i_k}\alpha](2k-1)\, [\varphi_{j_k}\beta](2k) - [\varphi_{i_k}\beta](2k-1)\, [\varphi_{j_k}\alpha](2k) \Big), \end{aligned}$$

where $\mathcal{N}$ is an arbitrary constant, and $\hat{\mathcal{A}} = \sum_P (-1)^p P$ is the antisymmetrizer that sums over all permutations P of the S_{2N} group with the parity p. To arrive at the second-quantized wave function Ansatz, we define the operator $G^\dagger(i,j)$ as:

$$G^\dagger(i,j) = X^\dagger_{i\alpha} X^\dagger_{j\beta} - X^\dagger_{i\beta} X^\dagger_{j\alpha},$$

where $X^\dagger_{i\mu}$ is the creation operator for the $[\varphi_i \mu]$ spinorbital. With this definition, we have:

$$|\Phi\rangle = \mathcal{N} \sum_{\substack{i_1 i_2 \cdots i_N \\ j_1 j_2 \cdots j_N}} \prod_{k=1}^{N} A^{(k)}_{i_k j_k} G^\dagger(i_k, j_k) \, |0\rangle \,.$$

Now, we can split the $G^\dagger$ operator into two parts,

$$G^\dagger(i,j) = F^{(0)\dagger}(i,j) + F^{(1)\dagger}(i,j), \tag{2.2}$$

where for $r = 0, 1$ we have:

$$F^{(r)\dagger}(i,j) = (-1)^r X^\dagger_{i\mu(r)} X^\dagger_{j\mu(1-r)},$$

using the notation $\mu(0) = \alpha$, $\mu(1) = \beta$. This gives four types of F operators:

$$\begin{aligned}
F^{(0)\dagger}(i,j) &= \ \ X^\dagger_{i\alpha} X^\dagger_{j\beta}, \\
F^{(1)\dagger}(i,j) &= -X^\dagger_{i\beta} X^\dagger_{j\alpha}, \\
F^{(0)}(i,j) &= \ \ X_{j\beta} X_{i\alpha}, \\
F^{(1)}(i,j) &= -X_{j\alpha} X_{i\beta}.
\end{aligned}$$

With these operators, we can write the geminal wave function as:

$$|\Phi\rangle = \mathcal{N} \sum_{r_1,r_2,\ldots,r_N=0}^{1} \sum_{\substack{i_1 i_2 \ldots i_N \\ j_1 j_2 \ldots j_N}} \prod_{k=1}^{N} A^{(k)}_{i_k j_k} F^{(r_k)\dagger}(i_k, j_k) \, |0\rangle \, . \tag{2.3}$$

In this way, we split the wave function into a linear combination of 2^N states which differ in sequences of the $F^{(0)\dagger}$ and $F^{(1)\dagger}$ operators.

To construct the diagrammatic representation of a geminal, we first introduce a set of s-vertices:

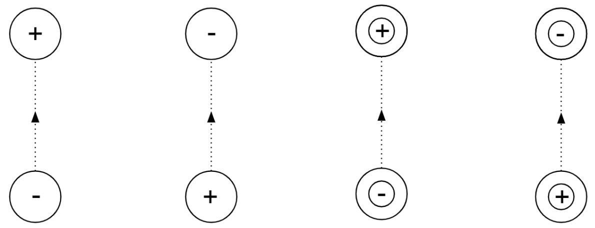

which, going from left to right, correspond to $F^{(0)\dagger}$, $F^{(1)\dagger}$, $F^{(0)}$ and $F^{(1)}$ operators, respectively. Next, we combine the s-vertices with oriented fermion lines to obtain F-skeletons and the corresponding diagrams:

- $F^{(0)}$ skeleton and the example of the $F^{(0)}$ diagram:

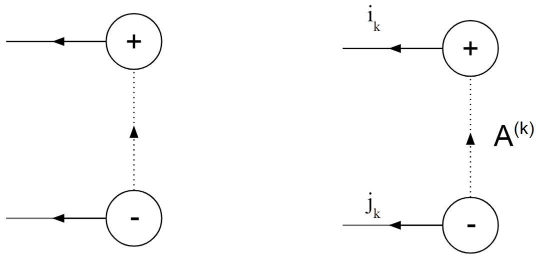

- $F^{(1)}$ skeleton and $F^{(1)}$ diagram:

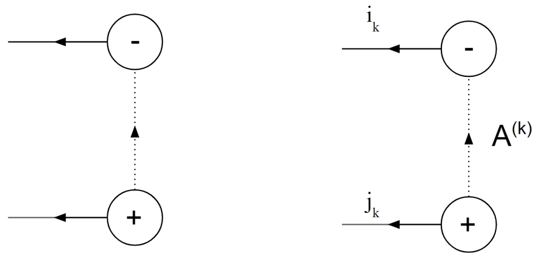

- $\tilde{F}^{(0)}$ skeleton and $\tilde{F}^{(0)}$ diagram:

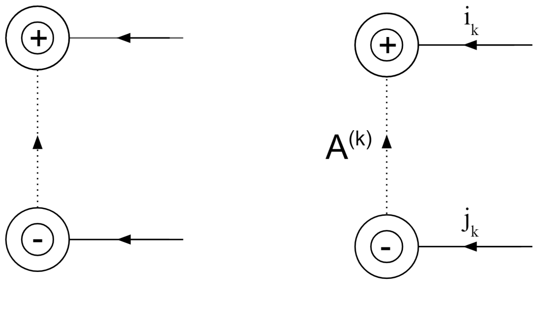

- $\tilde{F}^{(1)}$ skeleton and $\tilde{F}^{(1)}$ diagram:

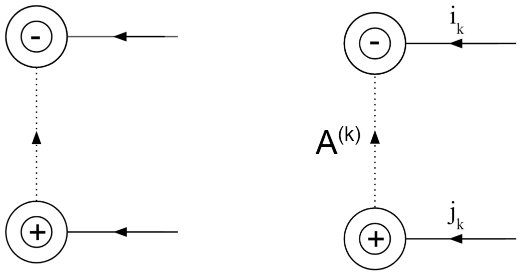

To connect these diagrams with algebraic expressions, we use the following rules:

1. The scalar quantity associated with the diagram X, where X is either $F^{(r)}$ or $\tilde{F}^{(r)}$, which has a matrix of geminal coefficients $A^{(k)}$ and orbital indices $\chi = (i_k, j_k)$, is given by $d_X(A^{(k)}, \chi) = (-1)^{r+s} A^{(k)}_{i_k j_k}$. Specifically, if $r = 0$ the dashed line is oriented toward the (+) sign, and if $r = 1$ the dashed line is oriented toward the (−) sign. The value of s depends on the direction of the dashed line: $s = 0$ if it is directed upwards, and $s = 1$ if it is directed downwards.

2. The operator part associated with the diagram comes from the following rules:

   - Each solid oriented line corresponds to either a creation or an anihilation operator.
   - Lines outgoing from a vertex represent creation operators, whereas lines incoming to a vertex represent anihilation operators.
   - An operator with spin α is connected to the (+) side of the s-vertex, whereas an operator with spin β is connected to the (−) side of the s-vertex.
   - Operators are written in the order from top to bottom for $F^{(r)}$ diagrams, and in bottom-to-top order for $\tilde{F}^{(r)}$ diagrams.

As a check, let us write down final algebraic expressions resulting from four basic diagrams shown above.

- $F^{(0)}$ diagram:

  1. $r = 0$
  2. $s = 0$
  3. Operator part: $X^\dagger_{i_k\alpha} X^\dagger_{j_k\beta} = N[X^\dagger_{i_k\alpha} X^\dagger_{j_k\beta}]$
  4. Final expression: $A^{(k)}_{i_k j_k} X^\dagger_{i_k\alpha} X^\dagger_{j_k\beta} = A^{(k)}_{i_k j_k} F^{(0)\dagger}(i_k, j_k)$

- $F^{(1)}$ diagram:

1. $r = 1$
2. $s = 0$
3. Operator part: $X^\dagger_{i_k\beta} X^\dagger_{j_k\alpha} = N[X^\dagger_{i_k\beta} X^\dagger_{j_k\alpha}]$
4. Final expression: $A^{(k)}_{i_k j_k}(-1) X^\dagger_{i_k\beta} X^\dagger_{j_k\alpha} = A^{(k)}_{i_k j_k} F^{(1)\dagger}(i_k, j_k)$

- $\tilde{F}^{(0)}$ diagram:

1. $r = 0$
2. $s = 0$
3. Operator part: $X_{j_k\beta} X_{i_k\alpha} = N[X_{j_k\beta} X_{i_k\alpha}]$
4. Final expression: $A^{(k)}_{i_k j_k} X_{j_k\beta} X_{i_k\alpha} = A^{(k)}_{i_k j_k} F^{(0)}(i_k, j_k)$

- $\tilde{F}^{(1)}$ diagram:

1. $r = 1$
2. $s = 0$
3. Operator part: $X_{j_k\alpha} X_{i_k\beta} = N[X_{j_k\alpha} X_{i_k\beta}]$
4. Final expression: $A^{(k)}_{i_k j_k}(-1) X_{j_k\alpha} X_{i_k\beta} = A^{(k)}_{i_k j_k} F^{(1)}(i_k, j_k)$

Formally, all the diagrams above should be called $F(1)$ and $\tilde{F}(1)$ diagrams, because they represent a part of a single geminal. A set of N such parts is then called $F(N)$ or $\tilde{F}(N)$ diagram.

Notice that all diagrams can be drawn in an "upside-down" way. Consider, for example, the $F^{(1)}$ diagram drawn as:

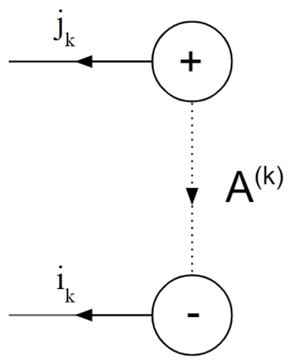

In this case, we have: $r = 1$, $s = 1$, operator: $X^{\dagger}_{j_k\alpha}X^{\dagger}_{i_k\beta}$, and the final expression is $A^{(k)}_{j_k i_k}X^{\dagger}_{j_k\alpha}X^{\dagger}_{i_k\beta} = A^{(k)}_{i_k j_k}(-1)X^{\dagger}_{i_k\beta}X^{\dagger}_{j_k\alpha} = A^{(k)}_{i_k j_k}F^{(1)\dagger}(i_k, j_k)$. We get exactly the same operator as proposed earlier for the $F^{(1)}$ s-vertex. Therefore, these diagrams/skeletons represent exactly the same operator. The significance of the s number becomes clear at this point—it gives the proper order of operators even if we switch our diagram "upside-down".

To build a diagram which represents an N-geminal wave function, we should use all possible 2^N combinations of $F^{(0)}$ and $F^{(1)}$ diagrams [see Eq. (2.3)]. However, we will show how to simplify the evaluation of expectation values with respect to a N-geminal wave function $|\Phi\rangle$ with the use of a new type of diagrams that we will call G diagrams.

Let us start with an example. Suppose that we want to calculate the norm of the k-th geminal, i.e., of the two-electron (one-geminal) wave function $|\Phi_k\rangle$. We have:

$$|\Phi_k\rangle = \mathcal{N}_k \sum_{ij} A^{(k)}_{ij} \left(F^{(0)\dagger}(i,j) + F^{(1)\dagger}(i,j)\right)|0\rangle \tag{2.4}$$

and

$$\langle\Phi_k| = \mathcal{N}_k \langle 0| \sum_{ij} A^{(k)}_{ij} \left(F^{(0)}(i,j) + F^{(1)}(i,j)\right).$$

To calculate $\langle\Phi_k|\Phi_k\rangle$, we need to evaluate four distinct terms, each involving one of the following operator pairs: $F^{(0)}F^{(0)\dagger}$, $F^{(0)}F^{(1)\dagger}$, $F^{(1)}F^{(0)\dagger}$, and $F^{(1)}F^{(1)\dagger}$. Consider only the $F^{(0)}F^{(0)\dagger}$ part:

$$\mathcal{N}_k^2 \langle 0| \left[\sum_{ij} A^{(k)}_{ij} F^{(0)}(i,j)\right]\left[\sum_{mn} A^{(k)}_{mn} F^{(0)\dagger}(m,n)\right]|0\rangle .$$

Using the definitions of the F operators, it can be rewritten as:

$$\mathcal{N}_k^2 \sum_{ij} \sum_{mn} A_{ij}^{(k)} A_{mn}^{(k)} \langle 0| N[X_{j\beta} X_{i\alpha}] N[X^\dagger_{m\alpha} X^\dagger_{n\beta}] |0\rangle .$$

Applying Wick theorem, we see that there is only one possible contraction:

$$\langle 0| N[X_{j\beta} X_{i\alpha}] N[X^\dagger_{m\alpha} X^\dagger_{n\beta}] |0\rangle = \langle 0| N[\overbrace{X_{j\beta} X_{i\alpha} X^\dagger_{m\alpha} X^\dagger_{n\beta}}] |0\rangle = (-1)^2 \delta_{jn} \delta_{im},$$

which leads us to:

$$\mathcal{N}_k^2 \sum_{ij} \sum_{mn} A_{ij}^{(k)} A_{mn}^{(k)} \langle 0| N[X_{j\beta} X_{i\alpha}] N[X^\dagger_{m\alpha} X^\dagger_{n\beta}] |0\rangle = \mathcal{N}_k^2 \sum_{ij} \left(A_{ij}^{(k)} \right)^2 .$$

This result can be also obtained using F diagrams. We want to calculate the expectation value w.r.t. true vacuum state. To do this, we will use the rules described in section 1, which we will adapt to the geminal problem.

The diagrammatic representation of Wick theorem using F diagrams can be constructed as follows. Contractions are represented by connections between solid oriented lines. There is a one-to-one correspondence between the number of intersections of these lines and the number of intersections of the pairing symbols in the corresponding normal product. Given this, we can conclude that the total sign factor is:

$$(-1)^{C+D+E}, \tag{2.5}$$

where

- C is the number of crossings of solid oriented lines,
- D is the number of dashed lines inside s-vertices oriented downward,
- E is the number of dashed lines inside s-vertices oriented toward the $(-)$ sign (i.e., the number of $F^{(1)}$ and $\tilde{F}^{(1)}$ vertices).

Moreover, we have to remember that lines which are connected to the $(+)$ sign are always α-lines, whereas lines connected to the $(-)$ sign are always β-lines, which limits the number of proper connections/contractions, due to fact that $\overline{X_{i\mu}X^{\dagger}_{j\nu}} = \delta_{ij}\delta_{\mu\nu}$.

We are now ready to evaluate the $F^{(0)}F^{(0)\dagger}$ part of the $\langle\Phi_k|\Phi_k\rangle$ norm using the diagrammatic approach. First, we draw skeletons of $\tilde{F}^{(0)}$ and $F^{(0)}$ diagrams (corresponding to $F^{(0)}$ and $F^{(0)\dagger}$ operators, respectively), then connect them in all possible ways. Then, we name solid oriented lines (here, we gave them i, j indices).

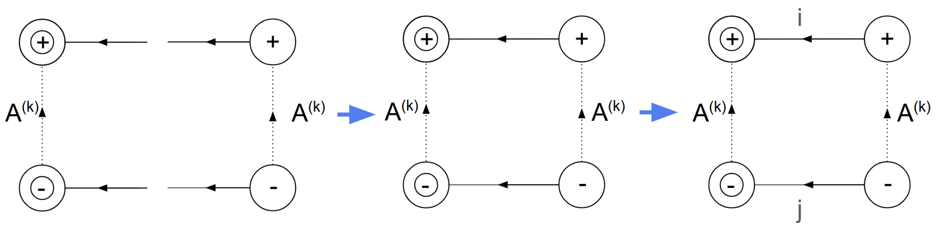

Next, we calculate the sign factor: $(-1)^{0+0+0}$. The scalar factor is taken as a product of geminal coefficient from both vertices: $A^{(k)}_{ij}A^{(k)}_{ij}$. Finally, we sum over all possible indices:

$$\sum_{ij}\left(A^{(k)}_{ij}\right)^2$$

If needed, we can multiply this result by the normalization factor connected to the diagram (here $\mathcal{N}_k^2$).

With the diagrammatic representation, it becomes easy to see that all remaining terms, $F^{(0)}F^{(1)\dagger}$, $F^{(1)}F^{(0)\dagger}$, and $F^{(1)}F^{(1)\dagger}$ give exactly the same scalar result, $\sum_{ij}\left(A^{(k)}_{ij}\right)^2$. These skeletons look as follows:

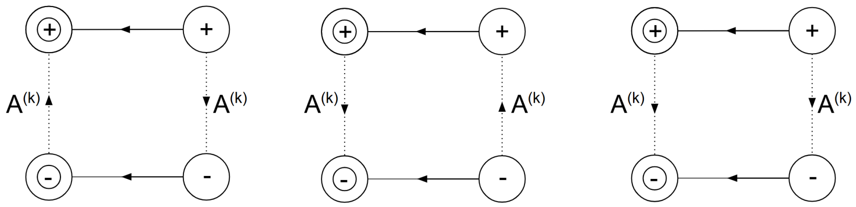

As we can see, each of the four components gives the same contribution to the final expression.

Next, we generalize this observation to wavefunctions with an arbitrary number of geminals. Consider a diagram build of N s-vertices of the $F^{(0)}$ type. The operator associated with this diagram is expressed as:

$$\hat{D}_F(\{0_N\}, V, \chi) = \prod_{k=1}^{N} A^{(k)}_{i_k,j_k} F^{(0)\dagger}(i_k, j_k)$$

where $\{0_N\}$ is a string of N zeros which indicates that we took only $F^{(0)}$ diagrams, so that in the final expression we get only $F^{(0)\dagger}$ operators, $V = (A^{(1)}, A^{(2)}, \cdots, A^{(N)})$ is the set of geminals coefficients, and $\chi = (i_1, j_1; i_2, j_2; \ldots; i_N, j_N)$ is the set of indices. When we replace one $F^{(0)}$ diagram with an $F^{(1)}$ diagram, we obtain a new $\hat{D}_F$ operator associated with a different binary string, which we denote as $\{0_{N-1}1_1\}_t$, where t indicates which $F^{(0)}$ s-vertex was changed to $F^{(1)}$. In other words, t enumerates which of the binary strings containing exactly one 1 is chosen. Now, we can change another $F^{(0)}$ s-vertex to $F^{(1)}$, generating $N(N-1)/2$ new diagrams. This process can be continued until all possible 2^N diagrams are obtained. Using all these diagrams, we can define an N-geminal wave function:

$$|\Phi\rangle = \mathcal{N} \sum_{i=1}^{N} \sum_{t=1}^{\binom{N}{i}} \sum_{\chi} \hat{D}_F(\{0_{N-i}1_i\}_t, V, \chi) |0\rangle .$$

Using this formula we can express the expectation value of an arbitrary operator $\hat{\Omega}$ as:

$$\langle\Phi| \hat{\Omega} |\Phi\rangle = \mathcal{N}^2 \sum_{j=1}^{N} \sum_{s=1}^{\binom{N}{j}} \sum_{\tilde{\chi}} \sum_{i=1}^{N} \sum_{t=1}^{\binom{N}{i}} \sum_{\chi} \sum_{\omega} \langle 0| \hat{D}_{\tilde{F}}(\{0_{N-j}1_j\}_s, \tilde{V}, \tilde{\chi}) \hat{D}_{\Omega}(\omega) \hat{D}_F(\{0_{N-i}1_i\}_t, V, \chi) |0\rangle ,$$

where $\hat{\Omega}$ can be associated with diagram Ω with spinorbital labels ω. Naturally, to obtain $\langle\Phi|\hat{\Omega}|\Phi\rangle$, we must consider all possible $F(N)$ and $\tilde{F}(N)$ diagrams (2^{2N} possibilities) and sum over all resulting diagrams. However, we observe that one diagram can be trivially transformed into another simply by reversing the arrow direction inside the s-vertex. In this way, starting from a single diagram, we can generate 2^{2N} distinct diagrams. Each of these diagrams yields the same scalar factor and the same sign. The sign remains unchanged

because both the number of s-vertex lines oriented downward and the number of s-vertex lines oriented toward the $(-)$ sign change by one simultaneously [i.e., both D and E in Eq. (2.5) change by one]. We can say that:

$$\langle\Phi|\,\hat{\Omega}\,|\Phi\rangle = \mathcal{N}^2 2^{2N} \sum_{\tilde{\chi}} \sum_{\chi} \sum_{\omega} \langle 0|\,\hat{D}_{\tilde{F}}(\{0_N\},\tilde{V},\tilde{\chi})\,\hat{D}_{\Omega}(\omega)\,\hat{D}_F(\{0_N\},V,\chi)\,|0\rangle\,. \tag{2.6}$$

Now we focus on obtaining that quantity in the diagrammatic representation.

We begin by introducing G skeletons, which are obtained from F skeletons by replacing F s-vertices with closed dots. This is motivated by the fact that $F^{(0)}$ and $F^{(1)}$ diagrams differ solely in the direction of the s-vertex line and, as we discussed it previously, both represent the same scalar quantity. Similarly, we define $\tilde{G}$ vertices by replacing $\tilde{F}$ s-vertices with open dots. From this point onward, we omit arrows in diagrams. This is because we apply Wick theorem with respect to the physical vacuum, so all arrows must consistently point from the right to the left. We observe that a closed dot can only be connected to either the interaction operator or an open dot, whereas an open dot can be connected to either the interaction operator or a closed dot. Below, we present an example of the diagrammatic representation of a $G(N)$ skeleton and diagram containing N different G vertices, each representing a different geminal. From now on we will label G vertices with symbol k instead of $A^{(k)}$.

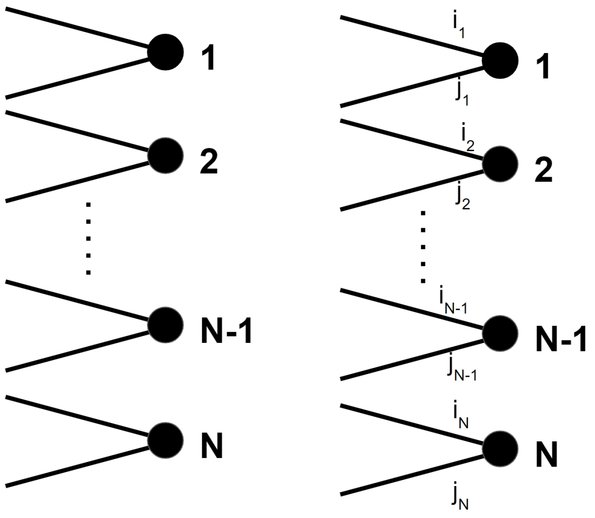

To this diagram we assign scalar factor:

$$d_G(V,\chi) = \prod_{k=1}^{N} A^{(k)}_{i_k,j_k},$$

where V is a set of vertex coefficients, $V = (A^{(1)}, A^{(2)}, \ldots, A^{(N)})$, and χ is a set of line labels, $\chi = (i_1, j_1; i_2, j_2; \ldots, i_N, j_N)$. The operator associated with that diagram reads:

$$\hat{D}_G(V,\chi) = d_G(V,\chi) \prod_{k=1}^{N} G^{\dagger}(i_k, j_k). \tag{2.7}$$

With that operator we can define operator $\hat{W}$:

$$\hat{W} = w_G \sum_{\chi} \hat{D}_G(V,\chi),$$

where w_G is the weight of the diagram, i.e., the inverse of the number of automorhpisms of its skeleton. In this case (N different geminals): $w_G = 2^{-N}$. This is due to equivalency of lines in each geminal. With this definition, the N-geminal wave function reads:

$$|\Phi\rangle = \hat{W}\,|0\rangle$$

The recipe to obtain $\langle\Phi|\,\hat{\Omega}\,|\Phi\rangle$ with the use of G diagrams is the following.[S6]

1. Assign skeletons to the $\hat{\Omega}$ operator, and $\langle\Phi|$ and $|\Phi\rangle$ states.

2. Form all possible topologically different skeletons R_G, and determine their weights $w_R(r)$, where r enumerates the resulting R_G skeletons.

3. Label all the lines of a skeleton to obtain R_G diagrams. The sets of line indices are denoted χ_r.

4. Assign scalar quantities, $d_R(r, \chi_r)$, to these diagrams.

    - Count the number of $\tilde{G}$ vertices, denoted n, inside the diagram.

- Count the number of loops, denoted l, inside the diagram.
- Assign the scalar $d(\chi_r) = d_{\tilde{G}}(\tilde{V}, \tilde{\chi})d_\Omega(\omega)d_G(V, \chi)\,\delta_{(\tilde{\chi},\omega,\chi),\chi_r}$ which includes all geminal expansion coefficients as well as the scalar factor associated with the operator $\hat{\Omega}$, all contracted due to Kronecker deltas coming from Wick theorem.
- The final quantity associated with that diagram is equal to:

$$d_R(r, \chi_r) = (-1)^{n+l}\,2^l\,w_R(r)d(\chi_r).$$

The factor 2^l comes from fact that final expression is summed over spin.

5. Calculate the final expression as:

$$\langle\Phi|\,\hat{\Omega}\,|\Phi\rangle = \sum_r \sum_{\chi_r} d_R(r, \chi_r).$$

As a final remark, note that the standard left-right skeletons can be replaced with cyclic skeletons. For example:

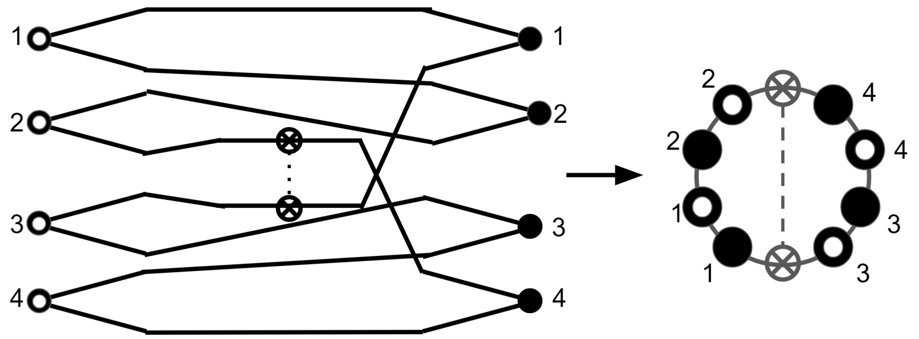

Of course, left-right skeleton contain exactly the same information as the cyclic one. We return to cyclic diagrams in Section 5 when introducing the diagrammatic approach to SAPT(APSG).

In the next section, we discuss further simplifications of the diagrammatic approach in the specific case of the APSG wave function.

2.1 Example

Let us evaluate the diagrammatic example from the previous section. To do this, we draw both the skeleton and the corresponding diagram:

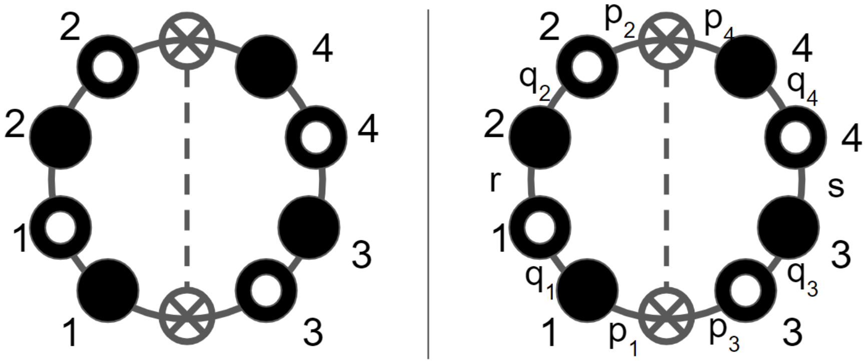

We then gather all quantities needed to evaluate such a diagram: the number of loops $l = 1$, the number of $\tilde{G}$ vertices $n = 4$. Since there are no equivalent lines, $w_R = 1$. Scalar $d(\chi)$ connected with this diagram:

$$d(\chi) = \left(A^{(1)}_{p_1q_1} A^{(1)}_{q_1r} A^{(2)}_{rq_2} A^{(2)}_{q_2p_2}\right) \left(A^{(4)}_{p_4q_4} A^{(4)}_{q_4s} A^{(3)}_{sq_3} A^{(3)}_{q_3p_3}\right) (p_2p_4|p_3p_1),$$

where $\chi = (p_1, p_2, p_3, p_4, q_1, q_2, q_3, q_4, r, s)$ denotes orbital indices. The final quantity connected with the diagram is equal to:

$$\sum_{\chi} (-1)^{n+l}\, 2^l\, w_R\, d(\chi) = -2 \sum_{\substack{p_1p_2p_3p_4\\ q_1q_2q_3q_4\\ rs}} \left(A^{(1)}_{p_1q_1} A^{(1)}_{q_1r} A^{(2)}_{rq_2} A^{(2)}_{q_2p_2}\right) \left(A^{(4)}_{p_4q_4} A^{(4)}_{q_4s} A^{(3)}_{sq_3} A^{(3)}_{q_3p_3}\right) (p_2p_4|p_3p_1).$$

3 Simplifications for APSG wave function

In the ASPG theory,[S8,S9] we assume that the $2N$-electron wave function is built from geminals

$$|\Phi\rangle = 2^{-N} \hat{\mathcal{A}} \prod_{I=1}^{N} g^{(I)}(2I-1, 2I),$$

which are strongly orthogonal, i.e., for $K \neq L$:

$$\int \mathrm{d}1\, g^{(K)}(1,2) g^{(L)}(1,3) = 0,$$

and each geminal can be expanded in natural orbitals:

$$g^{(K)}(1,2) = \sum_{i=1}^{N_K} c_i^K \varphi_i^K(\mathbf{r}_1) \varphi_i^K(\mathbf{r}_2) \chi(\sigma_1, \sigma_2),$$

where N_K denotes the number of orbitals in geminal K. In the case of GVB-PP, we have $N_K = 2$ for all K.[S10] Geminals are normalized according to:

$$\sum_{i=1}^{N_K} (c_i^K)^2 = 1.$$

In the following we will also use occupation numbers defined as $n_i^K = (c_i^K)^2$. In view of the Arai theorem,[S11] we can say that orbitals belonging to different geminals are orthogonal

$$\int \mathrm{d}\mathbf{r}\, \varphi_i^K(\mathbf{r}) \varphi_j^L(\mathbf{r}) = \delta_{KL} \delta_{ij}.$$

Let us introduce an alternative convention for indexing: all orbitals forming all geminals are enumerated continuously, and a group of orbitals belonging to a given geminal is collectively referred to by a separate geminal index. Thus, if orbital i belongs to geminal K, we write $i \in I_K$. For example, if geminal 4 consists of orbitals 4 and 7, we denote this as $I_4 = \{4, 7\}$ notation. In this formulation:

$$g^{(K)}(1,2) = \sum_{i \in I_K} c_i \varphi_i(\mathbf{r}_1) \varphi_i(\mathbf{r}_2) \chi(\sigma_1, \sigma_2).$$

We omit the superscript K, because it is clear that orbital i belongs to geminal K.

In the second-quantized form we can represent K geminal as:

$$\sum_{i \in I_K} c_i X^\dagger_{i\alpha} X^\dagger_{i\beta} \left|0\right\rangle ,$$

which can be rewritten as:

$$\sum_{i \in I_K} c_i X^\dagger_{i\alpha} X^\dagger_{i\beta} \left|0\right\rangle = \frac{1}{2} \sum_{i \in I_K} c_i \left(X^\dagger_{i\alpha} X^\dagger_{i\beta} - X^\dagger_{i\beta} X^\dagger_{i\alpha} \right) \left|0\right\rangle = \frac{1}{2} \sum_{i \in I_K} c_i G^\dagger(i,i) \left|0\right\rangle .$$

We can now see the correspondence to the previous representation of a geminal, given in Eq. (2.4): the $A^{(k)}_{ij}$ matrix becomes diagonal in the natural orbital representation, i.e., $A^{(k)}_{ij} = c_i \delta_{ij}$ if $i \in I_K$. The diagrammatic representation of a geminal vertex can be supplemented with a condition that both lines, whether outgoing or incoming to the $G/\tilde{G}$ vertex, share the same orbital index.

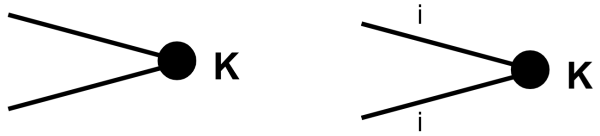

Moreover, due to the strong orthogonality condition, all diagrams in which distinct geminal vertices are directly connected, give zero contributions. For $K \neq L$ this can be schematically represented as:

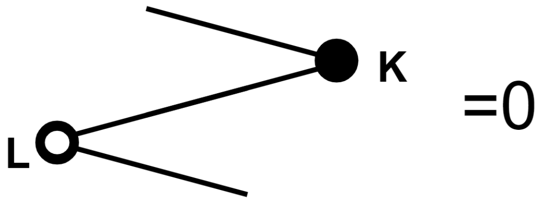

Additionally, we can easily check the norm of single geminal:

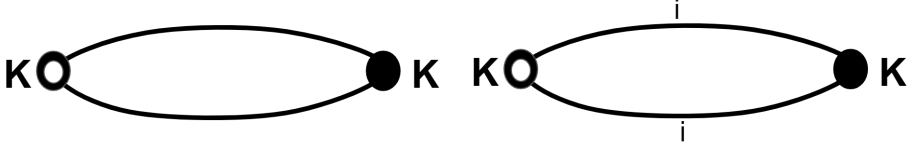

From the left skeleton, the weight of this skeleton is $w_R = \frac{1}{2}$ due to the equivalence of lines. The number of $\tilde{G}$ vertices is $n = 1$ and the number of loops is $l = 1$. The scalar $d(\chi)$ associated with this diagram is

$$d(\chi) = c_i^2,$$

where $i \in I_K$. Thus, the final scalar expression corresponding to the diagram is:

$$\sum_{\chi}(-1)^{n+l}\, 2^l\, w_R\, d(\chi) = \sum_{i\in I_K} c_i^2 = 1.$$

This confirms that the norm of a geminal is equal to one. We will refer to such a diagram as the normalization loop.

This knowledge is particularly useful when combined with the fact that the entrie expectation value diagram can be decomposed into loops. Each loop's scalar expression can be derived individually, and the final result is obtained by taking the product of these expressions.

In view of the strong orthogonality condition we know that, if we calculate the expectation value of $\hat{\Omega}$, all non-vanishing diagrams will have the property:

For $K = 1, 2, ..., N$, the $\tilde{G}$ vertex of geminal K is connected directly either to the G vertex of geminal K, or to the $\hat{\Omega}$ vertex.

Because of this, we can split the geminal indices in each diagram into two parts. The first part is directly connected to the $\hat{\Omega}$ vertex, while the second part consists of geminals that form the normalization loops. Since we know that the second part contributes only a factor of 1 to the final expression, we will no longer draw these diagrams; though we keep in mind that they are present in this theory. For example, when we want to calculate the expectation value of a one-body operator $\hat{h}_1$:

$$\hat{h}_1 = \sum_{pq\mu} h_{pq} X^{\dagger}_{p\mu} X_{q\mu},$$

represented as:

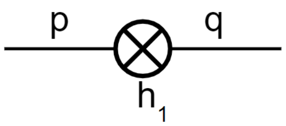

we calculate the expectation value $\langle\Phi|\hat{h}_1|\Phi\rangle$ which is represented as:

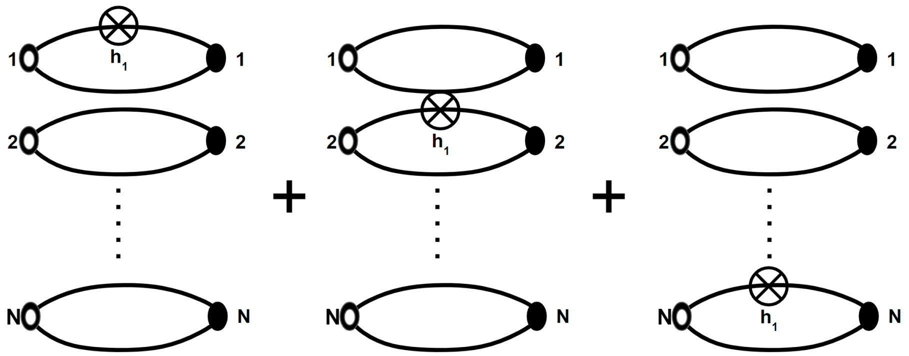

Using the simplification described above, we can redraw this as:

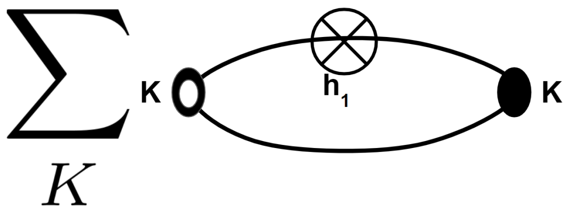

The next step is to use bare diagrams/skeletons instead of vertex-labeled ones. In this case, we start by drawing the skeleton without naming the vertices. Then, when creating diagrams, we assign names to both vertices and fermion lines and sum over all possible vertices and lines. For example, all the skeletons above can be represented by a single skeleton:

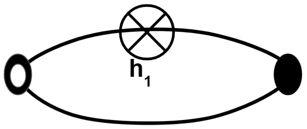

In this case, however, we have to be more careful with the weight factor, because there can be higher symmetry due to the indistinguishability of vertices.

3.1 Example

Let us return to the evaluation of the $\langle\Phi|\,\hat{h}_1\,|\Phi\rangle$ value. When working with vertex-labeled diagrams/skeletons, we begin by drawing N different skeletons, each corresponding to the interaction operator being connected to geminals $K = 1, 2, \ldots, N$. Now, let us determine the quantity associated with the diagram where the interaction occurs in the K-th geminal. To do this, we first draw the corresponding skeleton and diagram:

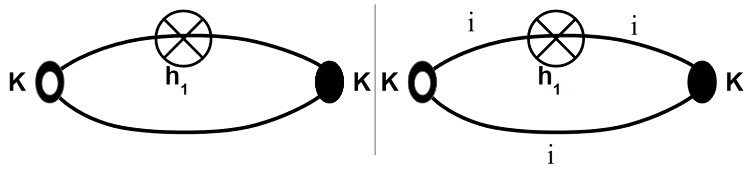

Since it is an APSG geminal, we see that the lines outgoing from and ingoing to the geminal vertices both have the index i . Now, we can determine all the quantities needed to evaluate the diagram. First, $w_R = 1$ because there are no equivalent lines. The number of $\tilde{G}$ vertices is $n = 1$ and the number of loops is $l = 1$. The scalar associated with this diagram is given by:

$$d(\chi) \equiv d(i) = c_i^2 h_{ii} = n_i h_{ii},$$

where $i \in I_K$. The final expression connected with the K-th diagram has the form:

$$\sum_{\chi} (-1)^{n+l}\, 2^l\, w_R\, d(\chi) = 2 \sum_{i \in I_K} n_i h_{ii}.$$

Here, notice that we sum over <u>orbital indices</u>. To obtain the final expression, we must sum over all N diagrams:

$$\langle\Phi|\,\hat{h}_1\,|\Phi\rangle = 2 \sum_{K=1}^{N} \sum_{i \in I_K} n_i h_{ii}$$

The same result can be obtained using unlabeled vertices. In this case, we draw only one skeleton and one diagram instead of N diagram, as in the labeled case:

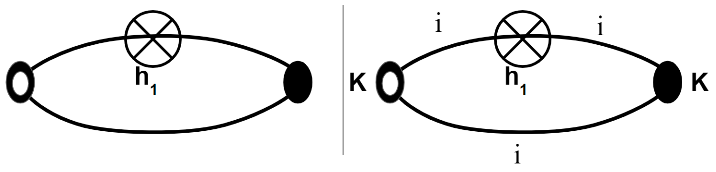

Here, both the vertices and lines of a skeleton must be labeled to obtain a diagram. We see that the index K appears on both sides because the geminals are connected by a fermion line. Due to the strong orthogonality condition, it must be the same geminal. Now, we calculate the required quantities: $w_R = 1$, $n = 1$, $l = 1$, and

$$d(\chi) \equiv d(K,i) = c_i^2 h_{ii} = n_i h_{ii},$$

where $i \in I_K$. Thus, the value associated with this diagram is:

$$\sum_{\chi} (-1)^{n+l}\, 2^l\, w_R\, d(\chi) = 2 \sum_K \sum_{i \in I_K} n_i h_{ii}.$$

Notice that this time the sum runs over vertices and orbital indices. As this was only possible skeleton/diagram, we obtain:

$$\langle \Phi | \, \hat{h}_1 \, | \Phi \rangle = 2 \sum_K \sum_{i \in I_K} n_i h_{ii}.$$

Even this simple example illustrates the advantage of using skeletons with unlabeled vertices: instead of drawing N different skeletons, we only need to draw one.

4 Diagrammatic representation in tensor product of Fock-spaces

In this section we generalize the introduced diagrammatic approach to a tensor product of monomer Fock spaces, $\mathcal{F}_A \otimes \mathcal{F}_B$. We use two sets of creation/anihilation operators: $a_p^\dagger$, a_q

and $b_r^\dagger$, b_s, acting on $\mathcal{F}_A$ and $\mathcal{F}_B$, respectively. They obey the standard anticommutation relations in spaces $\mathcal{F}_A$ and $\mathcal{F}_B$; moreover, the operators a and b commute:

$$\begin{aligned}
&\left\{a_p^\dagger, a_q^\dagger\right\} = \left\{b_r^\dagger, b_s^\dagger\right\} = \{a_p, a_q\} = \{b_r, b_s\} = 0, \\
&\left\{a_p^\dagger, a_q\right\} = \delta_{pq}, \qquad \left\{b_r^\dagger, b_s\right\} = \delta_{rs}, \\
&\left[a_p^\dagger, b_r^\dagger\right] = \left[a_p^\dagger, b_s\right] = \left[a_q, b_r^\dagger\right] = [a_q, b_s] = 0.
\end{aligned}$$

Our aim is to apply the diagrammatic approach to calculate expectation values with respect to the tensor product of physical vacuum of spaces $\mathcal{F}_A$ and $\mathcal{F}_B$, i.e., $|0\rangle_A \otimes |0\rangle_B$. We separate operators acting on $\mathcal{F}_A$ and $\mathcal{F}_B$, and use Wick theorem for operators in both spaces independently. Consequently, the sign associated with a given full contraction is the product of the signs of the contractions within the A and B parts. In the diagrammatic interpretation, we only connect lines inside one of the monomers, i.e., we do not connect A and B lines. The signs of the A and B parts are obtained independently. Assigning the weight to a diagram is more challenging, but it can be shown that defining the weight as $w_R = (\text{rank}(\mathcal{G}))^{-1}$, where $\mathcal{G}$ is the group of automorphisms of the whole diagram, fulfills necessary conditions [see Eq. (1.6)]. We adapt a two-color convention for diagrams, where lines connected to blue vertices are fermion lines from $\mathcal{F}_A$ space, whereas lines connected to red vertices are fermion lines from $\mathcal{F}_B$. In some cases, especially for interaction operators, we will add a subscript A or B below a fermion line to indicate the space to which it belongs.

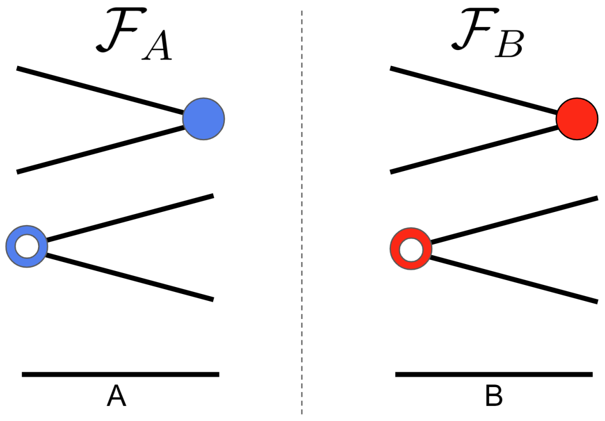

In next section, we will also use the notation:

$$\langle \hat{\Omega} \rangle = \langle \Phi_A \Phi_B | \, \hat{\Omega} \, | \Phi_A \Phi_B \rangle \, .$$

5 Diagrammatic representation of SAPT operators

Consider two types of operators which appear in first-order SAPT energy expresssion: the intermolecular interaction operator $\hat{V}$ and exchange operators $\hat{\mathcal{P}}_{2k}$ for $k = 1, 2, \ldots$ The interaction operator splits into four parts: the interaction between the nuclei of the monomers (V_{AB}), the interaction between nuclei of monomer A with electrons of monomer B ($\hat{V}_A$), interaction between nuclei of monomer B with electrons of monomer A ($\hat{V}_B$), and the interaction between electrons from A and B ($\hat{V}_{\mathrm{ee}}$):

$$\begin{aligned} \hat{V} &= V_{AB} + \hat{V}_A + \hat{V}_B + \hat{V}_{\mathrm{ee}} \\ &= V_{AB} + \sum_{rs \in B} v^A_{rs} b^\dagger_r b_s + \sum_{pq \in A} v^B_{pq} a^\dagger_p a_q + \sum_{\substack{pq \in A \\ rs \in B}} a^\dagger_p a_q b^\dagger_r b_s v^{qs}_{pr}. \end{aligned}$$

where $v^{qs}_{pr} = (pq|rs)$. We will assign indices p, q to monomer A and r, s to monomer B. Let us look at the diagrammatic representation of these operators (in the context of physical vacuum diagrams):

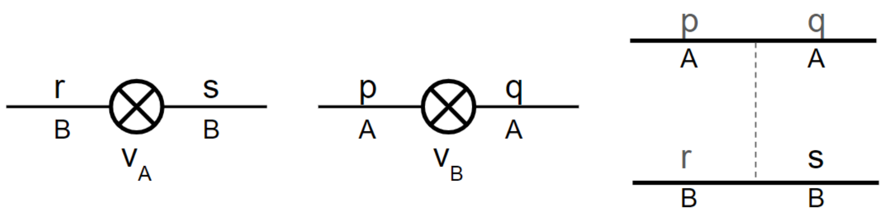

It is worth mentioning that the lines in $\hat{V}_{\mathrm{ee}}$ are not equivalent, because they do not belong to the same Fock space, therefore there is no $1/2$ weight in the operator expression (this weight is present, for example, in the intramolecular electron-electron interaction).

Let us discuss exchange operators starting with $\hat{\mathcal{P}}_2$:

$$\hat{\mathcal{P}}_2 = (-1) \sum_{pq \in A} \sum_{rs \in B} S_{ps} S_{rq} a_p^\dagger a_q b_r^\dagger b_s.$$

Defining $P_{pq,rs} = S_{ps} S_{rq}$ gives

$$\hat{\mathcal{P}}_2 = (-1) \sum_{pq \in A} \sum_{rs \in B} P_{pq,rs} a_p^\dagger a_q b_r^\dagger b_s.$$

We can now connect the $\hat{\mathcal{P}}_2$ operator with a standard two-body diagram:

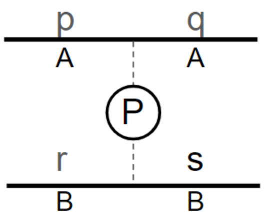

A drawback of this diagram is that it does not account for the correct sign of the operator. Instead, consider the following representation of the $\hat{\mathcal{P}}_2$ operator:

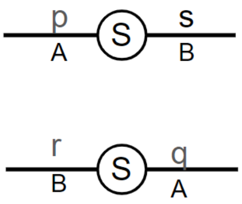

This representation of $\hat{\mathcal{P}}_2$ has several advantages:

- It is easy to connect that diagram with the scalar factor $S_{ps} S_{rq}$.
- Switching from P-representation to S-representation of the operator, we effectively change the number of loops in the whole diagram by one, which results in the (-1) factor. This factor is present in the definition of $\hat{\mathcal{P}}_2$.

This approach can be generalized to $\hat{\mathcal{P}}_{2k}$ diagrams. Operator $\hat{\mathcal{P}}_{2k}$ is defined as:

$$\hat{\mathcal{P}}_{2k} = (-1)^k \left(\frac{1}{k!}\right)^2 \sum_{\substack{p_1,p_2,\ldots,p_k \in A \\ q_1,q_2,\ldots,q_k \in A}} \sum_{\substack{r_1,r_2,\ldots,r_k \in B \\ s_1,s_2,\ldots,s_k \in B}} S^{s_1}_{p_1} S^{s_2}_{p_2} \ldots S^{s_k}_{p_k} S^{q_1}_{r_1} S^{q_2}_{r_2} S^{q_k}_{r_k} a^{\dagger}_{p_1} \ldots a^{\dagger}_{p_k} a_{q_k} \ldots a_{q_1} b^{\dagger}_{r_1} \ldots b^{\dagger}_{r_k} b_{s_k} \ldots b_{s_1},$$

which can be diagrammatically represented as:

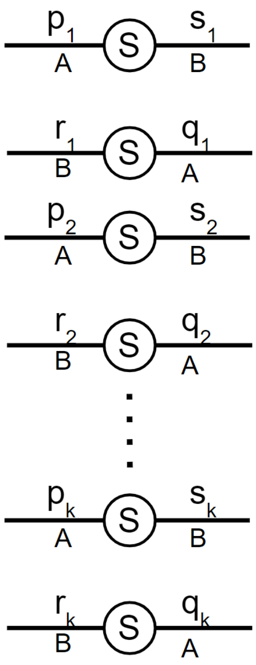

The $(-1)^k$ sign factor comes from changing the number of loops relative to the standard representation of this operator by one exactly k times. The $\left(\frac{1}{k!}\right)^2$ factor arises from the number of equivalent lines in the skeleton: we can permute independently k occurrences of the $A-S-B$ line and k occurrences of the $B-S-A$ line. Moreover, drawing all vertices above each other emphasizes that there is no possibility of contraction inside this operator, i.e., one cannot connect the fermion lines inside this s-vertex.

With the above definitions of interaction and exchange operators and their diagrammatic representations, we can now evaluate the operators appearing in the first-order SAPT energy.

First, consider the operator $\hat{V}_B\hat{\mathcal{P}}_2$:

$$\hat{V}_B\hat{\mathcal{P}}_2 = -\sum_{\substack{p_1q_1p_2q_2\in A\\ rs\in B}} v^B_{p_1q_1} S_{p_2s}S_{rq_2} a^\dagger_{p_1}a_{q_1}a^\dagger_{p_2}a_{q_2}b^\dagger_r b_s$$

$$= +\sum_{\substack{p_1q_1p_2q_2\in A\\ rs\in B}} v^B_{p_1q_1} S_{p_2s}S_{rq_2} a^\dagger_{p_1}a^\dagger_{p_2}a_{q_1}a_{q_2}b^\dagger_r b_s - \sum_{\substack{p_1q_1p_2q_2\in A\\ rs\in B}} v^B_{p_1q_1} S_{p_2s}S_{rq_2} a^\dagger_{p_1}\delta_{q_1p_2}a_{q_2}b^\dagger_r b_s$$

$$= -\sum_{\substack{p_1q_1p_2q_2\in A\\ rs\in B}} v^B_{p_1q_1} S_{p_2s}S_{rq_2} a^\dagger_{p_1}a^\dagger_{p_2}a_{q_2}a_{q_1}b^\dagger_r b_s - \sum_{\substack{p_1p_2q_2\in A\\ rs\in B}} v^B_{p_1p_2} S_{p_2s}S_{rq_2} a^\dagger_{p_1}a_{q_2}b^\dagger_r b_s.$$

In the diagrammatic representation we obtain:

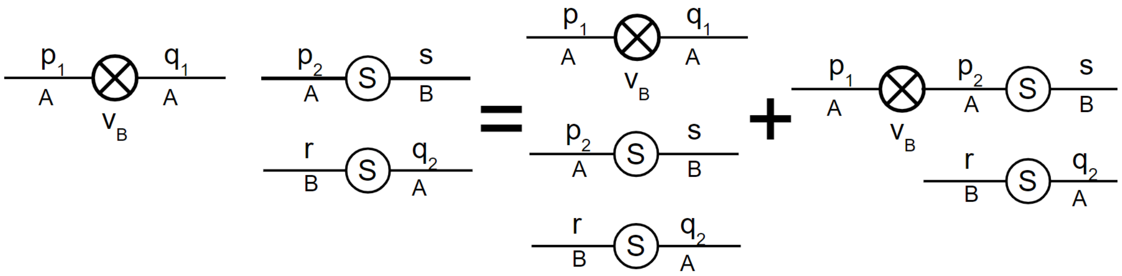

From the resolution of identity we get that $\sum_{p_2} v^B_{p_1p_2}S_{p_2s} = v^B_{p_1s}$. Thus, we can rewrite the above equation as:

$$\hat{V}_B\hat{\mathcal{P}}_2 = -\sum_{\substack{p_1q_1p_2q_2\in A\\ rs\in B}} v^B_{p_1q_1} S_{p_2s}S_{rq_2} a^\dagger_{p_1}a^\dagger_{p_2}a_{q_2}a_{q_1}b^\dagger_r b_s - \sum_{\substack{p_1q_1\in A\\ rs\in B}} v^B_{p_1s} S_{rq_1} a^\dagger_{p_1}a_{q_1}b^\dagger_r b_s,$$

and redraw the last diagram:

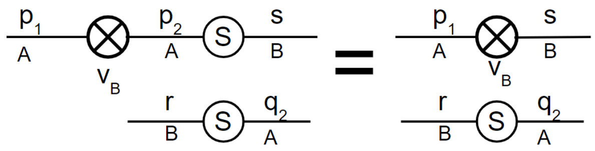

An analogous diagram follows for $\hat{V}_A\hat{\mathcal{P}}_2$:

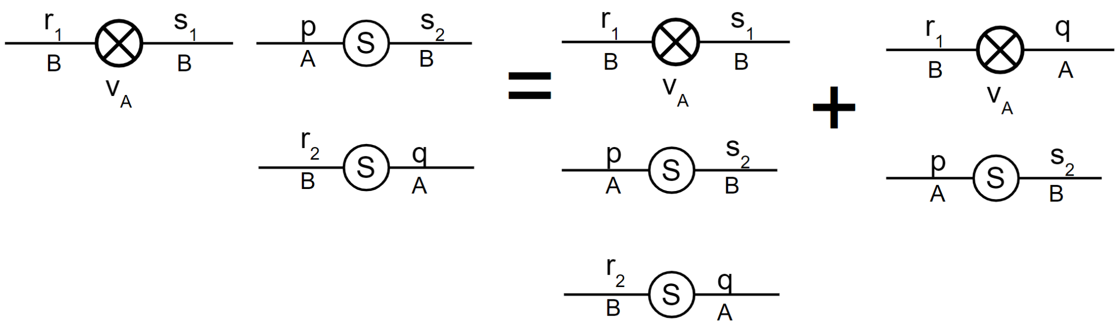

The $\hat{V}_{\mathrm{ee}}\hat{\mathcal{P}}_2$ operator can be rewritten in the same way:

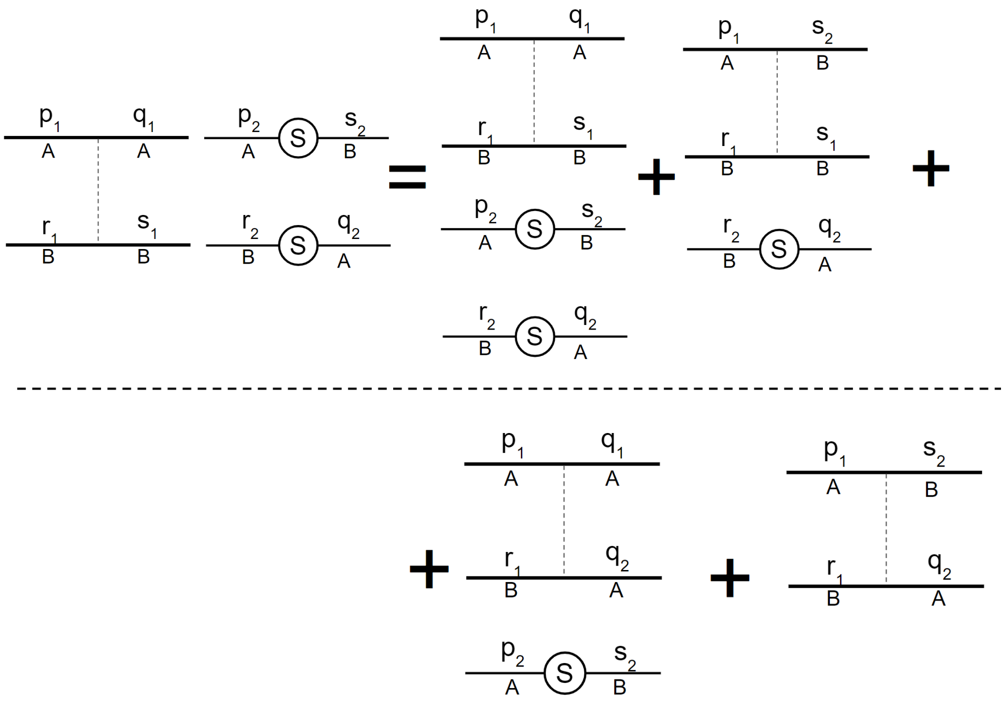

Similarly, we can draw diagrams for the $\hat{V}\hat{\mathcal{P}}_{2k}$ operator.

We can now evaluate diagrammatically the $\langle \hat{V}\hat{\mathcal{P}}_{2k}\rangle$ expectation value. In the resulting diagrams, we can only connect A fermion lines to blue vertices, whereas B fermion lines are connected to red vertices. Naturally, blue and red vertices can only be linked through the $\hat{\mathcal{P}}_{2k}$ or $\hat{V}\hat{\mathcal{P}}_{2k}$ operator. Observing the examples presented above, we see that inside these operators the lines change their character from A to B or vice versa exactly $2k$ times. In consequence, in the resulting diagram of $\langle \hat{V}\hat{\mathcal{P}}_{2k}\rangle$ the color also changes exactly $2k$ times.

Finally, we omit drawing the S vertices, as a direct connection between a red and a blue vertex can only occur through an S vertex. Now, we are ready to summarize the rules for obtaining the first-order exchange energy using the diagrammatic method.

6 Rules for evaluating first-order SAPT exchange diagrams

We split this section in two parts. First, we summarize the rules for obtaining all possible bare skeletons containing the $\hat{\mathcal{P}}_{2k}$ operator:

1. We draw all possible non-equivalent skeletons for $\langle \hat{V}_A \hat{\mathcal{P}}_{2k} \rangle$, $\langle \hat{V}_B \hat{\mathcal{P}}_{2k} \rangle$, $\langle \hat{V}_{\text{ee}} \hat{\mathcal{P}}_{2k} \rangle$, and $\langle \hat{\mathcal{P}}_{2k} \rangle$ separately.
2. Each skeleton contains an equal number of closed blue vertices and open blue vertices. The same goes for red vertices.
3. There can be up to two vertices of the same color directly connected to each other.
4. Each open vertex is placed either between two closed vertices, between a closed vertex and an interaction vertex, or between two interaction vertices.
5. Each closed vertex is placed either between two open vertices, between an open vertex and an interaction vertex, or between two interaction vertices.
6. Each interaction vertex connects an open and a closed vertex, where the V_A vertex is always connected to an open red vertex, the V_B vertex is always connected to an open blue vertex, and the V_{ee} vertex is always connected to one open blue vertex and one open red vertex.
7. The total number of color changes inside all loops in a skeleton is equal to $2k$.

8. The number of color changes from a red open vertex to a blue closed vertex must be equal to the number of color changes from a blue open vertex to a red closed vertex, and equal to k to assure a proper form of the $\mathcal{P}_{2k}$ skeleton.

After having drawn all possible non-equivalent skeletons fulfilling these rules, we can assign them scalar quantities. To do that:

1. Find the group of automorphisms $\mathcal{G}$ of the skeleton and assign the skeleton weigh as $w_R = (\text{rank}(\mathcal{G}))^{-1}$. The symmetries of a skeleton can originate from equivalence of lines, equivalence of vertices, or even equivalence of whole loops.

2. Label the vertices, keeping in mind that that each geminal index has to be present twice: once on a closed vertex, and once on an open vertex. Additionally, two connected vertices of the same color must have the same geminal index.

3. Label the fermion lines, remembering that lines connected to a single geminal vertex share the same orbital index. In this way, the lines connecting red and blue vertices have two different indices, but it is important to keep in mind that they are actually connected through the S vertex.

4. Assign a scalar d to the diagram using vertex scalars and S matrix elements, which appear for each direct connection between a red and a blue vertex.

5. To calculate the sign factor of the whole diagram, count the number of open vertices (i.e., the $\tilde{G}$ vertices) n and the number of loops l.

6. The final scalar quantity obtained from such skeleton is equal to $(-1)^{n+l}\, 2^l\, w_R\, d$. The 2^l factor comes from summation over all possible spins.

7. Sum over all possible geminal indices and orbital indices within a given geminal. In summing over geminal indices, keep in mind that each geminal appears only once in the wave function.

7 Examples

7.1 Example 1: $\langle \hat{\mathcal{P}}_2 \rangle$

The only possible skeleton containing $\mathcal{P}_2$ can be drawn as follows:

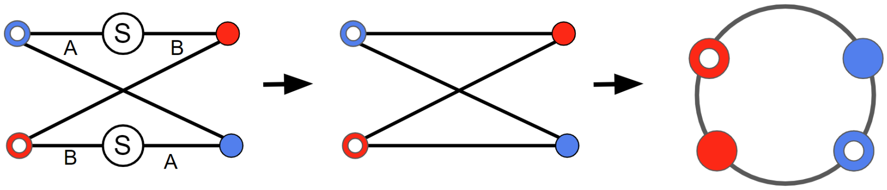

As we can see, any connection between a blue and a red vertex must pass through the S vertex. Thus, there is no need to explicitly draw this vertex. On the right, the skeleton is represented as a cyclic skeleton. Now, let us add labels for the vertices and fermion lines:

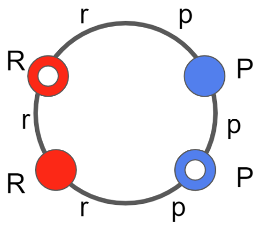

From the diagram, we immediately recognize that there are only two geminals R and P, because if two vertices of the same color are directly connected, then they have to belong to the same geminal. Also, there are only two orbital indices p and r. This comes from fact that in GVB-PP in the natural orbitals representation, both lines outgoing from a geminal vertex have the same orbital index. Moreover, observe that two lines in the diagram are assigned two different indices. These lines account for the S matrix elements in the final expression. Now, count the number of $\tilde{G}$ vertices: $n = 2$. The number of loops is $l = 1$. The weight of that diagram is $w_R = 1$. The scalar d connected to the diagram is:

$$d = (c_r)^2 (c_p)^2 (S_{pr})^2,$$

where $r \in I_R$, $p \in I_P$. Using the occupation numbers, $n_p = (c_p)^2, n_r = (c_r)^2$, we arrive at the final expression:

$$\langle \hat{\mathcal{P}}_2 \rangle = \sum_{\substack{I_P \in G(A) \\ I_R \in G(B)}} \sum_{\substack{p \in I_P \\ r \in I_R}} -2 n_r n_p (S_{pr})^2 .$$

The notation $I_P \in G(A), I_R \in G(B)$ indicates that P geminal is present in the $|\Phi_A\rangle$ wave function, whereas R geminal is present in the $|\Phi_B\rangle$ wave function.

7.2 Example 2: An example diagram in $\langle \hat{V}_{\mathrm{ee}} \hat{\mathcal{P}}_2 \rangle$

The next example we consider is one of the possible skeletons in $\langle \hat{V}_{\mathrm{ee}} \hat{\mathcal{P}}_2 \rangle$:

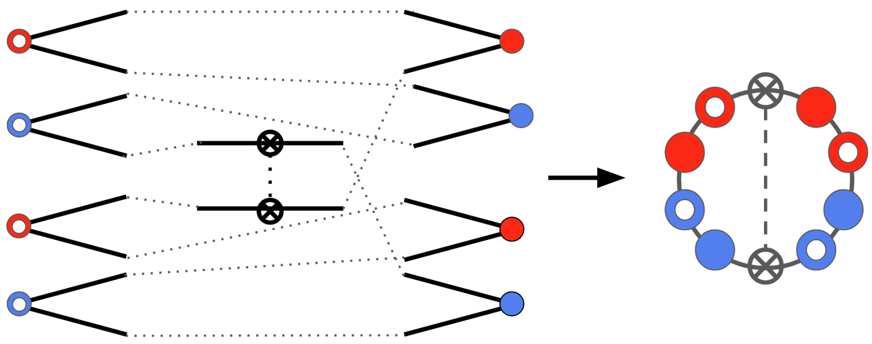

As in the previous example, we omit the S vertices, knowing that they are present wherever red and blue vertices are directly connected. Thus, we can say that this skeleton comes from $\langle \hat{V}_{\mathrm{ee}} \hat{\mathcal{P}}_2 \rangle$. Now, we draw the corresponding diagram:

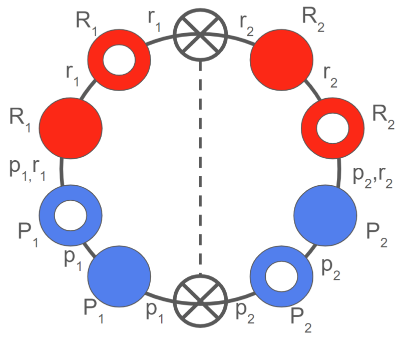

We assign the weight of that diagram: $w_R = 1$. The number of $\tilde{G}$ vertices is $n = 4$, and the number of loops is equal to $l = 1$. The scalar quantity is equal to:

$$d = (c_{p_1})^2 (c_{p_2})^2 (c_{r_1})^2 (c_{r_2})^2 S_{p_1 r_1} S_{p_2 r_2} v^{p_1 r_1}_{p_2 r_2},$$

where $p_1 \in I_{P_1}, p_2 \in I_{P_2}, r_1 \in I_{r_1}, r_2 \in I_{r_2}$. Therefore, the final contribution to the expectation value associated to that diagram reads:

$$\sum_{\substack{I_{P_1} \neq I_{P_2} \in G(A) \\ I_{R_1} \neq I_{R_2} \in G(B)}} \sum_{\substack{p_1 \in I_{P_1} p_2 \in I_{P_2} \\ r_1 \in I_{R_1} r_2 \in I_{R_2}}} -2 n_{p_1} n_{r_1} n_{r_2} n_{p_2} S_{p_1 r_1} S_{r_2 p_2} v^{p_1 r_1}_{p_2 r_2}.$$

Notice the limitation in the geminal summation, which arises from the fact that each geminal in the wave function is present only once.

7.3 Example 3: $\langle \hat{V}_A \hat{\mathcal{P}}_2 \rangle$

The next example are all distinguishable skeletons in $\langle \hat{V}_A \hat{\mathcal{P}}_2 \rangle$.

- One loop skeletons:

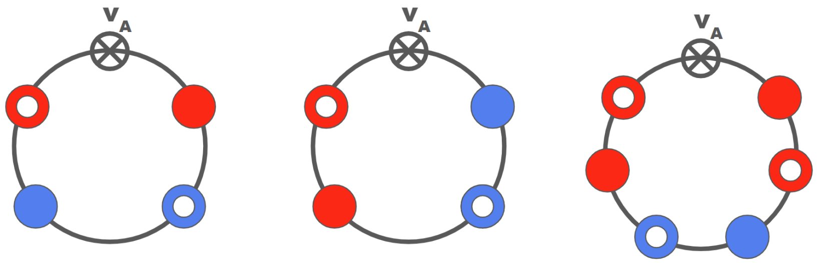

- Two loops skeleton:

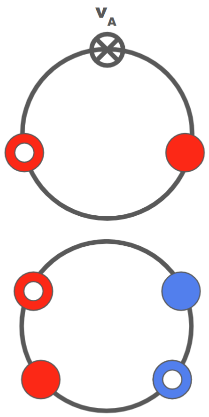

7.4 Examples of equivalency in diagrams

In skeletons containing the $\hat{\mathcal{P}}_4$ operator, it is important to assign the proper weight factor due to a higher symmetry of the skeleton. As the first example, consider one of the $\langle\hat{\mathcal{P}}_4\rangle$ skeletons:

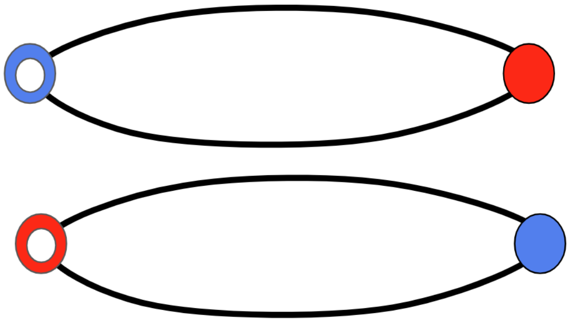

In this skeleton, we have two pairs of equivalent lines, and the weight factor is $w_R = \left(\frac{1}{2}\right)^2$.

Another example of higher symmetry, which is also present in $\langle\hat{\mathcal{P}}_4\rangle$, appears in the following skeleton:

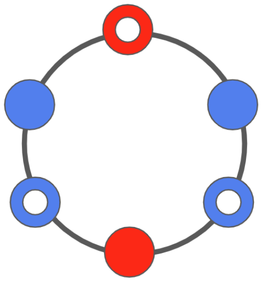

This is an example of equivalent vertices, which results in the weight factor $w_R = \frac{1}{2}$.

The last possibility of equivalency in $\langle \hat{\mathcal{P}}_4 \rangle$ is the equivalence of loops, which is present in the skeleton:

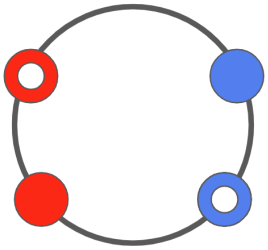

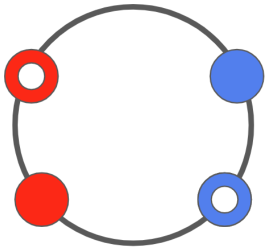

This equivalence results in weight factor $w_R = \frac{1}{2}$.

References

(S1) Korona, T.; Moszynski, R.; Jeziorski, B. Convergence of Symmetry-Adapted Perturbation Theory for the Interaction between Helium Atoms and between a Hydrogen Molecule and a Helium Atom. *Adv. Quantum Chem.* **1997**, *28*, 171–188.

(S2) Patkowski, K.; Szalewicz, K. Frozen core and effective core potentials in symmetry-adapted perturbation theory. *J. Chem. Phys.* **2007**, *127*, 164103.

(S3) Smith, D. G. A.; Burns, L. A.; Patkowski, K.; Sherrill, C. D. Revised Damping Parameters for the D3 Dispersion Correction to Density Functional Theory. *J. Phys. Chem. Lett.* **2016**, *7*, 2197–2203.

(S4) Moszynski, R.; Jeziorski, B.; Szalewicz, K. Many-body theory of exchange effects in intermolecular interactions. Second-quantization approach and comparison with full configuration interaction results. *J. Chem. Phys.* **1994**, *100*, 1312–1325.

(S5) Paldus, J. *Diagrammatical methods for many-fermion systems*; Radboud University Nijmegen, 1981; Chapter 5.

(S6) Paldus, J. Diagrammatical Method for Geminals. I. Theory. *J. Chem. Phys.* **1972**, *57*, 638–651.

(S7) Paldus, J.; Sengupta, S.; Čížek, J. Diagrammatical Method for Geminals. II. Applications. *J. Chem. Phys.* **1972**, *57*, 652–666.

(S8) Kutzelnigg, W. Direct Determination of Natural Orbitals and Natural Expansion Coefficients of Many-Electron Wavefunctions. I. Natural Orbitals in the Geminal Product Approximation. *J. Chem. Phys.* **1964**, *40*, 3640–3647.

(S9) Surján, P. R. In *Correlation and Localization*; Surján, P. R., Bartlett, R. J., Bogár, F., Cooper, D. L., Kirtman, B., Klopper, W., Kutzelnigg, W., March, N. H., Mezey, P. G., Müller, H., Noga, J., Paldus, J., Pipek, J., Raimondi, M., Røeggen, I., Sun, J. Q.,

Surján, P. R., Valdemoro, C., Vogtner, S., Eds.; Springer: Berlin, Heidelberg, 1999; pp 63–88.

(S10) Hurley, A. C.; Lennard-Jones, J. E.; Pople, J. A. The molecular orbital theory of chemical valency XVI. A theory of paired-electrons in polyatomic molecules. *Proc. R. Soc. London Ser. A* **1953**, *220*, 446–455.

(S11) Arai, T. Theorem on Separability of Electron Pairs. *J. Chem. Phys.* **1960**, *33*, 95–98.

Working expressions and diagrams

I. FINAL EXPRESSIONS

First-order electrostatic energy $E^{(1)}_{\text{elst}}$ is calculated as:

$$E^{(1)}_{\text{elst}} = V_{AB} + \sum_{I_P \in G(A)} \sum_{p \in I_P} 2 n_p v^B_{pp} + \sum_{I_R \in G(B)} \sum_{r \in I_R} 2 n_r v^A_{rr} + \sum_{\substack{I_P \in G(A) \\ I_R \in G(B)}} \sum_{\substack{p \in I_P \\ r \in I_R}} 4 n_p n_r v^{pr}_{pr}$$

First-order exchange energy proportional to S^2 is equal to:

$$E^{(1)}_{\text{exch}}(\propto S^2) = \langle \hat{V} \mathcal{P}_2 \rangle - E^{(1)}_{\text{elst}} \langle \mathcal{P}_2 \rangle$$

which can be rewritten as:

$$E^{(1)}_{\text{exch}}(\propto S^2) = \langle (\hat{V} - V_{AB}) \mathcal{P}_2 \rangle - (E^{(1)}_{\text{elst}} - V_{AB}) \langle \mathcal{P}_2 \rangle$$

We split $\langle (\hat{V} - V_{AB}) \mathcal{P}_2 \rangle$ into parts:

$$\langle (\hat{V} - V_{AB}) \mathcal{P}_2 \rangle = \langle \hat{V}_A \mathcal{P}_2 \rangle + \langle \hat{V}_B \mathcal{P}_2 \rangle + \langle \hat{V}_{ee} \mathcal{P}_2 \rangle$$

and split all parts according to the number of loops in a diagram:

$$\langle \hat{V}_A \mathcal{P}_2 \rangle = \langle \hat{V}_A \mathcal{P}_2 \rangle_{\text{1-loop}} + \langle \hat{V}_A \mathcal{P}_2 \rangle_{\text{2-loops}}$$

$$\langle \hat{V}_B \mathcal{P}_2 \rangle = \langle \hat{V}_B \mathcal{P}_2 \rangle_{\text{1-loop}} + \langle \hat{V}_B \mathcal{P}_2 \rangle_{\text{2-loops}}$$

$$\langle \hat{V}_{ee} \mathcal{P}_2 \rangle = \langle \hat{V}_{ee} \mathcal{P}_2 \rangle_{\text{1-loop}} + \langle \hat{V}_{ee} \mathcal{P}_2 \rangle_{\text{2-loops}} + \langle \hat{V}_{ee} \mathcal{P}_2 \rangle_{\text{3-loops}}$$

We also calculate $\langle \mathcal{P}_2 \rangle$ as:

$$\langle \mathcal{P}_2 \rangle = \langle \mathcal{P}_2 \rangle_{\text{1-loop}}$$

In this work, we also calculate $E^{(1)}_{\text{exch}}(\propto S^4)$:

$$E^{(1)}_{\text{exch}}(\propto S^4) = \langle \hat{V} \mathcal{P}_4 \rangle - E^{(1)}_{\text{exch}}(\propto S^2) \langle \mathcal{P}_2 \rangle - E^{(1)}_{\text{elst}} \langle \mathcal{P}_4 \rangle$$

This equation can be rewritten as:

$$E^{(1)}_{\text{exch}}(\propto S^4) = \langle (\hat{V} - V_{AB}) \mathcal{P}_4 \rangle - E^{(1)}_{\text{exch}}(\propto S^2) \langle \mathcal{P}_2 \rangle - (E^{(1)}_{\text{elst}} - V_{AB}) \langle \mathcal{P}_4 \rangle$$

In the same spirit as before, we divide $\langle (\hat{V} - V_{AB}) \mathcal{P}_4 \rangle$ into parts:

$$\langle (\hat{V} - V_{AB}) \mathcal{P}_4 \rangle = \langle \hat{V}_A \mathcal{P}_4 \rangle + \langle \hat{V}_B \mathcal{P}_4 \rangle + \langle \hat{V}_{ee} \mathcal{P}_4 \rangle$$

and we split these parts according to the number of loops in a diagram:

$$\langle \hat{V}_A \mathcal{P}_4 \rangle = \langle \hat{V}_A \mathcal{P}_4 \rangle_{\text{1-loop}} + \langle \hat{V}_A \mathcal{P}_4 \rangle_{\text{2-loops}} + \langle \hat{V}_A \mathcal{P}_4 \rangle_{\text{3-loops}}$$

$$\langle \hat{V}_B \mathcal{P}_4 \rangle = \langle \hat{V}_B \mathcal{P}_4 \rangle_{\text{1-loop}} + \langle \hat{V}_B \mathcal{P}_4 \rangle_{\text{2-loops}} + \langle \hat{V}_B \mathcal{P}_4 \rangle_{\text{3-loops}}$$

$$\langle \hat{V}_{ee} \mathcal{P}_4 \rangle = \langle \hat{V}_{ee} \mathcal{P}_4 \rangle_{\text{1-loop}} + \langle \hat{V}_{ee} \mathcal{P}_4 \rangle^a_{\text{2-loops}} + \langle \hat{V}_{ee} \mathcal{P}_4 \rangle^b_{\text{2-loops}} + \langle \hat{V}_{ee} \mathcal{P}_4 \rangle_{\text{3-loops}} + \langle \hat{V}_{ee} \mathcal{P}_4 \rangle_{\text{4-loops}}$$

Here $\langle \hat{V}_{ee} \mathcal{P}_4 \rangle^a_{\text{2-loops}}$ represents all diagrams built from two loops, both containing interaction vertices. The $\langle \hat{V}_{ee} \mathcal{P}_4 \rangle^b_{\text{2-loops}}$ term represents all two-loops diagrams, where only one loop contains interaction vertices. Additionally, $\langle \mathcal{P}_4 \rangle$ is calculated as:

$$\langle \mathcal{P}_4 \rangle = \langle \mathcal{P}_4 \rangle_{\text{1-loop}} + \langle \mathcal{P}_4 \rangle_{\text{2-loops}}$$

Colors in equations below represent the weight factor coming either from the equivalency of lines, vertices, or loops.

II. $E^{(1)}_{\mathbf{exch}}(\propto S^2)$

A. $\langle \hat{V}_{ee}\mathcal{P}_2\rangle$

$$\langle \hat{V}_{ee}\mathcal{P}_2\rangle_{\text{1-loop}} = \sum_{\substack{I_{P_1}\in G(A)\\ I_{R_1}\in G(B)}} \sum_{\substack{p_1\in I_{P_1}\\ r_1\in I_{R_1}}} -2n_{p_1}n_{r_1}v^{p_1p_1}_{r_1r_1} \tag{1}$$

$$+ \sum_{\substack{I_{P_1}\in G(A)\\ I_{R_1}\in G(B)}} \sum_{\substack{p_1q_1\in I_{P_1}\\ r_1s_1\in I_{R_1}}} -2c_{p_1}c_{r_1}c_{s_1}c_{q_1}S_{p_1r_1}S_{s_1q_1}v^{p_1r_1}_{q_1s_1} \tag{2}$$

$$+ \sum_{\substack{I_{P_1}\neq I_{P_2}\in G(A)\\ I_{R_1}\in G(B)}} \sum_{\substack{p_1\in I_{P_1}p_2\in I_{P_2}\\ r_1\in I_{R_1}}} 2n_{p_1}n_{r_1}n_{p_2}S_{r_1p_2}v^{p_1p_1}_{p_2r_1} \tag{3}$$

$$+ \sum_{\substack{I_{P_1}\in G(A)\\ I_{R_1}\in G(B)}} \sum_{\substack{p_1\in I_{P_1}\\ r_1s_1\in I_{R_1}}} -2n_{p_1}c_{r_1}c_{s_1}S_{p_1r_1}v^{p_1r_1}_{s_1s_1} \tag{4}$$

$$+ \sum_{\substack{I_{P_1}\in G(A)\\ I_{R_1}\in G(B)}} \sum_{\substack{p_1q_1\in I_{P_1}\\ r_1\in I_{R_1}}} -2c_{p_1}n_{r_1}c_{q_1}S_{p_1r_1}v^{p_1r_1}_{q_1q_1} \tag{5}$$

$$+ \sum_{\substack{I_{P_1}\neq I_{P_2}\in G(A)\\ I_{R_1}\in G(B)}} \sum_{\substack{p_1\in I_{P_1}p_2\in I_{P_2}\\ r_1s_1\in I_{R_1}}} 2n_{p_1}c_{r_1}c_{s_1}n_{p_2}S_{p_1r_1}S_{s_1p_2}v^{p_1r_1}_{p_2s_1} \tag{6}$$

$$+ \sum_{\substack{I_{P_1}\in G(A)\\ I_{R_1}\neq I_{R_2}\in G(B)}} \sum_{\substack{p_1q_1\in I_{P_1}\\ r_1\in I_{R_1}r_2\in I_{R_2}}} 2c_{p_1}n_{r_1}n_{r_2}c_{q_1}S_{p_1r_1}S_{r_2q_1}v^{p_1r_1}_{q_1r_2} \tag{7}$$

$$+ \sum_{\substack{I_{P_1}\in G(A)\\ I_{R_1}\neq I_{R_2}\in G(B)}} \sum_{\substack{p_1\in I_{P_1}\\ r_1\in I_{R_1}r_2\in I_{R_2}}} 2n_{p_1}n_{r_1}n_{r_2}S_{p_1r_1}v^{p_1r_1}_{r_2r_2} \tag{8}$$

$$+ \sum_{\substack{I_{P_1}\neq I_{P_2}\in G(A)\\ I_{R_1}\neq I_{R_2}\in G(B)}} \sum_{\substack{p_1\in I_{P_1}p_2\in I_{P_2}\\ r_1\in I_{R_1}r_2\in I_{R_2}}} -2n_{p_1}n_{r_1}n_{r_2}n_{p_2}S_{p_1r_1}S_{r_2p_2}v^{p_1r_1}_{p_2r_2} \tag{9}$$

$$\langle \hat{V}_{ee}\mathcal{P}_2\rangle_{\text{2-loops}} = \sum_{\substack{I_{P_1}\neq I_{P_2}\in G(A)\\ I_{R_1}\in G(B)}} \sum_{\substack{p_1\in I_{P_1} p_2\in I_{P_2}\\ r_1 s_1\in I_{R_1}}} -4n_{p_1}c_{r_1}n_{p_2}c_{s_1}S_{r_1p_2}S_{p_2s_1}v^{p_1r_1}_{p_1s_1} \tag{10}$$

$$+ \sum_{\substack{I_{P_1}\neq I_{P_2}\in G(A)\\ I_{R_1}\in G(B)}} \sum_{\substack{p_1\in I_{P_1} p_2\in I_{P_2}\\ r_1\in I_{R_1}}} -4n_{p_1}n_{r_1}n_{p_2}S_{r_1p_2}v^{p_1r_1}_{p_1p_2} \tag{11}$$

$$+ \sum_{\substack{I_{P_1}\neq I_{P_2}\in G(A)\\ I_{R_1}\neq I_{R_2}\in G(B)}} \sum_{\substack{p_1\in I_{P_1} p_2\in I_{P_2}\\ r_1\in I_{R_1} r_2\in I_{R_2}}} 4n_{p_1}n_{r_1}n_{p_2}n_{r_2}S_{r_1p_2}S_{p_2r_2}v^{p_1r_1}_{p_1r_2} \tag{12}$$

$$+ \sum_{\substack{I_{P_1}\in G(A)\\ I_{R_1}\neq I_{R_2}\in G(B)}} \sum_{\substack{p_1\in I_{P_1}\\ r_1\in I_{R_1} r_2\in I_{R_2}}} -4n_{p_1}n_{r_1}n_{r_2}S_{p_1r_1}v^{p_1r_2}_{r_1r_2} \tag{13}$$

$$+ \sum_{\substack{I_{P_1}\in G(A)\\ I_{R_1}\neq I_{R_2}\in G(B)}} \sum_{\substack{p_1 q_1\in I_{P_1}\\ r_1\in I_{R_1} r_2\in I_{R_2}}} -4c_{p_1}n_{r_1}c_{q_1}n_{r_2}S_{p_1r_1}S_{r_1q_1}v^{p_1r_2}_{q_1r_2} \tag{14}$$

$$+ \sum_{\substack{I_{P_1}\neq I_{P_2}\in G(A)\\ I_{R_1}\neq I_{R_2}\in G(B)}} \sum_{\substack{p_1\in I_{P_1} p_2\in I_{P_2}\\ r_1\in I_{R_1} r_2\in I_{R_2}}} 4n_{p_1}n_{r_1}n_{p_2}n_{r_2}S_{p_1r_1}S_{r_1p_2}v^{p_1r_2}_{p_2r_2} \tag{15}$$

$$\langle \hat{V}_{ee}\mathcal{P}_2\rangle_{\text{3-loops}} = \sum_{\substack{I_{P_1}\neq I_{P_2}\in G(A)\\ I_{R_1}\neq I_{R_2}\in G(B)}} \sum_{\substack{p_1\in I_{P_1} p_2\in I_{P_2}\\ r_1\in I_{R_1} r_2\in I_{R_2}}} -8n_{r_1}n_{p_1}S_{p_1r_1}S_{p_1r_1}n_{p_2}n_{r_2}v^{p_2r_2}_{p_2r_2} \tag{16}$$

B. $\langle \hat{V}_A \mathcal{P}_2 \rangle$

$$\langle \hat{V}_A \mathcal{P}_2 \rangle_{\text{1-loop}} = \sum_{\substack{I_{P_1} \in G(A) \\ I_{R_1} \in G(B)}} \sum_{\substack{p_1 \in I_{P_1} \\ r_1 s_1 \in I_{R_1}}} -2 c_{r_1} n_{p_1} c_{s_1} S_{r_1 p_1} S_{p_1 s_1} (v_A)_{r_1 s_1} \quad (17)$$

$$+ \sum_{\substack{I_{P_1} \in G(A) \\ I_{R_1} \in G(B)}} \sum_{\substack{p_1 \in I_{P_1} \\ r_1 \in I_{R_1}}} -2 n_{r_1} n_{p_1} S_{r_1 p_1} (v_A)_{r_1 p_1} \quad (18)$$

$$+ \sum_{\substack{I_{P_1} \in G(A) \\ I_{R_1} \neq I_{R_2} \in G(B)}} \sum_{\substack{p_1 \in I_{P_1} \\ r_1 \in I_{R_1} r_2 \in I_{R_2}}} 2 n_{r_1} n_{p_1} n_{r_2} S_{r_1 p_1} S_{p_1 r_2} (v_A)_{r_1 r_2} \quad (19)$$

$$\langle \hat{V}_A \mathcal{P}_2 \rangle_{\text{2-loops}} = \sum_{\substack{I_{P_1} \in G(A) \\ I_{R_1} \neq I_{R_2} \in G(B)}} \sum_{\substack{p_1 \in I_{P_1} \\ r_1 \in I_{R_1} r_2 \in I_{R_2}}} - 4 n_{r_1} n_{p_1} S_{p_1 r_1} S_{p_1 r_1} n_{r_2} (v_A)_{r_2 r_2} \quad (20)$$

C. $\langle \hat{V}_B \mathcal{P}_2 \rangle$

$$\langle \hat{V}_B \mathcal{P}_2 \rangle_{\text{1-loop}} = \sum_{\substack{I_{P_1} \in G(A) \\ I_{R_1} \in G(B)}} \sum_{\substack{p_1 \in I_{P_1} \\ r_1 \in I_{R_1}}} -2 n_{p_1} n_{r_1} S_{p_1 r_1} (v_B)_{p_1 r_1} \quad (21)$$

$$+ \sum_{\substack{I_{P_1} \in G(A) \\ I_{R_1} \in G(B)}} \sum_{\substack{p_1 q_1 \in I_{P_1} \\ r_1 \in I_{R_1}}} -2 c_{p_1} n_{r_1} c_{q_1} S_{p_1 r_1} S_{r_1 q_1} (v_B)_{p_1 q_1} \quad (22)$$

$$+ \sum_{\substack{I_{P_1} \neq I_{P_2} \in G(A) \\ I_{R_1} \in G(B)}} \sum_{\substack{p_1 \in I_{P_1} p_2 \in I_{P_2} \\ r_1 \in I_{R_1}}} 2 n_{p_1} n_{r_1} n_{p_2} S_{p_1 r_1} S_{r_1 p_2} (v_B)_{p_1 p_2} \quad (23)$$

$$\langle \hat{V}_B \mathcal{P}_2 \rangle_{\text{2-loops}} = \sum_{\substack{I_{P_1} \neq I_{P_2} \in G(A) \\ I_{R_1} \in G(B)}} \sum_{\substack{p_1 \in I_{P_1} p_2 \in I_{P_2} \\ r_1 \in I_{R_1}}} - 4 n_{r_1} n_{p_1} S_{p_1 r_1} S_{p_1 r_1} n_{p_2} (v_B)_{p_2 p_2} \quad (24)$$

D. $\langle \mathcal{P}_2 \rangle$

$$\langle \mathcal{P}_2 \rangle_{\text{1-loop}} = \sum_{\substack{I_{P_1} \in G(A) \\ I_{R_1} \in G(B)}} \sum_{p_1 \in I_{P_1} r_1 \in I_{R_1}} -2 n_{r_1} n_{p_1} S_{p_1 r_1} S_{p_1 r_1} \quad (25)$$

III. $E^{(1)}_{\mathbf{exch}}(\propto S^4)$

A. $\langle \hat{V}_{ee}\mathcal{P}_4 \rangle$

$$\langle \hat{V}_{ee}\mathcal{P}_4 \rangle_{\text{1-loop}} =$$

$$\sum_{\substack{I_{P_1}\neq I_{P_2}\in G(A)\\ I_{R_1}\in G(B)}} \sum_{\substack{p_1\in I_{P_1} p_2\in I_{P_2}\\ r_1 s_1\in I_{R_1}}} 2n_{p_1}c_{r_1}n_{p_2}c_{s_1}S_{r_1p_2}S_{p_2s_1}v^{p_1p_1}_{s_1r_1} \quad (26)$$

$$+ \sum_{\substack{I_{P_1}\in G(A)\\ I_{R_1}\neq I_{R_2}\in G(B)}} \sum_{\substack{p_1 q_1\in I_{P_1}\\ r_1\in I_{R_1} r_2 s_2\in I_{R_2}}} 2c_{p_1}c_{r_2}c_{s_2}c_{q_1}n_{r_1}S_{p_1r_2}S_{s_2q_1}S_{q_1r_1}v^{p_1r_2}_{r_1s_2} \quad (27)$$

$$+ \sum_{\substack{I_{P_1}\neq I_{P_2}\neq I_{P_3}\in G(A)\\ I_{R_1}\in G(B)}} \sum_{\substack{p_1\in I_{P_1} p_2\in I_{P_2} p_3\in I_{P_3}\\ r_1 s_1\in I_{R_1}}} -2n_{p_1}c_{r_1}n_{p_2}c_{s_1}n_{p_3}S_{r_1p_2}S_{p_2s_1}S_{s_1p_3}v^{p_1p_1}_{p_3r_1} \quad (28)$$

$$+ \sum_{\substack{I_{P_1}\neq I_{P_2}\in G(A)\\ I_{R_1}\neq I_{R_2}\in G(B)}} \sum_{\substack{p_1\in I_{P_1} p_2\in I_{P_2}\\ r_1\in I_{R_1} r_2\in I_{R_2}}} -2n_{p_1}n_{r_1}n_{p_2}n_{r_2}S_{r_1p_2}S_{p_2r_2}v^{p_1p_1}_{r_2r_1} \quad (29)$$

$$+ \sum_{\substack{I_{P_1}\neq I_{P_2}\in G(A)\\ I_{R_1}\neq I_{R_2}\in G(B)}} \sum_{\substack{p_1\in I_{P_1} p_2 q_2\in I_{P_2}\\ r_1\in I_{R_1} r_2\in I_{R_2}}} -2n_{p_1}n_{r_1}c_{p_2}n_{r_2}c_{q_2}S_{r_1p_2}S_{p_2r_2}S_{r_2q_2}v^{p_1p_1}_{q_2r_1} \quad (30)$$

$$+ \sum_{\substack{I_{P_1}\neq I_{P_2}\in G(A)\\ I_{R_1}\neq I_{R_2}\in G(B)}} \sum_{\substack{p_1\in I_{P_1} p_2 q_2\in I_{P_2}\\ r_1\in I_{R_1} r_2 s_2\in I_{R_2}}} -2c_{p_2}c_{r_2}c_{s_2}n_{p_1}n_{r_1}c_{q_2}S_{p_2r_2}S_{s_2p_1}S_{p_1r_1}S_{r_1q_2}v^{p_2r_2}_{q_2s_2} \quad (31)$$

$$+ \sum_{\substack{I_{P_1}\neq I_{P_2}\in G(A)\\ I_{R_1}\neq I_{R_2}\in G(B)}} \sum_{\substack{p_1\in I_{P_1} p_2 q_2\in I_{P_2}\\ r_1\in I_{R_1} r_2 s_2\in I_{R_2}}} -2c_{p_2}c_{r_2}c_{s_2}c_{q_2}n_{r_1}n_{p_1}S_{p_2r_2}S_{s_2q_2}S_{q_2r_1}S_{r_1p_1}v^{p_2r_2}_{p_1s_2} \quad (32)$$

$$+ \sum_{\substack{I_{P_1}\neq I_{P_2}\in G(A)\\ I_{R_1}\neq I_{R_2}\in G(B)}} \sum_{\substack{p_1\in I_{P_1} p_2 q_2\in I_{P_2}\\ r_1\in I_{R_1} r_2 s_2\in I_{R_2}}} -2c_{p_2}c_{r_2}n_{r_1}n_{p_1}c_{s_2}c_{q_2}S_{p_2r_2}S_{r_1p_1}S_{p_1s_2}S_{s_2q_2}v^{p_2r_2}_{q_2r_1} \quad (33)$$

$$+ \sum_{\substack{I_{P_1}\neq I_{P_2}\neq I_{P_3}\in G(A)\\ I_{R_1}\neq I_{R_2}\in G(B)}} \sum_{\substack{p_1\in I_{P_1} p_2\in I_{P_2} p_3\in I_{P_3}\\ r_1\in I_{R_1} r_2\in I_{R_2}}} 2n_{p_1}n_{r_1}n_{p_2}n_{r_2}n_{p_3}S_{r_1p_2}S_{p_2r_2}S_{r_2p_3}v^{p_1p_1}_{p_3r_1} \quad (34)$$

$$+ \sum_{\substack{I_{P_1}\neq I_{P_2}\in G(A)\\ I_{R_1}\in G(B)}} \sum_{\substack{p_1\in I_{P_1} p_2\in I_{P_2}\\ r_1 s_1\in I_{R_1}}} 2n_{p_1}c_{r_1}n_{p_2}c_{s_1}S_{p_1r_1}S_{p_2s_1}v^{p_1r_1}_{s_1p_2} \quad (35)$$

$$+ \sum_{\substack{I_{P_1}\in G(A)\\ I_{R_1}\neq I_{R_2}\in G(B)}} \sum_{\substack{p_1 q_1\in I_{P_1}\\ r_1\in I_{R_1} r_2\in I_{R_2}}} 2c_{p_1}n_{r_1}c_{q_1}n_{r_2}S_{p_1r_1}S_{q_1r_2}v^{p_1r_1}_{r_2q_1} \quad (36)$$

$$+\sum_{\substack{I_{P_1}\neq I_{P_2}\neq I_{P_3}\in G(A)\\ I_{R_1}\in G(B)}}\sum_{\substack{p_1\in I_{P_1}p_2\in I_{P_2}p_3\in I_{P_3}\\ r_1s_1\in I_{R_1}}} -2n_{p_1}c_{r_1}n_{p_2}c_{s_1}n_{p_3}S_{p_1r_1}S_{p_2s_1}S_{s_1p_3}v^{p_1r_1}_{p_3p_2} \tag{37}$$

$$+\sum_{\substack{I_{P_1}\neq I_{P_2}\in G(A)\\ I_{R_1}\neq I_{R_2}\in G(B)}}\sum_{\substack{p_1\in I_{P_1}p_2\in I_{P_2}\\ r_1\in I_{R_1}r_2s_2\in I_{R_2}}} -2n_{p_1}c_{r_2}c_{s_2}n_{p_2}n_{r_1}S_{p_1r_2}S_{s_2p_2}S_{p_2r_1}v^{p_1r_2}_{r_1s_2} \tag{38}$$

$$+\sum_{\substack{I_{P_1}\neq I_{P_2}\in G(A)\\ I_{R_1}\neq I_{R_2}\in G(B)}}\sum_{\substack{p_1\in I_{P_1}p_2q_2\in I_{P_2}\\ r_1\in I_{R_1}r_2s_2\in I_{R_2}}} -2n_{p_1}c_{r_2}c_{s_2}c_{p_2}n_{r_1}c_{q_2}S_{p_1r_2}S_{s_2p_2}S_{p_2r_1}S_{r_1q_2}v^{p_1r_2}_{q_2s_2} \tag{39}$$

$$+\sum_{\substack{I_{P_1}\neq I_{P_2}\in G(A)\\ I_{R_1}\neq I_{R_2}\in G(B)}}\sum_{\substack{p_1\in I_{P_1}p_2\in I_{P_2}\\ r_1\in I_{R_1}r_2s_2\in I_{R_2}}} -2n_{p_1}c_{r_2}n_{r_1}n_{p_2}c_{s_2}S_{p_1r_2}S_{r_1p_2}S_{p_2s_2}v^{p_1r_2}_{s_2r_1} \tag{40}$$

$$+\sum_{\substack{I_{P_1}\neq I_{P_2}\in G(A)\\ I_{R_1}\neq I_{R_2}\in G(B)}}\sum_{\substack{p_1\in I_{P_1}p_2q_2\in I_{P_2}\\ r_1\in I_{R_1}r_2\in I_{R_2}}} -2c_{p_2}n_{r_1}n_{p_1}n_{r_2}c_{q_2}S_{p_2r_1}S_{p_1r_2}S_{r_2q_2}v^{p_2r_1}_{q_2p_1} \tag{41}$$

$$+\sum_{\substack{I_{P_1}\neq I_{P_2}\in G(A)\\ I_{R_1}\neq I_{R_2}\in G(B)}}\sum_{\substack{p_1\in I_{P_1}p_2q_2\in I_{P_2}\\ r_1\in I_{R_1}r_2\in I_{R_2}}} -2c_{p_2}n_{r_1}c_{q_2}n_{r_2}n_{p_1}S_{p_2r_1}S_{q_2r_2}S_{r_2p_1}v^{p_2r_1}_{p_1q_2} \tag{42}$$

$$+\sum_{\substack{I_{P_1}\neq I_{P_2}\in G(A)\\ I_{R_1}\neq I_{R_2}\in G(B)}}\sum_{\substack{p_1\in I_{P_1}p_2q_2\in I_{P_2}\\ r_1\in I_{R_1}r_2s_2\in I_{R_2}}} -2c_{p_2}n_{r_1}c_{r_2}n_{p_1}c_{s_2}c_{q_2}S_{p_2r_1}S_{r_2p_1}S_{p_1s_2}S_{s_2q_2}v^{p_2r_1}_{q_2r_2} \tag{43}$$

$$+\sum_{\substack{I_{P_1}\in G(A)\\ I_{R_1}\neq I_{R_2}\neq I_{R_3}\in G(B)}}\sum_{\substack{p_1q_1\in I_{P_1}\\ r_1\in I_{R_1}r_2\in I_{R_2}r_3\in I_{R_3}}} -2c_{p_1}n_{r_1}n_{r_2}c_{q_1}n_{r_3}S_{p_1r_1}S_{r_2q_1}S_{q_1r_3}v^{p_1r_1}_{r_3r_2} \tag{44}$$

$$+\sum_{\substack{I_{P_1}\neq I_{P_2}\neq I_{P_3}\in G(A)\\ I_{R_1}\neq I_{R_2}\in G(B)}}\sum_{\substack{p_1\in I_{P_1}p_2\in I_{P_2}p_3\in I_{P_3}\\ r_1\in I_{R_1}r_2s_2\in I_{R_2}}} 2n_{p_1}c_{r_2}c_{s_2}n_{p_2}n_{r_1}n_{p_3}S_{p_1r_2}S_{s_2p_2}S_{p_2r_1}S_{r_1p_3}v^{p_1r_2}_{p_3s_2} \tag{45}$$

$$+\sum_{\substack{I_{P_1}\neq I_{P_2}\neq I_{P_3}\in G(A)\\ I_{R_1}\neq I_{R_2}\in G(B)}}\sum_{\substack{p_1\in I_{P_1}p_2\in I_{P_2}p_3\in I_{P_3}\\ r_1\in I_{R_1}r_2s_2\in I_{R_2}}} 2n_{p_1}c_{r_2}n_{r_1}n_{p_2}c_{s_2}n_{p_3}S_{p_1r_2}S_{r_1p_2}S_{p_2s_2}S_{s_2p_3}v^{p_1r_2}_{p_3r_1} \tag{46}$$

$$+\sum_{\substack{I_{P_1}\neq I_{P_2}\in G(A)\\ I_{R_1}\neq I_{R_2}\neq I_{R_3}\in G(B)}}\sum_{\substack{p_1\in I_{P_1}p_2q_2\in I_{P_2}\\ r_1\in I_{R_1}r_2\in I_{R_2}r_3\in I_{R_3}}} 2c_{p_2}n_{r_1}n_{r_2}n_{p_1}n_{r_3}c_{q_2}S_{p_2r_1}S_{r_2p_1}S_{p_1r_3}S_{r_3q_2}v^{p_2r_1}_{q_2r_2} \tag{47}$$

$$+\sum_{\substack{I_{P_1}\neq I_{P_2}\in G(A)\\ I_{R_1}\neq I_{R_2}\neq I_{R_3}\in G(B)}}\sum_{\substack{p_1\in I_{P_1}p_2q_2\in I_{P_2}\\ r_1\in I_{R_1}r_2\in I_{R_2}r_3\in I_{R_3}}} 2c_{p_2}n_{r_1}n_{r_2}c_{q_2}n_{r_3}n_{p_1}S_{p_2r_1}S_{r_2q_2}S_{q_2r_3}S_{r_3p_1}v^{p_2r_1}_{p_1r_2} \tag{48}$$

$$+\sum_{\substack{I_{P_1}\neq I_{P_2}\in G(A)\\ I_{R_1}\in G(B)}}\sum_{\substack{p_1\in I_{P_1}p_2q_2\in I_{P_2}\\ r_1s_1\in I_{R_1}}} 2c_{p_2}c_{r_1}n_{p_1}c_{s_1}c_{q_2}S_{p_2r_1}S_{r_1p_1}S_{s_1q_2}v^{p_2p_1}_{q_2s_1} \tag{49}$$

$$+\sum_{\substack{I_{P_1}\in G(A)\\ I_{R_1}\neq I_{R_2}\in G(B)}}\sum_{\substack{p_1q_1\in I_{P_1}\\ r_1\in I_{R_1}r_2\in I_{R_2}}} 2c_{p_1}n_{r_1}c_{q_1}n_{r_2}S_{p_1r_1}S_{r_1q_1}v^{p_1q_1}_{r_2r_2} \tag{50}$$

$$+\sum_{\substack{I_{P_1}\neq I_{P_2}\in G(A)\\ I_{R_1}\neq I_{R_2}\in G(B)}}\sum_{\substack{p_1\in I_{P_1}p_2\in I_{P_2}\\ r_1\in I_{R_1}r_2s_2\in I_{R_2}}} -2n_{p_1}n_{r_1}c_{r_2}n_{p_2}c_{s_2}S_{p_1r_1}S_{r_2p_2}S_{p_2s_2}v^{p_1r_1}_{s_2r_2} \tag{51}$$

$$+\sum_{\substack{I_{P_1}\neq I_{P_2}\in G(A)\\ I_{R_1}\neq I_{R_2}\in G(B)}}\sum_{\substack{p_1\in I_{P_1}p_2q_2\in I_{P_2}\\ r_1\in I_{R_1}r_2\in I_{R_2}}} -2c_{p_2}n_{r_1}c_{q_2}n_{r_2}n_{p_1}S_{p_2r_1}S_{r_1q_2}S_{r_2p_1}v^{p_2q_2}_{p_1r_2} \tag{52}$$

$$+\sum_{\substack{I_{P_1}\neq I_{P_2}\neq I_{P_3}\in G(A)\\ I_{R_1}\neq I_{R_2}\in G(B)}}\sum_{\substack{p_1\in I_{P_1}p_2\in I_{P_2}p_3\in I_{P_3}\\ r_1\in I_{R_1}r_2s_2\in I_{R_2}}} 2n_{p_1}n_{r_1}c_{r_2}n_{p_2}c_{s_2}n_{p_3}S_{p_1r_1}S_{r_2p_2}S_{p_2s_2}S_{s_2p_3}v^{p_1r_1}_{p_3r_2} \tag{53}$$

$$+\sum_{\substack{I_{P_1}\neq I_{P_2}\in G(A)\\ I_{R_1}\neq I_{R_2}\neq I_{R_3}\in G(B)}}\sum_{\substack{p_1\in I_{P_1}p_2\in I_{P_2}\\ r_1\in I_{R_1}r_2\in I_{R_2}r_3\in I_{R_3}}} 2n_{p_1}n_{r_1}n_{r_2}n_{p_2}n_{r_3}S_{p_1r_1}S_{r_2p_2}S_{p_2r_3}v^{p_1r_1}_{r_3r_2} \tag{54}$$

$$+\sum_{\substack{I_{P_1}\neq I_{P_2}\in G(A)\\ I_{R_1}\neq I_{R_2}\neq I_{R_3}\in G(B)}}\sum_{\substack{p_1\in I_{P_1}p_2q_2\in I_{P_2}\\ r_1\in I_{R_1}r_2\in I_{R_2}r_3\in I_{R_3}}} 2n_{p_1}n_{r_1}n_{r_2}c_{p_2}n_{r_3}c_{q_2}S_{p_1r_1}S_{r_2p_2}S_{p_2r_3}S_{r_3q_2}v^{p_1r_1}_{q_2r_2} \tag{55}$$

$$+\sum_{\substack{I_{P_1}\neq I_{P_2}\neq I_{P_3}\in G(A)\\ I_{R_1}\neq I_{R_2}\neq I_{R_3}\in G(B)}}\sum_{\substack{p_1\in I_{P_1}p_2\in I_{P_2}p_3\in I_{P_3}\\ r_1\in I_{R_1}r_2\in I_{R_2}r_3\in I_{R_3}}} -2n_{p_1}n_{r_1}n_{r_2}n_{p_2}n_{r_3}n_{p_3}S_{p_1r_1}S_{r_2p_2}S_{p_2r_3}S_{r_3p_3}v^{p_1r_1}_{p_3r_2} \tag{56}$$

$$+\sum_{\substack{I_{P_1}\neq I_{P_2}\in G(A)\\ I_{R_1}\in G(B)}}\sum_{\substack{p_1\in I_{P_1}p_2q_2\in I_{P_2}\\ r_1s_1\in I_{R_1}}} 2c_{p_2}c_{r_1}n_{p_1}c_{s_1}c_{q_2}S_{p_2r_1}S_{r_1p_1}S_{p_1s_1}v^{p_2s_1}_{q_2q_2} \tag{57}$$

$$+\sum_{\substack{I_{P_1}\in G(A)\\ I_{R_1}\neq I_{R_2}\in G(B)}}\sum_{\substack{p_1q_1\in I_{P_1}\\ r_1\in I_{R_1}r_2s_2\in I_{R_2}}} 2c_{p_1}n_{r_1}c_{q_1}c_{r_2}c_{s_2}S_{p_1r_1}S_{r_1q_1}S_{q_1r_2}v^{p_1r_2}_{s_2s_2} \tag{58}$$

$$+\sum_{\substack{I_{P_1}\neq I_{P_2}\in G(A)\\ I_{R_1}\neq I_{R_2}\in G(B)}}\sum_{\substack{p_1\in I_{P_1}p_2q_2\in I_{P_2}\\ r_1\in I_{R_1}r_2s_2\in I_{R_2}}} -2c_{p_2}c_{r_2}n_{p_1}c_{s_2}n_{r_1}c_{q_2}S_{p_2r_2}S_{r_2p_1}S_{p_1s_2}S_{r_1q_2}v^{p_2s_2}_{q_2r_1} \tag{59}$$

$$+\sum_{\substack{I_{P_1}\neq I_{P_2}\in G(A)\\ I_{R_1}\neq I_{R_2}\in G(B)}}\sum_{\substack{p_1\in I_{P_1}p_2q_2\in I_{P_2}\\ r_1\in I_{R_1}r_2s_2\in I_{R_2}}} -2c_{p_2}n_{r_1}c_{q_2}c_{r_2}c_{s_2}n_{p_1}S_{p_2r_1}S_{r_1q_2}S_{q_2r_2}S_{s_2p_1}v^{p_2r_2}_{p_1s_2} \tag{60}$$

$$+\sum_{\substack{I_{P_1}\neq I_{P_2}\in G(A)\\ I_{R_1}\neq I_{R_2}\in G(B)}}\sum_{\substack{p_1\in I_{P_1}p_2\in I_{P_2}\\ r_1\in I_{R_1}r_2s_2\in I_{R_2}}} -2n_{p_1}c_{r_2}n_{p_2}c_{s_2}n_{r_1}S_{p_1r_2}S_{r_2p_2}S_{p_2s_2}v^{p_1s_2}_{r_1r_1} \tag{61}$$

$$+\sum_{\substack{I_{P_1}\neq I_{P_2}\in G(A)\\ I_{R_1}\neq I_{R_2}\in G(B)}}\sum_{\substack{p_1\in I_{P_1}p_2\in I_{P_2}\\ r_1\in I_{R_1}r_2\in I_{R_2}}} -2n_{p_1}n_{r_1}n_{p_2}n_{r_2}S_{p_1r_1}S_{r_1p_2}v^{p_1p_2}_{r_2r_2} \tag{62}$$

$$+\sum_{\substack{I_{P_1}\neq I_{P_2}\in G(A)\\ I_{R_1}\neq I_{R_2}\in G(B)}}\sum_{\substack{p_1\in I_{P_1}p_2q_2\in I_{P_2}\\ r_1\in I_{R_1}r_2s_2\in I_{R_2}}} -2n_{p_1}n_{r_1}c_{p_2}c_{r_2}c_{s_2}c_{q_2}S_{p_1r_1}S_{r_1p_2}S_{p_2r_2}S_{s_2q_2}v^{p_1r_2}_{q_2s_2} \tag{63}$$

$$+\sum_{\substack{I_{P_1}\neq I_{P_2}\in G(A)\\ I_{R_1}\neq I_{R_2}\in G(B)}}\sum_{\substack{p_1\in I_{P_1}p_2q_2\in I_{P_2}\\ r_1\in I_{R_1}r_2s_2\in I_{R_2}}} -2c_{p_2}c_{r_2}n_{p_1}n_{r_1}c_{s_2}c_{q_2}S_{p_2r_2}S_{r_2p_1}S_{p_1r_1}S_{s_2q_2}v^{p_2r_1}_{q_2s_2} \tag{64}$$

$$+\sum_{\substack{I_{P_1}\neq I_{P_2}\in G(A)\\ I_{R_1}\neq I_{R_2}\in G(B)}}\sum_{\substack{p_1\in I_{P_1}p_2q_2\in I_{P_2}\\ r_1\in I_{R_1}r_2s_2\in I_{R_2}}} -2c_{p_2}n_{r_1}n_{p_1}c_{r_2}c_{s_2}c_{q_2}S_{p_2r_1}S_{r_1p_1}S_{p_1r_2}S_{s_2q_2}v^{p_2r_2}_{q_2s_2} \tag{65}$$

$$+\sum_{\substack{I_{P_1}\in G(A)\\ I_{R_1}\neq I_{R_2}\neq I_{R_3}\in G(B)}}\sum_{\substack{p_1q_1\in I_{P_1}\\ r_1\in I_{R_1}r_2\in I_{R_2}r_3\in I_{R_3}}} -2c_{p_1}n_{r_1}c_{q_1}n_{r_2}n_{r_3}S_{p_1r_1}S_{r_1q_1}S_{q_1r_2}v^{p_1r_2}_{r_3r_3} \tag{66}$$

$$+\sum_{\substack{I_{P_1}\neq I_{P_2}\neq I_{P_3}\in G(A)\\ I_{R_1}\neq I_{R_2}\in G(B)}}\sum_{\substack{p_1\in I_{P_1}p_2\in I_{P_2}p_3\in I_{P_3}\\ r_1\in I_{R_1}r_2s_2\in I_{R_2}}} 2n_{p_1}c_{r_2}n_{p_2}c_{s_2}n_{r_1}n_{p_3}S_{p_1r_2}S_{r_2p_2}S_{p_2s_2}S_{r_1p_3}v^{p_1s_2}_{p_3r_1} \tag{67}$$

$$+\sum_{\substack{I_{P_1}\neq I_{P_2}\neq I_{P_3}\in G(A)\\ I_{R_1}\neq I_{R_2}\in G(B)}}\sum_{\substack{p_1\in I_{P_1}p_2\in I_{P_2}p_3\in I_{P_3}\\ r_1\in I_{R_1}r_2\in I_{R_2}}} 2n_{p_1}n_{r_1}n_{p_2}n_{r_2}n_{p_3}S_{p_1r_1}S_{r_1p_2}S_{r_2p_3}v^{p_1p_2}_{p_3r_2} \tag{68}$$

$$+\sum_{\substack{I_{P_1}\neq I_{P_2}\in G(A)\\ I_{R_1}\neq I_{R_2}\neq I_{R_3}\in G(B)}}\sum_{\substack{p_1\in I_{P_1}p_2q_2\in I_{P_2}\\ r_1\in I_{R_1}r_2\in I_{R_2}r_3\in I_{R_3}}} 2c_{p_2}n_{r_1}c_{q_2}n_{r_2}n_{r_3}n_{p_1}S_{p_2r_1}S_{r_1q_2}S_{q_2r_2}S_{r_3p_1}v^{p_2r_2}_{p_1r_3} \tag{69}$$

$$+\sum_{\substack{I_{P_1}\neq I_{P_2}\in G(A)\\ I_{R_1}\neq I_{R_2}\in G(B)}}\sum_{\substack{p_1\in I_{P_1}p_2\in I_{P_2}\\ r_1\in I_{R_1}r_2s_2\in I_{R_2}}} -2n_{p_1}c_{r_2}n_{p_2}n_{r_1}c_{s_2}S_{p_1r_2}S_{r_2p_2}S_{p_2r_1}v^{p_1r_1}_{s_2s_2} \tag{70}$$

$$+\sum_{\substack{I_{P_1}\neq I_{P_2}\in G(A)\\ I_{R_1}\neq I_{R_2}\in G(B)}}\sum_{\substack{p_1\in I_{P_1}p_2\in I_{P_2}\\ r_1\in I_{R_1}r_2s_2\in I_{R_2}}} -2n_{p_1}n_{r_1}n_{p_2}c_{r_2}c_{s_2}S_{p_1r_1}S_{r_1p_2}S_{p_2r_2}v^{p_1r_2}_{s_2s_2} \tag{71}$$

$$+\sum_{\substack{I_{P_1}\neq I_{P_2}\in G(A)\\ I_{R_1}\neq I_{R_2}\in G(B)}}\sum_{\substack{p_1\in I_{P_1}p_2q_2\in I_{P_2}\\ r_1\in I_{R_1}r_2\in I_{R_2}}} -2n_{p_1}n_{r_1}c_{p_2}n_{r_2}c_{q_2}S_{p_1r_1}S_{r_1p_2}S_{p_2r_2}v^{p_1r_2}_{q_2q_2} \tag{72}$$

$$+\sum_{\substack{I_{P_1}\neq I_{P_2}\in G(A)\\ I_{R_1}\neq I_{R_2}\in G(B)}}\sum_{\substack{p_1\in I_{P_1}p_2q_2\in I_{P_2}\\ r_1\in I_{R_1}r_2\in I_{R_2}}} -2c_{p_2}n_{r_1}n_{p_1}n_{r_2}c_{q_2}S_{p_2r_1}S_{r_1p_1}S_{p_1r_2}v^{p_2r_2}_{q_2q_2} \tag{73}$$

$$+\sum_{\substack{I_{P_1}\neq I_{P_2}\neq I_{P_3}\in G(A)\\ I_{R_1}\neq I_{R_2}\in G(B)}}\sum_{\substack{p_1\in I_{P_1}p_2\in I_{P_2}p_3\in I_{P_3}\\ r_1\in I_{R_1}r_2s_2\in I_{R_2}}} 2n_{p_1}c_{r_2}n_{p_2}n_{r_1}c_{s_2}n_{p_3}S_{p_1r_2}S_{r_2p_2}S_{p_2r_1}S_{s_2p_3}v^{p_1r_1}_{p_3s_2} \tag{74}$$

$$+\sum_{\substack{I_{P_1}\neq I_{P_2}\neq I_{P_3}\in G(A)\\ I_{R_1}\neq I_{R_2}\in G(B)}}\sum_{\substack{p_1\in I_{P_1}p_2\in I_{P_2}p_3\in I_{P_3}\\ r_1\in I_{R_1}r_2s_2\in I_{R_2}}} 2n_{p_1}n_{r_1}n_{p_2}c_{r_2}c_{s_2}n_{p_3}S_{p_1r_1}S_{r_1p_2}S_{p_2r_2}S_{s_2p_3}v^{p_1r_2}_{p_3s_2} \tag{75}$$

$$+\sum_{\substack{I_{P_1}\neq I_{P_2}\in G(A)\\ I_{R_1}\neq I_{R_2}\neq I_{R_3}\in G(B)}}\sum_{\substack{p_1\in I_{P_1}p_2q_2\in I_{P_2}\\ r_1\in I_{R_1}r_2\in I_{R_2}r_3\in I_{R_3}}} 2n_{p_1}n_{r_1}c_{p_2}n_{r_2}n_{r_3}c_{q_2}S_{p_1r_1}S_{r_1p_2}S_{p_2r_2}S_{r_3q_2}v^{p_1r_2}_{q_2r_3} \tag{76}$$

$$+\sum_{\substack{I_{P_1}\neq I_{P_2}\in G(A)\\ I_{R_1}\neq I_{R_2}\neq I_{R_3}\in G(B)}}\sum_{\substack{p_1\in I_{P_1}p_2q_2\in I_{P_2}\\ r_1\in I_{R_1}r_2\in I_{R_2}r_3\in I_{R_3}}} 2c_{p_2}n_{r_1}n_{p_1}n_{r_2}n_{r_3}c_{q_2}S_{p_2r_1}S_{r_1p_1}S_{p_1r_2}S_{r_3q_2}v^{p_2r_2}_{q_2r_3} \tag{77}$$

$$+\sum_{\substack{I_{P_1}\neq I_{P_2}\in G(A)\\ I_{R_1}\neq I_{R_2}\neq I_{R_3}\in G(B)}}\sum_{\substack{p_1\in I_{P_1}p_2\in I_{P_2}\\ r_1\in I_{R_1}r_2\in I_{R_2}r_3\in I_{R_3}}} 2n_{p_1}n_{r_1}n_{p_2}n_{r_2}n_{r_3}S_{p_1r_1}S_{r_1p_2}S_{p_2r_2}v^{p_1r_2}_{r_3r_3} \tag{78}$$

$$+\sum_{\substack{I_{P_1}\neq I_{P_2}\neq I_{P_3}\in G(A)\\ I_{R_1}\neq I_{R_2}\neq I_{R_3}\in G(B)}}\sum_{\substack{p_1\in I_{P_1}p_2\in I_{P_2}p_3\in I_{P_3}\\ r_1\in I_{R_1}r_2\in I_{R_2}r_3\in I_{R_3}}} -2n_{p_1}n_{r_1}n_{p_2}n_{r_2}n_{r_3}n_{p_3}S_{p_1r_1}S_{r_1p_2}S_{p_2r_2}S_{r_3p_3}v^{p_1r_2}_{p_3r_3} \quad (79)$$

$$\langle \hat{V}_{ee}\mathcal{P}_4\rangle^a_{\text{2-loops}} =$$

$$\sum_{\substack{I_{P_1}\in G(A)\\ I_{R_1}\in G(B)}}\sum_{\substack{p_1q_1\in I_{P_1}\\ r_1s_1\in I_{R_1}}} 4c_{p_1}c_{r_1}c_{s_1}c_{q_1}S_{p_1r_1}S_{s_1q_1}v^{p_1s_1}_{r_1q_1} \tag{80}$$

$$+\sum_{\substack{I_{P_1}\in G(A)\\ I_{R_1}\neq I_{R_2}\in G(B)}}\sum_{\substack{p_1q_1\in I_{P_1}\\ r_1\in I_{R_1}r_2s_2\in I_{R_2}}} -4c_{p_1}c_{r_2}c_{s_2}c_{q_1}n_{r_1}S_{p_1r_2}S_{s_2q_1}S_{q_1r_1}v^{p_1s_2}_{r_2r_1} \tag{81}$$

$$+\sum_{\substack{I_{P_1}\neq I_{P_2}\neq I_{P_3}\in G(A)\\ I_{R_1}\in G(B)}}\sum_{\substack{p_1\in I_{P_1}p_2\in I_{P_2}p_3\in I_{P_3}\\ r_1s_1\in I_{R_1}}} 4n_{p_1}c_{r_1}n_{p_2}c_{s_1}n_{p_3}S_{r_1p_2}S_{p_2s_1}S_{s_1p_3}v^{p_1r_1}_{p_1p_3} \tag{82}$$

$$+\sum_{\substack{I_{P_1}\neq I_{P_2}\in G(A)\\ I_{R_1}\neq I_{R_2}\in G(B)}}\sum_{\substack{p_1\in I_{P_1}p_2q_2\in I_{P_2}\\ r_1\in I_{R_1}r_2s_2\in I_{R_2}}} 4n_{p_1}c_{r_2}c_{p_2}n_{r_1}c_{q_2}c_{s_2}S_{r_2p_2}S_{p_2r_1}S_{r_1q_2}S_{q_2s_2}v^{p_1r_2}_{p_1s_2} \tag{83}$$

$$+\sum_{\substack{I_{P_1}\neq I_{P_2}\in G(A)\\ I_{R_1}\neq I_{R_2}\in G(B)}}\sum_{\substack{p_1\in I_{P_1}p_2q_2\in I_{P_2}\\ r_1\in I_{R_1}r_2\in I_{R_2}}} 4n_{p_1}n_{r_1}c_{p_2}n_{r_2}c_{q_2}S_{r_1p_2}S_{p_2r_2}S_{r_2q_2}v^{p_1r_1}_{p_1q_2} \tag{84}$$

$$+\sum_{\substack{I_{P_1}\neq I_{P_2}\neq I_{P_3}\in G(A)\\ I_{R_1}\neq I_{R_2}\in G(B)}}\sum_{\substack{p_1\in I_{P_1}p_2\in I_{P_2}p_3\in I_{P_3}\\ r_1\in I_{R_1}r_2s_2\in I_{R_2}}} -4n_{p_1}c_{r_2}n_{p_2}c_{s_2}n_{p_3}n_{r_1}S_{r_2p_2}S_{p_2s_2}S_{s_2p_3}S_{p_3r_1}v^{p_1r_2}_{p_1r_1} \tag{85}$$

$$+\sum_{\substack{I_{P_1}\neq I_{P_2}\neq I_{P_3}\in G(A)\\ I_{R_1}\neq I_{R_2}\in G(B)}}\sum_{\substack{p_1\in I_{P_1}p_2\in I_{P_2}p_3\in I_{P_3}\\ r_1\in I_{R_1}r_2s_2\in I_{R_2}}} -4n_{p_1}c_{r_2}n_{p_2}n_{r_1}n_{p_3}c_{s_2}S_{r_2p_2}S_{p_2r_1}S_{r_1p_3}S_{p_3s_2}v^{p_1r_2}_{p_1s_2} \tag{86}$$

$$+\sum_{\substack{I_{P_1}\neq I_{P_2}\neq I_{P_3}\in G(A)\\ I_{R_1}\neq I_{R_2}\in G(B)}}\sum_{\substack{p_1\in I_{P_1}p_2\in I_{P_2}p_3\in I_{P_3}\\ r_1\in I_{R_1}r_2s_2\in I_{R_2}}} -4n_{p_1}n_{r_1}n_{p_2}c_{r_2}n_{p_3}c_{s_2}S_{r_1p_2}S_{p_2r_2}S_{r_2p_3}S_{p_3s_2}v^{p_1r_1}_{p_1s_2} \tag{87}$$

$$+\sum_{\substack{I_{P_1}\neq I_{P_2}\neq I_{P_3}\in G(A)\\ I_{R_1}\neq I_{R_2}\in G(B)}}\sum_{\substack{p_1\in I_{P_1}p_2\in I_{P_2}p_3\in I_{P_3}\\ r_1\in I_{R_1}r_2\in I_{R_2}}} -4n_{p_1}n_{r_1}n_{p_2}n_{r_2}n_{p_3}S_{r_1p_2}S_{p_2r_2}S_{r_2p_3}v^{p_1r_1}_{p_1p_3} \tag{88}$$

$$+\sum_{\substack{I_{P_1}\neq I_{P_2}\in G(A)\\ I_{R_1}\neq I_{R_2}\neq I_{R_3}\in G(B)}}\sum_{\substack{p_1\in I_{P_1}p_2q_2\in I_{P_2}\\ r_1\in I_{R_1}r_2\in I_{R_2}r_3\in I_{R_3}}} -4n_{p_1}n_{r_1}c_{p_2}n_{r_2}c_{q_2}n_{r_3}S_{r_1p_2}S_{p_2r_2}S_{r_2q_2}S_{q_2r_3}v^{p_1r_1}_{p_1r_3} \tag{89}$$

$$+\sum_{\substack{I_{P_1}\neq I_{P_2}\neq I_{P_3}\in G(A)\\ I_{R_1}\neq I_{R_2}\neq I_{R_3}\in G(B)}}\sum_{\substack{p_1\in I_{P_1}p_2\in I_{P_2}p_3\in I_{P_3}\\ r_1\in I_{R_1}r_2\in I_{R_2}r_3\in I_{R_3}}} 4n_{p_1}n_{r_1}n_{p_2}n_{r_2}n_{p_3}n_{r_3}S_{r_1p_2}S_{p_2r_2}S_{r_2p_3}S_{p_3r_3}v^{p_1r_1}_{p_1r_3} \tag{90}$$

$$+\sum_{\substack{I_{P_1}\neq I_{P_2}\in G(A)\\ I_{R_1}\in G(B)}}\sum_{\substack{p_1\in I_{P_1}p_2q_2\in I_{P_2}\\ r_1s_1\in I_{R_1}}} -4c_{p_2}c_{r_1}n_{p_1}c_{s_1}c_{q_2}S_{p_2r_1}S_{r_1p_1}S_{s_1q_2}v^{p_2s_1}_{p_1q_2} \tag{91}$$

$$+\sum_{\substack{I_{P_1}\neq I_{P_2}\in G(A)\\ I_{R_1}\neq I_{R_2}\in G(B)}}\sum_{\substack{p_1\in I_{P_1}p_2q_2\in I_{P_2}\\ r_1\in I_{R_1}r_2s_2\in I_{R_2}}} 4n_{p_1}c_{r_2}c_{p_2}n_{r_1}c_{q_2}c_{s_2}S_{p_1r_2}S_{r_2p_2}S_{r_1q_2}S_{q_2s_2}v^{p_1r_1}_{p_2s_2} \tag{92}$$

$$+\sum_{\substack{I_{P_1}\neq I_{P_2}\in G(A)\\ I_{R_1}\neq I_{R_2}\in G(B)}}\sum_{\substack{p_1\in I_{P_1}p_2\in I_{P_2}\\ r_1\in I_{R_1}r_2s_2\in I_{R_2}}} 4n_{p_1}n_{r_1}c_{r_2}n_{p_2}c_{s_2}S_{p_1r_1}S_{r_2p_2}S_{p_2s_2}v^{p_1r_2}_{r_1s_2} \tag{93}$$

$$+\sum_{\substack{I_{P_1}\neq I_{P_2}\in G(A)\\ I_{R_1}\neq I_{R_2}\in G(B)}}\sum_{\substack{p_1\in I_{P_1}p_2\in I_{P_2}\\ r_1\in I_{R_1}r_2\in I_{R_2}}} 4n_{p_1}n_{r_1}n_{r_2}n_{p_2}S_{p_1r_1}S_{r_2p_2}v^{p_1r_2}_{r_1p_2} \tag{94}$$

$$+\sum_{\substack{I_{P_1}\neq I_{P_2}\in G(A)\\ I_{R_1}\neq I_{R_2}\in G(B)}}\sum_{\substack{p_1\in I_{P_1}p_2q_2\in I_{P_2}\\ r_1\in I_{R_1}r_2s_2\in I_{R_2}}} 4c_{p_2}c_{r_2}n_{p_1}c_{s_2}c_{q_2}n_{r_1}S_{p_2r_2}S_{r_2p_1}S_{s_2q_2}S_{q_2r_1}v^{p_2s_2}_{p_1r_1} \tag{95}$$

$$+\sum_{\substack{I_{P_1}\neq I_{P_2}\in G(A)\\ I_{R_1}\neq I_{R_2}\in G(B)}}\sum_{\substack{p_1\in I_{P_1}p_2q_2\in I_{P_2}\\ r_1\in I_{R_1}r_2s_2\in I_{R_2}}} 4c_{p_2}n_{r_1}c_{q_2}c_{r_2}n_{p_1}c_{s_2}S_{p_2r_1}S_{r_1q_2}S_{r_2p_1}S_{p_1s_2}v^{p_2r_2}_{q_2s_2} \tag{96}$$

$$+\sum_{\substack{I_{P_1}\neq I_{P_2}\in G(A)\\ I_{R_1}\neq I_{R_2}\in G(B)}}\sum_{\substack{p_1\in I_{P_1}p_2q_2\in I_{P_2}\\ r_1\in I_{R_1}r_2\in I_{R_2}}} 4c_{p_2}n_{r_1}c_{q_2}n_{r_2}n_{p_1}S_{p_2r_1}S_{r_1q_2}S_{r_2p_1}v^{p_2r_2}_{q_2p_1} \tag{97}$$

$$+\sum_{\substack{I_{P_1}\neq I_{P_2}\in G(A)\\ I_{R_1}\neq I_{R_2}\neq I_{R_3}\in G(B)}}\sum_{\substack{p_1\in I_{P_1}p_2\in I_{P_2}\\ r_1\in I_{R_1}r_2\in I_{R_2}r_3\in I_{R_3}}} -4n_{p_1}n_{r_1}n_{r_2}n_{p_2}n_{r_3}S_{p_1r_1}S_{r_2p_2}S_{p_2r_3}v^{p_1r_2}_{r_1r_3} \tag{98}$$

$$+\sum_{\substack{I_{P_1}\neq I_{P_2}\in G(A)\\ I_{R_1}\neq I_{R_2}\neq I_{R_3}\in G(B)}}\sum_{\substack{p_1\in I_{P_1}p_2q_2\in I_{P_2}\\ r_1\in I_{R_1}r_2\in I_{R_2}r_3\in I_{R_3}}} -4c_{p_2}n_{r_1}c_{q_2}n_{r_2}n_{p_1}n_{r_3}S_{p_2r_1}S_{r_1q_2}S_{r_2p_1}S_{p_1r_3}v^{p_2r_2}_{q_2r_3} \tag{99}$$

$$+\sum_{\substack{I_{P_1}\neq I_{P_2}\in G(A)\\ I_{R_1}\neq I_{R_2}\in G(B)}}\sum_{\substack{p_1\in I_{P_1}p_2\in I_{P_2}\\ r_1\in I_{R_1}r_2s_2\in I_{R_2}}} 4n_{p_1}c_{r_2}n_{p_2}c_{s_2}n_{r_1}S_{p_1r_2}S_{r_2p_2}S_{p_2s_2}v^{p_1r_1}_{s_2r_1} \tag{100}$$

$$+\sum_{\substack{I_{P_1}\neq I_{P_2}\in G(A)\\ I_{R_1}\neq I_{R_2}\in G(B)}}\sum_{\substack{p_1\in I_{P_1}p_2q_2\in I_{P_2}\\ r_1\in I_{R_1}r_2s_2\in I_{R_2}}} 4c_{p_2}c_{r_2}n_{p_1}c_{s_2}c_{q_2}n_{r_1}S_{p_2r_2}S_{r_2p_1}S_{p_1s_2}S_{s_2q_2}v^{p_2r_1}_{q_2r_1} \tag{101}$$

$$+\sum_{\substack{I_{P_1}\in G(A)\\ I_{R_1}\neq I_{R_2}\neq I_{R_3}\in G(B)}}\sum_{\substack{p_1q_1\in I_{P_1}\\ r_1\in I_{R_1}r_2\in I_{R_2}r_3\in I_{R_3}}} 4c_{p_1}n_{r_1}c_{q_1}n_{r_2}n_{r_3}S_{p_1r_1}S_{r_1q_1}S_{q_1r_2}v^{p_1r_3}_{r_2r_3} \tag{102}$$

$$+\sum_{\substack{I_{P_1}\neq I_{P_2}\neq I_{P_3}\in G(A)\\ I_{R_1}\neq I_{R_2}\in G(B)}}\sum_{\substack{p_1\in I_{P_1}p_2\in I_{P_2}p_3\in I_{P_3}\\ r_1\in I_{R_1}r_2s_2\in I_{R_2}}} -4n_{p_1}n_{r_1}n_{p_2}c_{r_2}n_{p_3}c_{s_2}S_{p_1r_1}S_{r_1p_2}S_{r_2p_3}S_{p_3s_2}v^{p_1r_2}_{p_2s_2} \tag{103}$$

$$+\sum_{\substack{I_{P_1}\neq I_{P_2}\neq I_{P_3}\in G(A)\\ I_{R_1}\neq I_{R_2}\in G(B)}}\sum_{\substack{p_1\in I_{P_1}p_2\in I_{P_2}p_3\in I_{P_3}\\ r_1\in I_{R_1}r_2\in I_{R_2}}} -4n_{p_1}n_{r_1}n_{p_2}n_{r_2}n_{p_3}S_{p_1r_1}S_{r_1p_2}S_{r_2p_3}v^{p_1r_2}_{p_2p_3} \tag{104}$$

$$+\sum_{\substack{I_{P_1}\neq I_{P_2}\neq I_{P_3}\in G(A)\\ I_{R_1}\neq I_{R_2}\neq I_{R_3}\in G(B)}}\sum_{\substack{p_1\in I_{P_1}p_2\in I_{P_2}p_3\in I_{P_3}\\ r_1\in I_{R_1}r_2\in I_{R_2}r_3\in I_{R_3}}} 4n_{p_1}n_{r_1}n_{p_2}n_{r_2}n_{p_3}n_{r_3}S_{p_1r_1}S_{r_1p_2}S_{r_2p_3}S_{p_3r_3}v^{p_1r_2}_{p_2r_3} \tag{105}$$

$$+\sum_{\substack{I_{P_1}\neq I_{P_2}\neq I_{P_3}\in G(A)\\ I_{R_1}\neq I_{R_2}\in G(B)}}\sum_{\substack{p_1\in I_{P_1}p_2\in I_{P_2}p_3\in I_{P_3}\\ r_1\in I_{R_1}r_2s_2\in I_{R_2}}} -4n_{p_1}c_{r_2}n_{p_2}c_{s_2}n_{p_3}n_{r_1}S_{p_1r_2}S_{r_2p_2}S_{p_2s_2}S_{s_2p_3}v^{p_1r_1}_{p_3r_1} \tag{106}$$

$$+ \sum_{\substack{I_{P_1} \neq I_{P_2} \in G(A) \\ I_{R_1} \neq I_{R_2} \neq I_{R_3} \in G(B)}} \sum_{\substack{p_1 \in I_{P_1} p_2 \in I_{P_2} \\ r_1 \in I_{R_1} r_2 \in I_{R_2} r_3 \in I_{R_3}}} -4 n_{p_1} n_{r_1} n_{p_2} n_{r_2} n_{r_3} S_{p_1 r_1} S_{r_1 p_2} S_{p_2 r_2} v^{p_1 r_3}_{r_2 r_3} \tag{107}$$

$$+ \sum_{\substack{I_{P_1} \neq I_{P_2} \in G(A) \\ I_{R_1} \neq I_{R_2} \neq I_{R_3} \in G(B)}} \sum_{\substack{p_1 \in I_{P_1} p_2 q_2 \in I_{P_2} \\ r_1 \in I_{R_1} r_2 \in I_{R_2} r_3 \in I_{R_3}}} -4 n_{p_1} n_{r_1} c_{p_2} n_{r_2} c_{q_2} n_{r_3} S_{p_1 r_1} S_{r_1 p_2} S_{p_2 r_2} S_{r_2 q_2} v^{p_1 r_3}_{q_2 r_3} \tag{108}$$

$$+ \sum_{\substack{I_{P_1} \neq I_{P_2} \in G(A) \\ I_{R_1} \neq I_{R_2} \neq I_{R_3} \in G(B)}} \sum_{\substack{p_1 \in I_{P_1} p_2 q_2 \in I_{P_2} \\ r_1 \in I_{R_1} r_2 \in I_{R_2} r_3 \in I_{R_3}}} -4 c_{p_2} n_{r_1} n_{p_1} n_{r_2} c_{q_2} n_{r_3} S_{p_2 r_1} S_{r_1 p_1} S_{p_1 r_2} S_{r_2 q_2} v^{p_2 r_3}_{q_2 r_3} \tag{109}$$

$$+ \sum_{\substack{I_{P_1} \neq I_{P_2} \in G(A) \\ I_{R_1} \neq I_{R_2} \neq I_{R_3} \in G(B)}} \sum_{\substack{p_1 \in I_{P_1} p_2 q_2 \in I_{P_2} \\ r_1 \in I_{R_1} r_2 \in I_{R_2} r_3 \in I_{R_3}}} -4 c_{p_2} n_{r_1} c_{q_2} n_{r_2} n_{p_1} n_{r_3} S_{p_2 r_1} S_{r_1 q_2} S_{q_2 r_2} S_{r_2 p_1} v^{p_2 r_3}_{p_1 r_3} \tag{110}$$

$$+ \sum_{\substack{I_{P_1} \neq I_{P_2} \neq I_{P_3} \in G(A) \\ I_{R_1} \neq I_{R_2} \neq I_{R_3} \in G(B)}} \sum_{\substack{p_1 \in I_{P_1} p_2 \in I_{P_2} p_3 \in I_{P_3} \\ r_1 \in I_{R_1} r_2 \in I_{R_2} r_3 \in I_{R_3}}} 4 n_{p_1} n_{r_1} n_{p_2} n_{r_2} n_{p_3} n_{r_3} S_{p_1 r_1} S_{r_1 p_2} S_{p_2 r_2} S_{r_2 p_3} v^{p_1 r_3}_{p_3 r_3} \tag{111}$$

$$\langle \hat{V}_{ee}\mathcal{P}_4\rangle^{b}_{\text{2-loops}} =$$

$$\frac{1}{2}\sum_{\substack{I_{P_1}\neq I_{P_2}\in G(A)\\ I_{R_1}\in G(B)}}\sum_{\substack{p_1\in I_{P_1}p_2q_2\in I_{P_2}\\ r_1s_1\in I_{R_1}}} -4n_{p_1}c_{r_1}c_{p_2}c_{q_2}c_{s_1}S_{r_1p_2}v^{p_1p_1}_{p_2r_1}S_{q_2s_1}S_{q_2s_1} \tag{112}$$

$$+\frac{1}{2}\sum_{\substack{I_{P_1}\in G(A)\\ I_{R_1}\neq I_{R_2}\in G(B)}}\sum_{\substack{p_1q_1\in I_{P_1}\\ r_1\in I_{R_1}r_2s_2\in I_{R_2}}} -4c_{p_1}c_{r_2}n_{r_1}c_{s_2}c_{q_1}S_{p_1r_2}v^{p_1r_2}_{r_1r_1}S_{s_2q_1}S_{q_1s_2} \tag{113}$$

$$+\sum_{\substack{I_{P_1}\neq I_{P_2}\in G(A)\\ I_{R_1}\neq I_{R_2}\in G(B)}}\sum_{\substack{p_1\in I_{P_1}p_2\in I_{P_2}\\ r_1\in I_{R_1}r_2\in I_{R_2}}} 4n_{p_1}n_{r_1}n_{r_2}n_{p_2}v^{p_1p_1}_{r_1r_1}S_{r_2p_2}S_{p_2r_2} \tag{114}$$

$$+\sum_{\substack{I_{P_1}\neq I_{P_2}\in G(A)\\ I_{R_1}\neq I_{R_2}\in G(B)}}\sum_{\substack{p_1\in I_{P_1}p_2q_2\in I_{P_2}\\ r_1\in I_{R_1}r_2s_2\in I_{R_2}}} 4c_{p_2}c_{r_2}c_{s_2}c_{q_2}n_{r_1}n_{p_1}S_{p_2r_2}S_{s_2q_2}v^{p_2r_2}_{q_2s_2}S_{r_1p_1}S_{p_1r_1} \tag{115}$$

$$+\frac{1}{2}\sum_{\substack{I_{P_1}\neq I_{P_2}\in G(A)\\ I_{R_1}\neq I_{R_2}\in G(B)}}\sum_{\substack{p_1\in I_{P_1}p_2q_2\in I_{P_2}\\ r_1\in I_{R_1}r_2s_2\in I_{R_2}}} 4c_{p_2}c_{r_2}n_{r_1}n_{p_1}c_{s_2}c_{q_2}S_{p_2r_2}S_{r_1p_1}v^{p_2r_2}_{p_1r_1}S_{s_2q_2}S_{q_2s_2} \tag{116}$$

$$+\sum_{\substack{I_{P_1}\neq I_{P_2}\neq I_{P_3}\in G(A)\\ I_{R_1}\neq I_{R_2}\in G(B)}}\sum_{\substack{p_1\in I_{P_1}p_2\in I_{P_2}p_3\in I_{P_3}\\ r_1\in I_{R_1}r_2\in I_{R_2}}} -4n_{p_1}n_{r_1}n_{p_2}n_{r_2}n_{p_3}S_{r_1p_2}v^{p_1p_1}_{p_2r_1}S_{r_2p_3}S_{p_3r_2} \tag{117}$$

$$+\frac{1}{2}\sum_{\substack{I_{P_1}\neq I_{P_2}\in G(A)\\ I_{R_1}\in G(B)}}\sum_{\substack{p_1\in I_{P_1}p_2q_2\in I_{P_2}\\ r_1s_1\in I_{R_1}}} -4n_{p_1}c_{r_1}c_{p_2}c_{q_2}c_{s_1}S_{p_1r_1}v^{p_1r_1}_{p_2p_2}S_{q_2s_1}S_{q_2s_1} \tag{118}$$

$$+\frac{1}{2}\sum_{\substack{I_{P_1}\in G(A)\\ I_{R_1}\neq I_{R_2}\in G(B)}}\sum_{\substack{p_1q_1\in I_{P_1}\\ r_1\in I_{R_1}r_2s_2\in I_{R_2}}} -4c_{p_1}n_{r_1}c_{r_2}c_{s_2}c_{q_1}S_{p_1r_1}v^{p_1r_1}_{r_2r_2}S_{s_2q_1}S_{q_1s_2} \tag{119}$$

$$+\sum_{\substack{I_{P_1}\neq I_{P_2}\in G(A)\\ I_{R_1}\neq I_{R_2}\in G(B)}}\sum_{\substack{p_1\in I_{P_1}p_2\in I_{P_2}\\ r_1\in I_{R_1}r_2s_2\in I_{R_2}}} 4n_{p_1}c_{r_2}c_{s_2}n_{r_1}n_{p_2}S_{p_1r_2}v^{p_1r_2}_{s_2s_2}S_{r_1p_2}S_{p_2r_1} \tag{120}$$

$$+\sum_{\substack{I_{P_1}\neq I_{P_2}\in G(A)\\ I_{R_1}\neq I_{R_2}\in G(B)}}\sum_{\substack{p_1\in I_{P_1}p_2q_2\in I_{P_2}\\ r_1\in I_{R_1}r_2\in I_{R_2}}} 4c_{p_2}n_{r_1}c_{q_2}n_{r_2}n_{p_1}S_{p_2r_1}v^{p_2r_1}_{q_2q_2}S_{r_2p_1}S_{p_1r_2} \tag{121}$$

$$+\frac{1}{2}\sum_{\substack{I_{P_1}\neq I_{P_2}\in G(A)\\ I_{R_1}\neq I_{R_2}\in G(B)}}\sum_{\substack{p_1\in I_{P_1}p_2q_2\in I_{P_2}\\ r_1\in I_{R_1}r_2s_2\in I_{R_2}}} 4n_{p_1}c_{r_2}n_{r_1}c_{p_2}c_{q_2}c_{s_2}S_{p_1r_2}S_{r_1p_2}v^{p_1r_2}_{p_2r_1}S_{q_2s_2}S_{q_2s_2} \tag{122}$$

$$+\frac{1}{2}\sum_{\substack{I_{P_1}\neq I_{P_2}\in G(A)\\ I_{R_1}\neq I_{R_2}\in G(B)}}\sum_{\substack{p_1\in I_{P_1}p_2q_2\in I_{P_2}\\ r_1\in I_{R_1}r_2s_2\in I_{R_2}}} 4c_{p_2}n_{r_1}c_{r_2}n_{p_1}c_{s_2}c_{q_2}S_{p_2r_1}S_{r_2p_1}v^{p_2r_1}_{p_1r_2}S_{s_2q_2}S_{q_2s_2} \tag{123}$$

$$+\sum_{\substack{I_{P_1}\neq I_{P_2}\neq I_{P_3}\in G(A)\\ I_{R_1}\neq I_{R_2}\in G(B)}}\sum_{\substack{p_1\in I_{P_1}p_2\in I_{P_2}p_3\in I_{P_3}\\ r_1\in I_{R_1}r_2s_2\in I_{R_2}}} -4n_{p_1}c_{r_2}c_{s_2}n_{p_2}n_{r_1}n_{p_3}S_{p_1r_2}S_{s_2p_2}v_{p_2s_2}^{p_1r_2}S_{r_1p_3}S_{p_3r_1} \tag{124}$$

$$+\sum_{\substack{I_{P_1}\neq I_{P_2}\in G(A)\\ I_{R_1}\neq I_{R_2}\neq I_{R_3}\in G(B)}}\sum_{\substack{p_1\in I_{P_1}p_2q_2\in I_{P_2}\\ r_1\in I_{R_1}r_2\in I_{R_2}r_3\in I_{R_3}}} -4c_{p_2}n_{r_1}n_{r_2}c_{q_2}n_{r_3}n_{p_1}S_{p_2r_1}S_{r_2q_2}v_{q_2r_2}^{p_2r_1}S_{r_3p_1}S_{p_1r_3} \tag{125}$$

$$+\frac{1}{2}\sum_{\substack{I_{P_1}\neq I_{P_2}\in G(A)\\ I_{R_1}\neq I_{R_2}\in G(B)}}\sum_{\substack{p_1\in I_{P_1}p_2q_2\in I_{P_2}\\ r_1\in I_{R_1}r_2s_2\in I_{R_2}}} 4n_{p_1}n_{r_1}c_{r_2}c_{p_2}c_{q_2}c_{s_2}S_{p_1r_1}S_{r_2p_2}v_{p_2r_2}^{p_1r_1}S_{q_2s_2}S_{q_2s_2} \tag{126}$$

$$+\sum_{\substack{I_{P_1}\neq I_{P_2}\in G(A)\\ I_{R_1}\neq I_{R_2}\neq I_{R_3}\in G(B)}}\sum_{\substack{p_1\in I_{P_1}p_2\in I_{P_2}\\ r_1\in I_{R_1}r_2\in I_{R_2}r_3\in I_{R_3}}} -4n_{p_1}n_{r_1}n_{r_2}n_{r_3}n_{p_2}S_{p_1r_1}v_{r_2r_2}^{p_1r_1}S_{r_3p_2}S_{p_2r_3} \tag{127}$$

$$+\sum_{\substack{I_{P_1}\neq I_{P_2}\neq I_{P_3}\in G(A)\\ I_{R_1}\neq I_{R_2}\neq I_{R_3}\in G(B)}}\sum_{\substack{p_1\in I_{P_1}p_2\in I_{P_2}p_3\in I_{P_3}\\ r_1\in I_{R_1}r_2\in I_{R_2}r_3\in I_{R_3}}} 4n_{p_1}n_{r_1}n_{r_2}n_{p_2}n_{r_3}n_{p_3}S_{p_1r_1}S_{r_2p_2}v_{p_2r_2}^{p_1r_1}S_{r_3p_3}S_{p_3r_3} \tag{128}$$

$$\langle \hat{V}_{ee}\mathcal{P}_4\rangle_{\text{3-loops}} =$$

$$\frac{1}{2}\sum_{\substack{I_{P_1}\neq I_{P_2}\in G(A)\\ I_{R_1}\in G(B)}}\ \sum_{\substack{p_1\in I_{P_1}\, p_2q_2\in I_{P_2}\\ r_1s_1\in I_{R_1}}} 8n_{p_1}c_{r_1}c_{p_2}c_{q_2}c_{s_1}S_{r_1p_2}v^{p_1r_1}_{p_1p_2}S_{q_2s_1}S_{q_2s_1} \tag{129}$$

$$+\frac{1}{2}\sum_{\substack{I_{P_1}\in G(A)\\ I_{R_1}\neq I_{R_2}\in G(B)}}\ \sum_{\substack{p_1q_1\in I_{P_1}\\ r_1\in I_{R_1}\, r_2s_2\in I_{R_2}}} 8c_{p_1}c_{r_2}n_{r_1}c_{s_2}c_{q_1}S_{p_1r_2}v^{p_1r_1}_{r_2r_1}S_{s_2q_1}S_{q_1s_2} \tag{130}$$

$$+\frac{1}{2}\sum_{\substack{I_{P_1}\neq I_{P_2}\neq I_{P_3}\in G(A)\\ I_{R_1}\neq I_{R_2}\in G(B)}}\ \sum_{\substack{p_1\in I_{P_1}\, p_2\in I_{P_2}\, p_3\in I_{P_3}\\ r_1\in I_{R_1}\, r_2s_2\in I_{R_2}}} 8n_{p_1}n_{r_1}c_{r_2}n_{p_2}c_{s_2}n_{p_3}v^{p_1r_1}_{p_1r_1}S_{r_2p_2}S_{p_2s_2}S_{s_2p_3}S_{p_3r_2} \tag{131}$$

$$+\frac{1}{2}\sum_{\substack{I_{P_1}\neq I_{P_2}\in G(A)\\ I_{R_1}\neq I_{R_2}\neq I_{R_3}\in G(B)}}\ \sum_{\substack{p_1\in I_{P_1}\, p_2q_2\in I_{P_2}\\ r_1\in I_{R_1}\, r_2\in I_{R_2}\, r_3\in I_{R_3}}} 8n_{p_1}n_{r_1}n_{r_2}c_{p_2}n_{r_3}c_{q_2}v^{p_1r_1}_{p_1r_1}S_{r_2p_2}S_{p_2r_3}S_{r_3q_2}S_{q_2r_2} \tag{132}$$

$$+\frac{1}{2}\sum_{\substack{I_{P_1}\neq I_{P_2}\neq I_{P_3}\in G(A)\\ I_{R_1}\neq I_{R_2}\neq I_{R_3}\in G(B)}}\ \sum_{\substack{p_1\in I_{P_1}\, p_2\in I_{P_2}\, p_3\in I_{P_3}\\ r_1\in I_{R_1}\, r_2\in I_{R_2}\, r_3\in I_{R_3}}} -8n_{p_1}n_{r_1}n_{r_2}n_{p_2}n_{r_3}n_{p_3}v^{p_1r_1}_{p_1r_1}S_{r_2p_2}S_{p_2r_3}S_{r_3p_3}S_{p_3r_2} \tag{133}$$

$$+\frac{1}{2}\sum_{\substack{I_{P_1}\neq I_{P_2}\in G(A)\\ I_{R_1}\neq I_{R_2}\in G(B)}}\ \sum_{\substack{p_1\in I_{P_1}\, p_2q_2\in I_{P_2}\\ r_1\in I_{R_1}\, r_2s_2\in I_{R_2}}} -8n_{p_1}c_{r_2}c_{p_2}n_{r_1}c_{q_2}c_{s_2}S_{r_2p_2}S_{p_2r_1}v^{p_1r_2}_{p_1r_1}S_{q_2s_2}S_{q_2s_2} \tag{134}$$

$$+\frac{1}{2}\sum_{\substack{I_{P_1}\neq I_{P_2}\in G(A)\\ I_{R_1}\neq I_{R_2}\in G(B)}}\ \sum_{\substack{p_1\in I_{P_1}\, p_2q_2\in I_{P_2}\\ r_1\in I_{R_1}\, r_2s_2\in I_{R_2}}} -8n_{p_1}n_{r_1}c_{p_2}c_{r_2}c_{s_2}c_{q_2}S_{r_1p_2}S_{p_2r_2}v^{p_1r_1}_{p_1r_2}S_{s_2q_2}S_{q_2s_2} \tag{135}$$

$$+\sum_{\substack{I_{P_1}\neq I_{P_2}\neq I_{P_3}\in G(A)\\ I_{R_1}\neq I_{R_2}\in G(B)}}\ \sum_{\substack{p_1\in I_{P_1}\, p_2\in I_{P_2}\, p_3\in I_{P_3}\\ r_1\in I_{R_1}\, r_2s_2\in I_{R_2}}} 8n_{p_1}c_{r_2}n_{p_2}c_{s_2}n_{r_1}n_{p_3}S_{r_2p_2}S_{p_2s_2}v^{p_1r_2}_{p_1s_2}S_{r_1p_3}S_{p_3r_1} \tag{136}$$

$$+\sum_{\substack{I_{P_1}\neq I_{P_2}\neq I_{P_3}\in G(A)\\ I_{R_1}\neq I_{R_2}\in G(B)}}\ \sum_{\substack{p_1\in I_{P_1}\, p_2\in I_{P_2}\, p_3\in I_{P_3}\\ r_1\in I_{R_1}\, r_2\in I_{R_2}}} 8n_{p_1}n_{r_1}n_{p_2}n_{r_2}n_{p_3}S_{r_1p_2}v^{p_1r_1}_{p_1p_2}S_{r_2p_3}S_{p_3r_2} \tag{137}$$

$$+\sum_{\substack{I_{P_1}\neq I_{P_2}\neq I_{P_3}\in G(A)\\ I_{R_1}\neq I_{R_2}\neq I_{R_3}\in G(B)}}\ \sum_{\substack{p_1\in I_{P_1}\, p_2\in I_{P_2}\, p_3\in I_{P_3}\\ r_1\in I_{R_1}\, r_2\in I_{R_2}\, r_3\in I_{R_3}}} -8n_{p_1}n_{r_1}n_{p_2}n_{r_2}n_{r_3}n_{p_3}S_{r_1p_2}S_{p_2r_2}v^{p_1r_1}_{p_1r_2}S_{r_3p_3}S_{p_3r_3} \tag{138}$$

$$+\frac{1}{2}\sum_{\substack{I_{P_1}\neq I_{P_2}\in G(A)\\ I_{R_1}\neq I_{R_2}\in G(B)}}\ \sum_{\substack{p_1\in I_{P_1}\, p_2q_2\in I_{P_2}\\ r_1\in I_{R_1}\, r_2s_2\in I_{R_2}}} -8n_{p_1}c_{r_2}c_{p_2}n_{r_1}c_{q_2}c_{s_2}S_{p_1r_2}S_{r_2p_2}v^{p_1r_1}_{p_2r_1}S_{q_2s_2}S_{q_2s_2} \tag{139}$$

$$+\frac{1}{2}\sum_{\substack{I_{P_1}\neq I_{P_2}\in G(A)\\ I_{R_1}\neq I_{R_2}\in G(B)}}\ \sum_{\substack{p_1\in I_{P_1}\, p_2q_2\in I_{P_2}\\ r_1\in I_{R_1}\, r_2s_2\in I_{R_2}}} -8c_{p_2}c_{r_2}n_{p_1}n_{r_1}c_{s_2}c_{q_2}S_{p_2r_2}S_{r_2p_1}v^{p_2r_1}_{p_1r_1}S_{s_2q_2}S_{q_2s_2} \tag{140}$$

$$+ \sum_{\substack{I_{P_1} \neq I_{P_2} \in G(A) \\ I_{R_1} \neq I_{R_2} \neq I_{R_3} \in G(B)}} \sum_{\substack{p_1 \in I_{P_1} p_2 \in I_{P_2} \\ r_1 \in I_{R_1} r_2 \in I_{R_2} r_3 \in I_{R_3}}} 8 n_{p_1} n_{r_1} n_{r_2} n_{r_3} n_{p_2} S_{p_1 r_1} v^{p_1 r_2}_{r_1 r_2} S_{r_3 p_2} S_{p_2 r_3} \tag{141}$$

$$+ \sum_{\substack{I_{P_1} \neq I_{P_2} \in G(A) \\ I_{R_1} \neq I_{R_2} \neq I_{R_3} \in G(B)}} \sum_{\substack{p_1 \in I_{P_1} p_2 q_2 \in I_{P_2} \\ r_1 \in I_{R_1} r_2 \in I_{R_2} r_3 \in I_{R_3}}} 8 c_{p_2} n_{r_1} c_{q_2} n_{r_2} n_{r_3} n_{p_1} S_{p_2 r_1} S_{r_1 q_2} v^{p_2 r_2}_{q_2 r_2} S_{r_3 p_1} S_{p_1 r_3} \tag{142}$$

$$+ \sum_{\substack{I_{P_1} \neq I_{P_2} \neq I_{P_3} \in G(A) \\ I_{R_1} \neq I_{R_2} \neq I_{R_3} \in G(B)}} \sum_{\substack{p_1 \in I_{P_1} p_2 \in I_{P_2} p_3 \in I_{P_3} \\ r_1 \in I_{R_1} r_2 \in I_{R_2} r_3 \in I_{R_3}}} - 8 n_{p_1} n_{r_1} n_{p_2} n_{r_2} n_{r_3} n_{p_3} S_{p_1 r_1} S_{r_1 p_2} v^{p_1 r_2}_{p_2 r_2} S_{r_3 p_3} S_{p_3 r_3} \tag{143}$$

$$\langle \hat{V}_{ee}\mathcal{P}_4\rangle_{\text{4-loops}} = \tag{144}$$

$$\frac{1}{2}\cdot\frac{1}{2}\sum_{\substack{I_{P_1}\neq I_{P_2}\in G(A)\\ I_{R_1}\neq I_{R_2}\in G(B)}}\sum_{\substack{p_1q_1\in I_{P_1}p_2\in I_{P_2}\\ r_1s_1\in I_{R_1}r_2\in I_{R_2}}} 16c_{r_1}c_{p_1}c_{q_1}c_{s_1}S_{r_1p_1}S_{q_1s_1}S_{p_1r_1}S_{q_1s_1}n_{p_2}n_{r_2}v^{p_2r_2}_{p_2r_2} \tag{145}$$

$$+\frac{1}{2}\sum_{\substack{I_{P_1}\neq I_{P_2}\neq I_{P_3}\in G(A)\\ I_{R_1}\neq I_{R_2}\neq I_{R_3}\in G(B)}}\sum_{\substack{p_1\in I_{P_1}p_2\in I_{P_2}p_3\in I_{P_3}\\ r_1\in I_{R_1}r_2\in I_{R_2}r_3\in I_{R_3}}} 16n_{r_1}n_{p_1}n_{r_2}n_{p_2}S_{r_1p_1}S_{r_2p_2}S_{p_1r_1}S_{p_2r_2}n_{p_3}n_{r_3}v^{p_3r_3}_{p_3r_3} \tag{146}$$

B. $\langle \hat{V}_A \mathcal{P}_4 \rangle$

$$\langle \hat{V}_A \mathcal{P}_4 \rangle_{\text{1-loop}} =$$

$$\sum_{\substack{I_{P_1} \neq I_{P_2} \in G(A) \\ I_{R_1} \in G(B)}} \sum_{\substack{p_1 \in I_{P_1} p_2 \in I_{P_2} \\ r_1 s_1 \in I_{R_1}}} 2c_{r_1} n_{p_1} c_{s_1} n_{p_2} S_{r_1 p_1} S_{p_1 s_1} S_{s_1 p_2} (v_A)_{r_1 p_2} \tag{147}$$

$$+ \sum_{\substack{I_{P_1} \in G(A) \\ I_{R_1} \neq I_{R_2} \in G(B)}} \sum_{\substack{p_1 q_1 \in I_{P_1} \\ r_1 \in I_{R_1} r_2 s_2 \in I_{R_2}}} 2c_{r_2} c_{p_1} n_{r_1} c_{q_1} c_{s_2} S_{r_2 p_1} S_{p_1 r_1} S_{r_1 q_1} S_{q_1 s_2} (v_A)_{r_2 s_2} \tag{148}$$

$$+ \sum_{\substack{I_{P_1} \in G(A) \\ I_{R_1} \neq I_{R_2} \in G(B)}} \sum_{\substack{p_1 q_1 \in I_{P_1} \\ r_1 \in I_{R_1} r_2 \in I_{R_2}}} 2n_{r_1} c_{p_1} n_{r_2} c_{q_1} S_{r_1 p_1} S_{p_1 r_2} S_{r_2 q_1} (v_A)_{r_1 q_1} \tag{149}$$

$$+ \sum_{\substack{I_{P_1} \neq I_{P_2} \in G(A) \\ I_{R_1} \neq I_{R_2} \in G(B)}} \sum_{\substack{p_1 \in I_{P_1} p_2 \in I_{P_2} \\ r_1 \in I_{R_1} r_2 s_2 \in I_{R_2}}} -2c_{r_2} n_{p_1} c_{s_2} n_{p_2} n_{r_1} S_{r_2 p_1} S_{p_1 s_2} S_{s_2 p_2} S_{p_2 r_1} (v_A)_{r_2 r_1} \tag{150}$$

$$+ \sum_{\substack{I_{P_1} \neq I_{P_2} \in G(A) \\ I_{R_1} \neq I_{R_2} \in G(B)}} \sum_{\substack{p_1 \in I_{P_1} p_2 \in I_{P_2} \\ r_1 \in I_{R_1} r_2 s_2 \in I_{R_2}}} -2c_{r_2} n_{p_1} n_{r_1} n_{p_2} c_{s_2} S_{r_2 p_1} S_{p_1 r_1} S_{r_1 p_2} S_{p_2 s_2} (v_A)_{r_2 s_2} \tag{151}$$

$$+ \sum_{\substack{I_{P_1} \neq I_{P_2} \in G(A) \\ I_{R_1} \neq I_{R_2} \in G(B)}} \sum_{\substack{p_1 \in I_{P_1} p_2 \in I_{P_2} \\ r_1 \in I_{R_1} r_2 s_2 \in I_{R_2}}} -2n_{r_1} n_{p_1} c_{r_2} n_{p_2} c_{s_2} S_{r_1 p_1} S_{p_1 r_2} S_{r_2 p_2} S_{p_2 s_2} (v_A)_{r_1 s_2} \tag{152}$$

$$+ \sum_{\substack{I_{P_1} \neq I_{P_2} \in G(A) \\ I_{R_1} \neq I_{R_2} \in G(B)}} \sum_{\substack{p_1 \in I_{P_1} p_2 \in I_{P_2} \\ r_1 \in I_{R_1} r_2 \in I_{R_2}}} -2n_{r_1} n_{p_1} n_{r_2} n_{p_2} S_{r_1 p_1} S_{p_1 r_2} S_{r_2 p_2} (v_A)_{r_1 p_2} \tag{153}$$

$$+ \sum_{\substack{I_{P_1} \in G(A) \\ I_{R_1} \neq I_{R_2} \neq I_{R_3} \in G(B)}} \sum_{\substack{p_1 q_1 \in I_{P_1} \\ r_1 \in I_{R_1} r_2 \in I_{R_2} r_3 \in I_{R_3}}} -2n_{r_1} c_{p_1} n_{r_2} c_{q_1} n_{r_3} S_{r_1 p_1} S_{p_1 r_2} S_{r_2 q_1} S_{q_1 r_3} (v_A)_{r_1 r_3} \tag{154}$$

$$+ \sum_{\substack{I_{P_1} \neq I_{P_2} \in G(A) \\ I_{R_1} \neq I_{R_2} \neq I_{R_3} \in G(B)}} \sum_{\substack{p_1 \in I_{P_1} p_2 \in I_{P_2} \\ r_1 \in I_{R_1} r_2 \in I_{R_2} r_3 \in I_{R_3}}} 2n_{r_1} n_{p_1} n_{r_2} n_{p_2} n_{r_3} S_{r_1 p_1} S_{p_1 r_2} S_{r_2 p_2} S_{p_2 r_3} (v_A)_{r_1 r_3} \tag{155}$$

$$\langle \hat{V}_A \mathcal{P}_4 \rangle_{\text{2-loops}}$$

$$= \frac{1}{2} \sum_{\substack{I_{P_1} \in G(A) \\ I_{R_1} \in G(B)}} \sum_{\substack{p_1 q_1 \in I_{P_1} \\ r_1 s_1 \in I_{R_1}}} 4 c_{r_1} c_{p_1} c_{q_1} c_{s_1} S_{r_1 p_1} S_{q_1 s_1} (v_A)_{r_1 p_1} S_{q_1 s_1} \tag{156}$$

$$+ \frac{1}{2} \sum_{\substack{I_{P_1} \neq I_{P_2} \in G(A) \\ I_{R_1} \neq I_{R_2} \in G(B)}} \sum_{\substack{p_1 \in I_{P_1} p_2 \in I_{P_2} \\ r_1 \in I_{R_1} r_2 s_2 \in I_{R_2}}} 4 n_{r_1} c_{r_2} n_{p_1} c_{s_2} n_{p_2} S_{r_2 p_1} S_{p_1 s_2} S_{s_2 p_2} (v_A)_{r_1 r_1} S_{p_2 r_2} \tag{157}$$

$$+ \frac{1}{2} \sum_{\substack{I_{P_1} \in G(A) \\ I_{R_1} \neq I_{R_2} \neq I_{R_3} \in G(B)}} \sum_{\substack{p_1 q_1 \in I_{P_1} \\ r_1 \in I_{R_1} r_2 \in I_{R_2} r_3 \in I_{R_3}}} 4 n_{r_1} n_{r_2} c_{p_1} n_{r_3} c_{q_1} S_{r_2 p_1} S_{p_1 r_3} S_{r_3 q_1} (v_A)_{r_1 r_1} S_{q_1 r_2} \tag{158}$$

$$+ \frac{1}{2} \sum_{\substack{I_{P_1} \neq I_{P_2} \in G(A) \\ I_{R_1} \neq I_{R_2} \neq I_{R_3} \in G(B)}} \sum_{\substack{p_1 \in I_{P_1} p_2 \in I_{P_2} \\ r_1 \in I_{R_1} r_2 \in I_{R_2} r_3 \in I_{R_3}}} -4 n_{r_1} n_{r_2} n_{p_1} n_{r_3} n_{p_2} S_{r_2 p_1} S_{p_1 r_3} S_{r_3 p_2} (v_A)_{r_1 r_1} S_{p_2 r_2} \tag{159}$$

$$+ \frac{1}{2} \sum_{\substack{I_{P_1} \in G(A) \\ I_{R_1} \neq I_{R_2} \in G(B)}} \sum_{\substack{p_1 q_1 \in I_{P_1} \\ r_1 \in I_{R_1} r_2 s_2 \in I_{R_2}}} -4 c_{r_2} c_{p_1} n_{r_1} c_{q_1} c_{s_2} S_{r_2 p_1} S_{p_1 r_1} S_{q_1 s_2} (v_A)_{r_2 r_1} S_{q_1 s_2} \tag{160}$$

$$+ \frac{1}{2} \sum_{\substack{I_{P_1} \in G(A) \\ I_{R_1} \neq I_{R_2} \in G(B)}} \sum_{\substack{p_1 q_1 \in I_{P_1} \\ r_1 \in I_{R_1} r_2 s_2 \in I_{R_2}}} -4 n_{r_1} c_{p_1} c_{r_2} c_{s_2} c_{q_1} S_{r_1 p_1} S_{p_1 r_2} S_{s_2 q_1} (v_A)_{r_1 r_2} S_{q_1 s_2} \tag{161}$$

$$+ \sum_{\substack{I_{P_1} \neq I_{P_2} \in G(A) \\ I_{R_1} \neq I_{R_2} \in G(B)}} \sum_{\substack{p_1 \in I_{P_1} p_2 \in I_{P_2} \\ r_1 \in I_{R_1} r_2 s_2 \in I_{R_2}}} 4 c_{r_2} n_{p_1} c_{s_2} n_{r_1} n_{p_2} S_{r_2 p_1} S_{p_1 s_2} S_{r_1 p_2} (v_A)_{r_2 s_2} S_{p_2 r_1} \tag{162}$$

$$+ \sum_{\substack{I_{P_1} \neq I_{P_2} \in G(A) \\ I_{R_1} \neq I_{R_2} \in G(B)}} \sum_{\substack{p_1 \in I_{P_1} p_2 \in I_{P_2} \\ r_1 \in I_{R_1} r_2 \in I_{R_2}}} 4 n_{r_1} n_{p_1} n_{r_2} n_{p_2} S_{r_1 p_1} S_{r_2 p_2} (v_A)_{r_1 p_1} S_{p_2 r_2} \tag{163}$$

$$+ \sum_{\substack{I_{P_1} \neq I_{P_2} \in G(A) \\ I_{R_1} \neq I_{R_2} \neq I_{R_3} \in G(B)}} \sum_{\substack{p_1 \in I_{P_1} p_2 \in I_{P_2} \\ r_1 \in I_{R_1} r_2 \in I_{R_2} r_3 \in I_{R_3}}} -4 n_{r_1} n_{p_1} n_{r_2} n_{r_3} n_{p_2} S_{r_1 p_1} S_{p_1 r_2} S_{r_3 p_2} (v_A)_{r_1 r_2} S_{p_2 r_3} \tag{164}$$

$$\langle \hat{V}_A \mathcal{P}_4 \rangle_{\text{3-loops}}$$

$$= \frac{1}{2} \cdot \frac{1}{2} \sum_{\substack{I_{P_1} \in G(A) \\ I_{R_1} \neq I_{R_2} \in G(B)}} \sum_{\substack{p_1 q_1 \in I_{P_1} \\ r_1 s_1 \in I_{R_1} r_2 \in I_{R_2}}} 8 c_{r_1} c_{p_1} c_{q_1} c_{s_1} S_{r_1 p_1} S_{q_1 s_1} S_{p_1 r_1} S_{q_1 s_1} n_{r_2} (v_A)_{r_2 r_2} \tag{165}$$

$$+ \frac{1}{2} \sum_{\substack{I_{P_1} \neq I_{P_2} \in G(A) \\ I_{R_1} \neq I_{R_2} \neq I_{R_3} \in G(B)}} \sum_{\substack{p_1 \in I_{P_1} p_2 \in I_{P_2} \\ r_1 \in I_{R_1} r_2 \in I_{R_2} r_3 \in I_{R_3}}} 8 n_{r_1} n_{p_1} n_{r_2} n_{p_2} S_{r_1 p_1} S_{r_2 p_2} S_{p_1 r_1} S_{p_2 r_2} n_{r_3} (v_A)_{r_3 r_3} \tag{166}$$

C. $\langle \hat{V}_B \mathcal{P}_4 \rangle$

$$\langle \hat{V}_B \mathcal{P}_4 \rangle_{\text{1-loop}} =$$

$$+ \sum_{\substack{I_{P_1} \in G(A) \\ I_{R_1} \in G(B)}} \sum_{\substack{p_1 q_1 \in I_{P_1} \\ r_1 s_1 \in I_{R_1}}} 4 c_{p_1} c_{r_1} c_{s_1} c_{q_1} S_{p_1 r_1} S_{s_1 q_1} (v_B)_{p_1 r_1} S_{q_1 s_1} \tag{167}$$

$$+ \sum_{\substack{I_{P_1} \neq I_{P_2} \neq I_{P_3} \in G(A) \\ I_{R_1} \in G(B)}} \sum_{\substack{p_1 \in I_{P_1} p_2 \in I_{P_2} p_3 \in I_{P_3} \\ r_1 s_1 \in I_{R_1}}} 4 n_{p_1} c_{r_1} n_{p_2} c_{s_1} n_{p_3} S_{r_1 p_2} S_{p_2 s_1} S_{s_1 p_3} (v_B)_{p_1 p_1} S_{p_3 r_1} \tag{168}$$

$$+ \sum_{\substack{I_{P_1} \neq I_{P_2} \in G(A) \\ I_{R_1} \neq I_{R_2} \in G(B)}} \sum_{\substack{p_1 \in I_{P_1} p_2 q_2 \in I_{P_2} \\ r_1 \in I_{R_1} r_2 \in I_{R_2}}} 4 n_{p_1} n_{r_1} c_{p_2} n_{r_2} c_{q_2} S_{r_1 p_2} S_{p_2 r_2} S_{r_2 q_2} (v_B)_{p_1 p_1} S_{q_2 r_1} \tag{169}$$

$$+ \sum_{\substack{I_{P_1} \neq I_{P_2} \neq I_{P_3} \in G(A) \\ I_{R_1} \neq I_{R_2} \in G(B)}} \sum_{\substack{p_1 \in I_{P_1} p_2 \in I_{P_2} p_3 \in I_{P_3} \\ r_1 \in I_{R_1} r_2 \in I_{R_2}}} -4 n_{p_1} n_{r_1} n_{p_2} n_{r_2} n_{p_3} S_{r_1 p_2} S_{p_2 r_2} S_{r_2 p_3} (v_B)_{p_1 p_1} S_{p_3 r_1} \tag{170}$$

$$+ \sum_{\substack{I_{P_1} \neq I_{P_2} \in G(A) \\ I_{R_1} \in G(B)}} \sum_{\substack{p_1 \in I_{P_1} p_2 q_2 \in I_{P_2} \\ r_1 s_1 \in I_{R_1}}} -4 n_{p_1} c_{r_1} c_{p_2} c_{q_2} c_{s_1} S_{p_1 r_1} S_{r_1 p_2} S_{q_2 s_1} (v_B)_{p_1 p_2} S_{q_2 s_1} \tag{171}$$

$$+ \sum_{\substack{I_{P_1} \neq I_{P_2} \in G(A) \\ I_{R_1} \in G(B)}} \sum_{\substack{p_1 \in I_{P_1} p_2 q_2 \in I_{P_2} \\ r_1 s_1 \in I_{R_1}}} -4 c_{p_2} c_{r_1} n_{p_1} c_{s_1} c_{q_2} S_{p_2 r_1} S_{r_1 p_1} S_{s_1 q_2} (v_B)_{p_2 p_1} S_{q_2 s_1} \tag{172}$$

$$+ \sum_{\substack{I_{P_1} \neq I_{P_2} \in G(A) \\ I_{R_1} \neq I_{R_2} \in G(B)}} \sum_{\substack{p_1 \in I_{P_1} p_2 \in I_{P_2} \\ r_1 \in I_{R_1} r_2 \in I_{R_2}}} 4 n_{p_1} n_{r_1} n_{r_2} n_{p_2} S_{p_1 r_1} S_{r_2 p_2} (v_B)_{p_1 r_1} S_{p_2 r_2} \tag{173}$$

$$+ \sum_{\substack{I_{P_1} \neq I_{P_2} \in G(A) \\ I_{R_1} \neq I_{R_2} \in G(B)}} \sum_{\substack{p_1 \in I_{P_1} p_2 q_2 \in I_{P_2} \\ r_1 \in I_{R_1} r_2 \in I_{R_2}}} 4 c_{p_2} n_{r_1} c_{q_2} n_{r_2} n_{p_1} S_{p_2 r_1} S_{r_1 q_2} S_{r_2 p_1} (v_B)_{p_2 q_2} S_{p_1 r_2} \tag{174}$$

$$+ \sum_{\substack{I_{P_1} \neq I_{P_2} \neq I_{P_3} \in G(A) \\ I_{R_1} \neq I_{R_2} \in G(B)}} \sum_{\substack{p_1 \in I_{P_1} p_2 \in I_{P_2} p_3 \in I_{P_3} \\ r_1 \in I_{R_1} r_2 \in I_{R_2}}} -4 n_{p_1} n_{r_1} n_{p_2} n_{r_2} n_{p_3} S_{p_1 r_1} S_{r_1 p_2} S_{r_2 p_3} (v_B)_{p_1 p_2} S_{p_3 r_2} \tag{175}$$

$$\langle \hat{V}_B \mathcal{P}_4 \rangle_{\text{2-loops}} =$$

$$\frac{1}{2} \sum_{\substack{I_{P_1} \in G(A) \\ I_{R_1} \in G(B)}} \sum_{\substack{p_1 q_1 \in I_{P_1} \\ r_1 s_1 \in I_{R_1}}} 4 c_{p_1} c_{r_1} c_{s_1} c_{q_1} S_{p_1 r_1} S_{s_1 q_1} (v_B)_{p_1 r_1} S_{q_1 s_1} \tag{176}$$

$$+ \frac{1}{2} \sum_{\substack{I_{P_1} \neq I_{P_2} \neq I_{P_3} \in G(A) \\ I_{R_1} \in G(B)}} \sum_{\substack{p_1 \in I_{P_1} p_2 \in I_{P_2} p_3 \in I_{P_3} \\ r_1 s_1 \in I_{R_1}}} 4 n_{p_1} c_{r_1} n_{p_2} c_{s_1} n_{p_3} S_{r_1 p_2} S_{p_2 s_1} S_{s_1 p_3} (v_B)_{p_1 p_1} S_{p_3 r_1} \tag{177}$$

$$+ \frac{1}{2} \sum_{\substack{I_{P_1} \neq I_{P_2} \in G(A) \\ I_{R_1} \neq I_{R_2} \in G(B)}} \sum_{\substack{p_1 \in I_{P_1} p_2 q_2 \in I_{P_2} \\ r_1 \in I_{R_1} r_2 \in I_{R_2}}} 4 n_{p_1} n_{r_1} c_{p_2} n_{r_2} c_{q_2} S_{r_1 p_2} S_{p_2 r_2} S_{r_2 q_2} (v_B)_{p_1 p_1} S_{q_2 r_1} \tag{178}$$

$$+ \frac{1}{2} \sum_{\substack{I_{P_1} \neq I_{P_2} \neq I_{P_3} \in G(A) \\ I_{R_1} \neq I_{R_2} \in G(B)}} \sum_{\substack{p_1 \in I_{P_1} p_2 \in I_{P_2} p_3 \in I_{P_3} \\ r_1 \in I_{R_1} r_2 \in I_{R_2}}} -4 n_{p_1} n_{r_1} n_{p_2} n_{r_2} n_{p_3} S_{r_1 p_2} S_{p_2 r_2} S_{r_2 p_3} (v_B)_{p_1 p_1} S_{p_3 r_1} \tag{179}$$

$$+ \frac{1}{2} \sum_{\substack{I_{P_1} \neq I_{P_2} \in G(A) \\ I_{R_1} \in G(B)}} \sum_{\substack{p_1 \in I_{P_1} p_2 q_2 \in I_{P_2} \\ r_1 s_1 \in I_{R_1}}} -4 n_{p_1} c_{r_1} c_{p_2} c_{q_2} c_{s_1} S_{p_1 r_1} S_{r_1 p_2} S_{q_2 s_1} (v_B)_{p_1 p_2} S_{q_2 s_1} \tag{180}$$

$$+ \frac{1}{2} \sum_{\substack{I_{P_1} \neq I_{P_2} \in G(A) \\ I_{R_1} \in G(B)}} \sum_{\substack{p_1 \in I_{P_1} p_2 q_2 \in I_{P_2} \\ r_1 s_1 \in I_{R_1}}} -4 c_{p_2} c_{r_1} n_{p_1} c_{s_1} c_{q_2} S_{p_2 r_1} S_{r_1 p_1} S_{s_1 q_2} (v_B)_{p_2 p_1} S_{q_2 s_1} \tag{181}$$

$$+ \sum_{\substack{I_{P_1} \neq I_{P_2} \in G(A) \\ I_{R_1} \neq I_{R_2} \in G(B)}} \sum_{\substack{p_1 \in I_{P_1} p_2 \in I_{P_2} \\ r_1 \in I_{R_1} r_2 \in I_{R_2}}} 4 n_{p_1} n_{r_1} n_{r_2} n_{p_2} S_{p_1 r_1} S_{r_2 p_2} (v_B)_{p_1 r_1} S_{p_2 r_2} \tag{182}$$

$$+ \sum_{\substack{I_{P_1} \neq I_{P_2} \in G(A) \\ I_{R_1} \neq I_{R_2} \in G(B)}} \sum_{\substack{p_1 \in I_{P_1} p_2 q_2 \in I_{P_2} \\ r_1 \in I_{R_1} r_2 \in I_{R_2}}} 4 c_{p_2} n_{r_1} c_{q_2} n_{r_2} n_{p_1} S_{p_2 r_1} S_{r_1 q_2} S_{r_2 p_1} (v_B)_{p_2 q_2} S_{p_1 r_2} \tag{183}$$

$$+ \sum_{\substack{I_{P_1} \neq I_{P_2} \neq I_{P_3} \in G(A) \\ I_{R_1} \neq I_{R_2} \in G(B)}} \sum_{\substack{p_1 \in I_{P_1} p_2 \in I_{P_2} p_3 \in I_{P_3} \\ r_1 \in I_{R_1} r_2 \in I_{R_2}}} -4 n_{p_1} n_{r_1} n_{p_2} n_{r_2} n_{p_3} S_{p_1 r_1} S_{r_1 p_2} S_{r_2 p_3} (v_B)_{p_1 p_2} S_{p_3 r_2} \tag{184}$$

$$\langle \hat{V}_B \mathcal{P}_4 \rangle_{\text{3-loops}} =$$

$$\frac{1}{2} \cdot \frac{1}{2} \sum_{\substack{I_{P_1} \neq I_{P_2} \in G(A) \\ I_{R_1} \in G(B)}} \sum_{\substack{p_1 q_1 \in I_{P_1} p_2 \in I_{P_2} \\ r_1 s_1 \in I_{R_1}}} 8 c_{r_1} c_{p_1} c_{q_1} c_{s_1} S_{r_1 p_1} S_{q_1 s_1} S_{p_1 r_1} S_{q_1 s_1} n_{p_2} (v_B)_{p_2 p_2} \tag{185}$$

$$+ \frac{1}{2} \sum_{\substack{I_{P_1} \neq I_{P_2} \neq I_{P_3} \in G(A) \\ I_{R_1} \neq I_{R_2} \in G(B)}} \sum_{\substack{p_1 \in I_{P_1} p_2 \in I_{P_2} p_3 \in I_{P_3} \\ r_1 \in I_{R_1} r_2 \in I_{R_2}}} 8 n_{r_1} n_{p_1} n_{r_2} n_{p_2} S_{r_1 p_1} S_{r_2 p_2} S_{p_1 r_1} S_{p_2 r_2} n_{p_3} (v_B)_{p_3 p_3} \tag{186}$$

D. $\langle \mathcal{P}_4 \rangle$

$$\langle \mathcal{P}_4 \rangle_{\text{1-loop}} =$$

$$\frac{1}{2} \sum_{\substack{I_{P_1} \neq I_{P_2} \in G(A) \\ I_{R_1} \in G(B)}} \sum_{p_1 \in I_{P_1} p_2 \in I_{P_2} r_1 s_1 \in I_{R_1}} 2 c_{r_1} n_{p_1} c_{s_1} n_{p_2} S_{p_1 r_1} S_{p_1 s_1} S_{p_2 s_1} S_{p_2 r_1} \tag{187}$$

$$+ \frac{1}{2} \sum_{\substack{I_{P_1} \in G(A) \\ I_{R_1} \neq I_{R_2} \in G(B)}} \sum_{p_1 q_1 \in I_{P_1} r_1 \in I_{R_1} r_2 \in I_{R_2}} 2 n_{r_1} c_{p_1} n_{r_2} c_{q_1} S_{p_1 r_1} S_{p_1 r_2} S_{q_1 r_2} S_{q_1 r_1} \tag{188}$$

$$+ \frac{1}{2} \sum_{\substack{I_{P_1} \neq I_{P_2} \in G(A) \\ I_{R_1} \neq I_{R_2} \in G(B)}} \sum_{p_1 \in I_{P_1} p_2 \in I_{P_2} r_1 \in I_{R_1} r_2 \in I_{R_2}} -2 n_{r_1} n_{p_1} n_{r_2} n_{p_2} S_{p_1 r_1} S_{p_1 r_2} S_{p_2 r_2} S_{p_2 r_1} \tag{189}$$

$$\langle \mathcal{P}_4 \rangle_{\text{2-loops}} =$$

$$\frac{1}{2} \cdot \frac{1}{2} \sum_{\substack{I_{P_1} \in G(A) \\ I_{R_1} \in G(B)}} \sum_{\substack{p_1 q_1 \in I_{P_1} \\ r_1 s_1 \in I_{R_1}}} 4 c_{r_1} c_{p_1} c_{q_1} c_{s_1} S_{r_1 p_1} S_{q_1 s_1} S_{p_1 r_1} S_{q_1 s_1} \tag{190}$$

$$+ \frac{1}{2} \sum_{\substack{I_{P_1} \neq I_{P_2} \in G(A) \\ I_{R_1} \neq I_{R_2} \in G(B)}} \sum_{\substack{p_1 \in I_{P_1} p_2 \in I_{P_2} \\ r_1 \in I_{R_1} r_2 \in I_{R_2}}} 4 n_{r_1} n_{p_1} n_{r_2} n_{p_2} S_{r_1 p_1} S_{r_2 p_2} S_{p_1 r_1} S_{p_2 r_2} \tag{191}$$

$\langle V_{ee} \rangle$

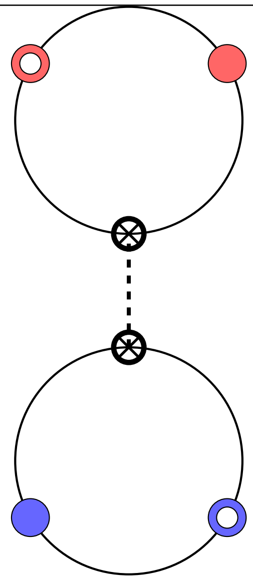

$$\sum_{\substack{I_P \in G(A) \\ I_R \in G(B)}} \sum_{\substack{p \in I_P \\ r \in I_R}} 4 n_p n_r v_{pr}^{pr}$$

$\langle V_A \rangle$

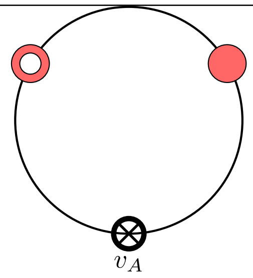

$$\sum_{I_R \in G(B)} \sum_{r \in I_R} 2 n_r v_{rr}^{A}$$

$\langle V_B \rangle$

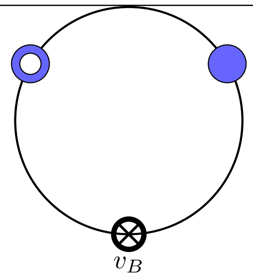

$$\sum_{I_P \in G(A)} \sum_{p \in I_P} 2 n_p v_{pp}^{B}$$

$\langle V_{ee} \mathcal{P}_2 \rangle$

(1)	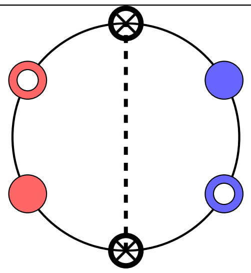	$\sum_{\substack{I_{P_1}\in G(A)\\ I_{R_1}\in G(B)}} \sum_{\substack{p_1\in I_{P_1}\\ r_1\in I_{R_1}}} -2 n_{p_1} n_{r_1} v^{p_1 p_1}_{r_1 r_1}$
(2)	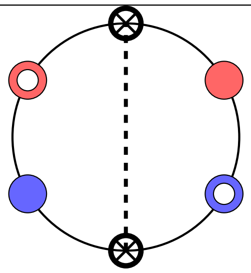	$\sum_{\substack{I_{P_1}\in G(A)\\ I_{R_1}\in G(B)}} \sum_{\substack{p_1 q_1\in I_{P_1}\\ r_1 s_1\in I_{R_1}}} -2 c_{p_1} c_{r_1} c_{s_1} c_{q_1} S_{p_1 r_1} S_{s_1 q_1} v^{p_1 r_1}_{q_1 s_1}$
(3)	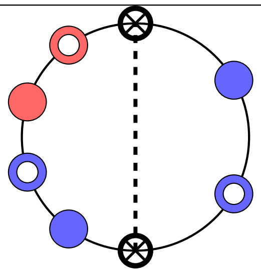	$\sum_{\substack{I_{P_1}\neq I_{P_2}\in G(A)\\ I_{R_1}\in G(B)}} \sum_{\substack{p_1\in I_{P_1}\, p_2\in I_{P_2}\\ r_1\in I_{R_1}}} 2 n_{p_1} n_{r_1} n_{p_2} S_{r_1 p_2} v^{p_1 p_1}_{p_2 r_1}$
(4)	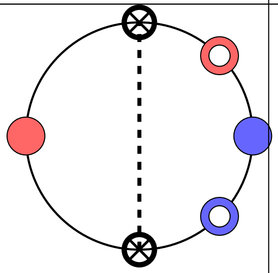	$\sum_{\substack{I_{P_1}\in G(A)\\ I_{R_1}\in G(B)}} \sum_{\substack{p_1\in I_{P_1}\\ r_1 s_1\in I_{R_1}}} -2 n_{p_1} c_{r_1} c_{s_1} S_{p_1 r_1} v^{p_1 r_1}_{s_1 s_1}$
(5)	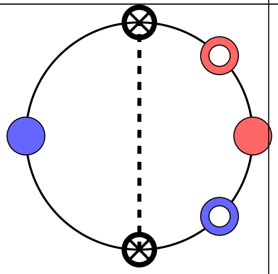	$\sum_{\substack{I_{P_1}\in G(A)\\ I_{R_1}\in G(B)}} \sum_{\substack{p_1 q_1\in I_{P_1}\\ r_1\in I_{R_1}}} -2 c_{p_1} n_{r_1} c_{q_1} S_{p_1 r_1} v^{p_1 r_1}_{q_1 q_1}$
(6)	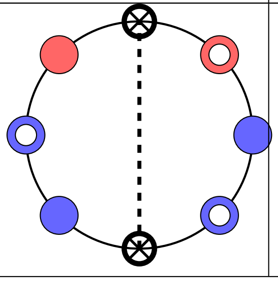	$\sum_{\substack{I_{P_1}\neq I_{P_2}\in G(A)\\ I_{R_1}\in G(B)}} \sum_{\substack{p_1\in I_{P_1}\, p_2\in I_{P_2}\\ r_1 s_1\in I_{R_1}}} 2 n_{p_1} c_{r_1} c_{s_1} n_{p_2} S_{p_1 r_1} S_{s_1 p_2} v^{p_1 r_1}_{p_2 s_1}$

(7)	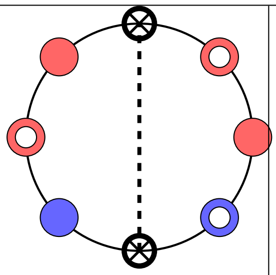	$\sum_{\substack{I_{P_1}\in G(A)\\ I_{R_1}\neq I_{R_2}\in G(B)}} \sum_{\substack{p_1 q_1\in I_{P_1}\\ r_1\in I_{R_1} r_2\in I_{R_2}}} 2c_{p_1} n_{r_1} n_{r_2} c_{q_1} S_{p_1 r_1} S_{r_2 q_1} v^{p_1 r_1}_{q_1 r_2}$
(8)	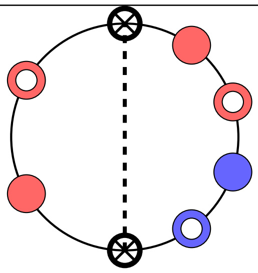	$\sum_{\substack{I_{P_1}\in G(A)\\ I_{R_1}\neq I_{R_2}\in G(B)}} \sum_{\substack{p_1\in I_{P_1}\\ r_1\in I_{R_1} r_2\in I_{R_2}}} 2n_{p_1} n_{r_1} n_{r_2} S_{p_1 r_1} v^{p_1 r_1}_{r_2 r_2}$
(9)	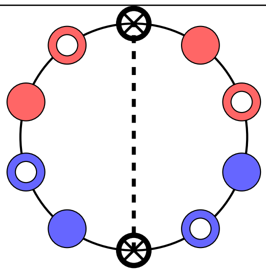	$\sum_{\substack{I_{P_1}\neq I_{P_2}\in G(A)\\ I_{R_1}\neq I_{R_2}\in G(B)}} \sum_{\substack{p_1\in I_{P_1} p_2\in I_{P_2}\\ r_1\in I_{R_1} r_2\in I_{R_2}}} -2n_{p_1} n_{r_1} n_{r_2} n_{p_2} S_{p_1 r_1} S_{r_2 p_2} v^{p_1 r_1}_{p_2 r_2}$
(10)	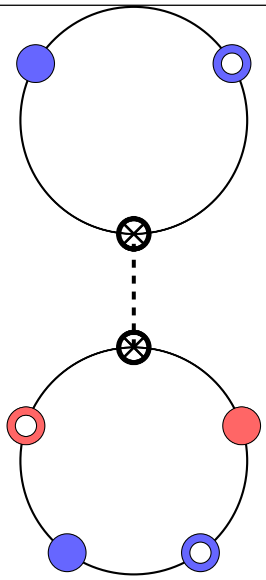	$\sum_{\substack{I_{P_1}\neq I_{P_2}\in G(A)\\ I_{R_1}\in G(B)}} \sum_{\substack{p_1\in I_{P_1} p_2\in I_{P_2}\\ r_1 s_1\in I_{R_1}}} -4n_{p_1} c_{r_1} n_{p_2} c_{s_1} S_{r_1 p_2} S_{p_2 s_1} v^{p_1 r_1}_{p_1 s_1}$

(11)	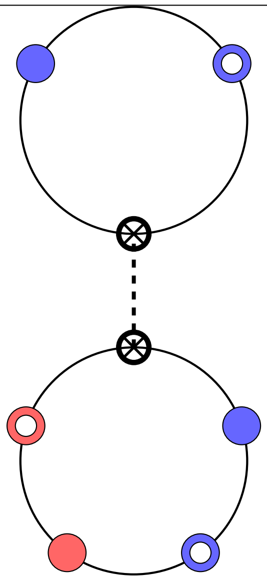	$\sum_{\substack{I_{P_1} \neq I_{P_2} \in G(A) \\ I_{R_1} \in G(B)}} \sum_{\substack{p_1 \in I_{P_1}\, p_2 \in I_{P_2} \\ r_1 \in I_{R_1}}} -4 n_{p_1} n_{r_1} n_{p_2} S_{r_1 p_2} v_{p_1 p_2}^{p_1 r_1}$
(12)	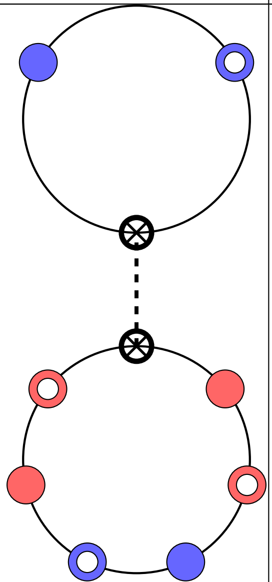	$\sum_{\substack{I_{P_1} \neq I_{P_2} \in G(A) \\ I_{R_1} \neq I_{R_2} \in G(B)}} \sum_{\substack{p_1 \in I_{P_1}\, p_2 \in I_{P_2} \\ r_1 \in I_{R_1}\, r_2 \in I_{R_2}}} 4 n_{p_1} n_{r_1} n_{p_2} n_{r_2} S_{r_1 p_2} S_{p_2 r_2} v_{p_1 r_2}^{p_1 r_1}$
(13)	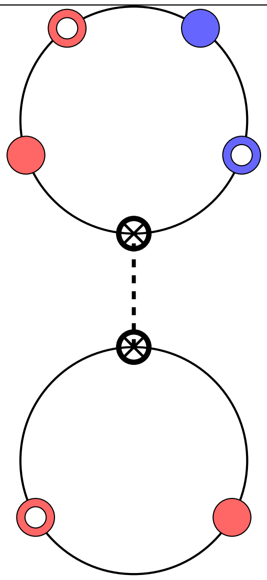	$\sum_{\substack{I_{P_1} \in G(A) \\ I_{R_1} \neq I_{R_2} \in G(B)}} \sum_{\substack{p_1 \in I_{P_1} \\ r_1 \in I_{R_1}\, r_2 \in I_{R_2}}} -4 n_{p_1} n_{r_1} n_{r_2} S_{p_1 r_1} v_{r_1 r_2}^{p_1 r_2}$

(14)	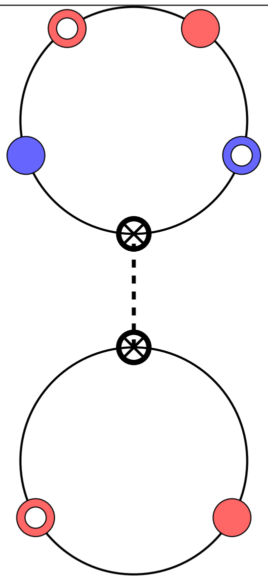	$\sum_{\substack{I_{P_1}\in G(A)\\ I_{R_1}\neq I_{R_2}\in G(B)}} \sum_{\substack{p_1 q_1\in I_{P_1}\\ r_1\in I_{R_1} r_2\in I_{R_2}}} -4c_{p_1} n_{r_1} c_{q_1} n_{r_2} S_{p_1 r_1} S_{r_1 q_1} v^{p_1 r_2}_{q_1 r_2}$
(15)	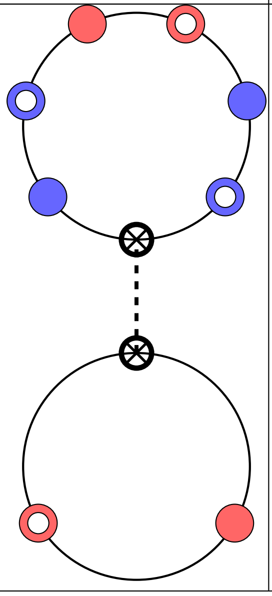	$\sum_{\substack{I_{P_1}\neq I_{P_2}\in G(A)\\ I_{R_1}\neq I_{R_2}\in G(B)}} \sum_{\substack{p_1\in I_{P_1} p_2\in I_{P_2}\\ r_1\in I_{R_1} r_2\in I_{R_2}}} 4n_{p_1} n_{r_1} n_{p_2} n_{r_2} S_{p_1 r_1} S_{r_1 p_2} v^{p_1 r_2}_{p_2 r_2}$

(16)	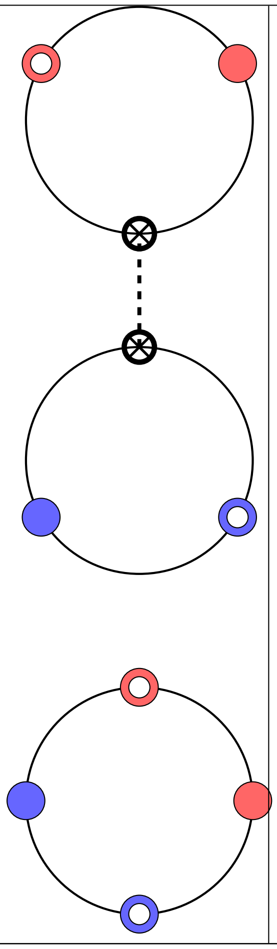	$\sum_{\substack{I_{P_1}\neq I_{P_2}\in G(A)\\ I_{R_1}\neq I_{R_2}\in G(B)}}\ \sum_{\substack{p_1\in I_{P_1}\, p_2\in I_{P_2}\\ r_1\in I_{R_1}\, r_2\in I_{R_2}}} -8 n_{r_1} n_{p_1} S_{p_1 r_1} S_{p_1 r_1} n_{p_2} n_{r_2} v^{p_2 r_2}_{p_2 r_2}$

$\langle V_A \mathcal{P}_2 \rangle$

(17)	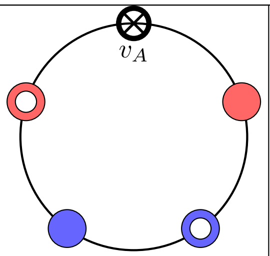	$\sum_{\substack{I_{P_1}\in G(A)\\ I_{R_1}\in G(B)}} \sum_{\substack{p_1\in I_{P_1}\\ r_1 s_1\in I_{R_1}}} -2c_{r_1} n_{p_1} c_{s_1} S_{r_1p_1} S_{p_1s_1} (v_A)_{r_1s_1}$
(18)	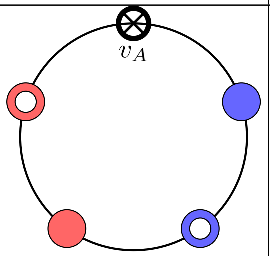	$\sum_{\substack{I_{P_1}\in G(A)\\ I_{R_1}\in G(B)}} \sum_{\substack{p_1\in I_{P_1}\\ r_1\in I_{R_1}}} -2n_{r_1} n_{p_1} S_{r_1p_1} (v_A)_{r_1p_1}$
(19)	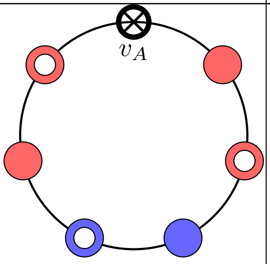	$\sum_{\substack{I_{P_1}\in G(A)\\ I_{R_1}\neq I_{R_2}\in G(B)}} \sum_{\substack{p_1\in I_{P_1}\\ r_1\in I_{R_1} r_2\in I_{R_2}}} 2n_{r_1} n_{p_1} n_{r_2} S_{r_1p_1} S_{p_1r_2} (v_A)_{r_1r_2}$
(20)	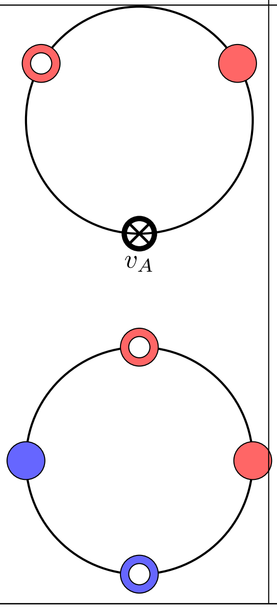	$\sum_{\substack{I_{P_1}\in G(A)\\ I_{R_1}\neq I_{R_2}\in G(B)}} \sum_{\substack{p_1\in I_{P_1}\\ r_1\in I_{R_1} r_2\in I_{R_2}}} -4n_{r_1} n_{p_1} S_{p_1r_1} S_{p_1r_1} n_{r_2} (v_A)_{r_2r_2}$

$\langle V_B \mathcal{P}_2 \rangle$

(21)	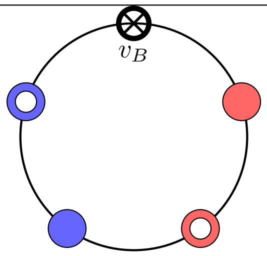	$$\sum_{\substack{I_{P_1}\in G(A)\\ I_{R_1}\in G(B)}} \sum_{\substack{p_1\in I_{P_1}\\ r_1\in I_{R_1}}} -2n_{p_1}n_{r_1}S_{p_1r_1}(v_B)_{p_1r_1}$$
(22)	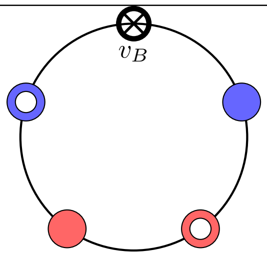	$$\sum_{\substack{I_{P_1}\in G(A)\\ I_{R_1}\in G(B)}} \sum_{\substack{p_1 q_1\in I_{P_1}\\ r_1\in I_{R_1}}} -2c_{p_1}n_{r_1}c_{q_1}S_{p_1r_1}S_{r_1q_1}(v_B)_{p_1q_1}$$
(23)	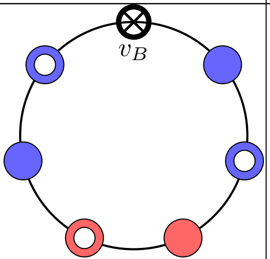	$$\sum_{\substack{I_{P_1}\neq I_{P_2}\in G(A)\\ I_{R_1}\in G(B)}} \sum_{\substack{p_1\in I_{P_1} p_2\in I_{P_2}\\ r_1\in I_{R_1}}} 2n_{p_1}n_{r_1}n_{p_2}S_{p_1r_1}S_{r_1p_2}(v_B)_{p_1p_2}$$
(24)	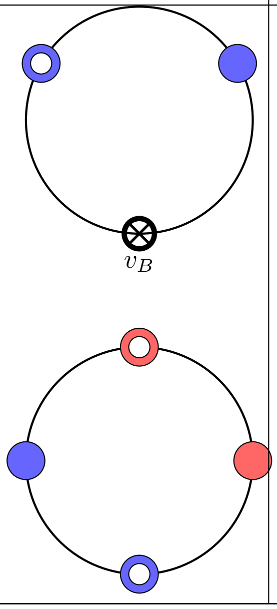	$$\sum_{\substack{I_{P_1}\neq I_{P_2}\in G(A)\\ I_{R_1}\in G(B)}} \sum_{\substack{p_1\in I_{P_1} p_2\in I_{P_2}\\ r_1\in I_{R_1}}} -4n_{r_1}n_{p_1}S_{p_1r_1}S_{p_1r_1}n_{p_2}(v_B)_{p_2p_2}$$

$\langle \mathcal{P}_2 \rangle$

(25)	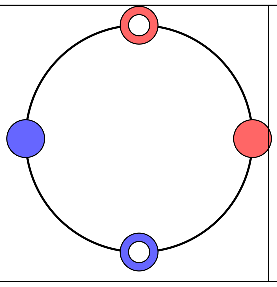	$$\sum_{\substack{I_{P_1}\in G(A)\\ I_{R_1}\in G(B)}} \sum_{p_1\in I_{P_1} r_1\in I_{R_1}} -2n_{r_1}n_{p_1}S_{p_1r_1}S_{p_1r_1}$$

$\langle V_{ee}\mathcal{P}_4\rangle$

(26)	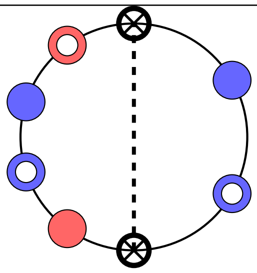	$\sum_{\substack{I_{P_1}\neq I_{P_2}\in G(A)\\ I_{R_1}\in G(B)}}\sum_{\substack{p_1\in I_{P_1}\,p_2\in I_{P_2}\\ r_1 s_1\in I_{R_1}}} 2n_{p_1}c_{r_1}n_{p_2}c_{s_1}S_{r_1p_2}S_{p_2s_1}v^{p_1p_1}_{s_1r_1}$
(27)	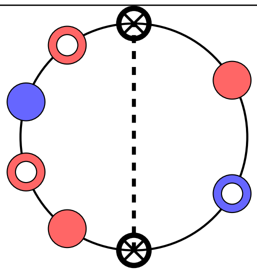	$\sum_{\substack{I_{P_1}\in G(A)\\ I_{R_1}\neq I_{R_2}\in G(B)}}\sum_{\substack{p_1q_1\in I_{P_1}\\ r_1\in I_{R_1}\,r_2s_2\in I_{R_2}}} 2c_{p_1}c_{r_2}c_{s_2}c_{q_1}n_{r_1}S_{p_1r_2}S_{s_2q_1}S_{q_1r_1}v^{p_1r_2}_{r_1s_2}$
(28)	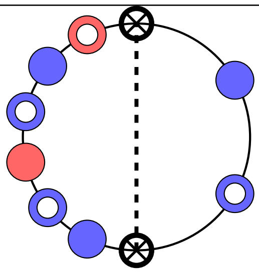	$\sum_{\substack{I_{P_1}\neq I_{P_2}\neq I_{P_3}\in G(A)\\ I_{R_1}\in G(B)}}\sum_{\substack{p_1\in I_{P_1}\,p_2\in I_{P_2}\,p_3\in I_{P_3}\\ r_1s_1\in I_{R_1}}} -2n_{p_1}c_{r_1}n_{p_2}c_{s_1}n_{p_3}S_{r_1p_2}S_{p_2s_1}S_{s_1p_3}v^{p_1p_1}_{p_3r_1}$
(29)	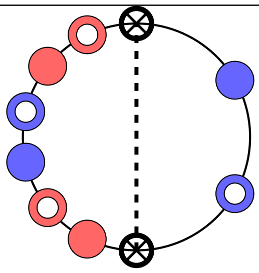	$\sum_{\substack{I_{P_1}\neq I_{P_2}\in G(A)\\ I_{R_1}\neq I_{R_2}\in G(B)}}\sum_{\substack{p_1\in I_{P_1}\,p_2\in I_{P_2}\\ r_1\in I_{R_1}\,r_2\in I_{R_2}}} -2n_{p_1}n_{r_1}n_{p_2}n_{r_2}S_{r_1p_2}S_{p_2r_2}v^{p_1p_1}_{r_2r_1}$
(30)	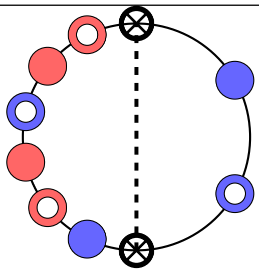	$\sum_{\substack{I_{P_1}\neq I_{P_2}\in G(A)\\ I_{R_1}\neq I_{R_2}\in G(B)}}\sum_{\substack{p_1\in I_{P_1}\,p_2q_2\in I_{P_2}\\ r_1\in I_{R_1}\,r_2\in I_{R_2}}} -2n_{p_1}n_{r_1}c_{p_2}n_{r_2}c_{q_2}S_{r_1p_2}S_{p_2r_2}S_{r_2q_2}v^{p_1p_1}_{q_2r_1}$
(31)	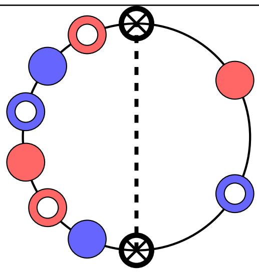	$\sum_{\substack{I_{P_1}\neq I_{P_2}\in G(A)\\ I_{R_1}\neq I_{R_2}\in G(B)}}\sum_{\substack{p_1\in I_{P_1}\,p_2q_2\in I_{P_2}\\ r_1\in I_{R_1}\,r_2s_2\in I_{R_2}}} -2c_{p_2}c_{r_2}c_{s_2}n_{p_1}n_{r_1}c_{q_2}S_{p_2r_2}S_{s_2p_1}S_{p_1r_1}S_{r_1q_2}v^{p_2r_2}_{q_2s_2}$

(32)	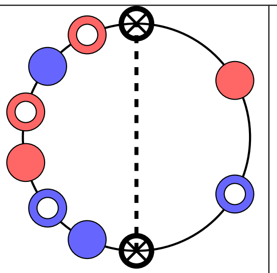	$\displaystyle\sum_{\substack{I_{P_1}\neq I_{P_2}\in G(A)\\ I_{R_1}\neq I_{R_2}\in G(B)}} \sum_{\substack{p_1\in I_{P_1}\, p_2q_2\in I_{P_2}\\ r_1\in I_{R_1}\, r_2s_2\in I_{R_2}}} -2c_{p_2}c_{r_2}c_{s_2}c_{q_2}n_{r_1}n_{p_1}S_{p_2r_2}S_{s_2q_2}S_{q_2r_1}S_{r_1p_1}v^{p_2r_2}_{p_1s_2}$
(33)	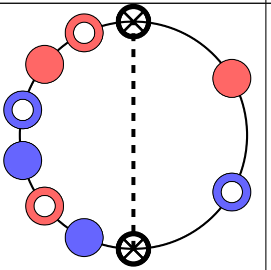	$\displaystyle\sum_{\substack{I_{P_1}\neq I_{P_2}\in G(A)\\ I_{R_1}\neq I_{R_2}\in G(B)}} \sum_{\substack{p_1\in I_{P_1}\, p_2q_2\in I_{P_2}\\ r_1\in I_{R_1}\, r_2s_2\in I_{R_2}}} -2c_{p_2}c_{r_2}n_{r_1}n_{p_1}c_{s_2}c_{q_2}S_{p_2r_2}S_{r_1p_1}S_{p_1s_2}S_{s_2q_2}v^{p_2r_2}_{q_2r_1}$
(34)	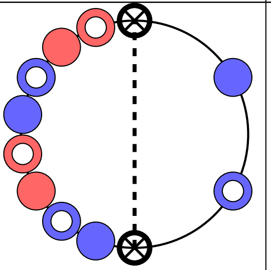	$\displaystyle\sum_{\substack{I_{P_1}\neq I_{P_2}\neq I_{P_3}\in G(A)\\ I_{R_1}\neq I_{R_2}\in G(B)}} \sum_{\substack{p_1\in I_{P_1}\, p_2\in I_{P_2}\, p_3\in I_{P_3}\\ r_1\in I_{R_1}\, r_2\in I_{R_2}}} 2n_{p_1}n_{r_1}n_{p_2}n_{r_2}n_{p_3}S_{r_1p_2}S_{p_2r_2}S_{r_2p_3}v^{p_1p_1}_{p_3r_1}$
(35)	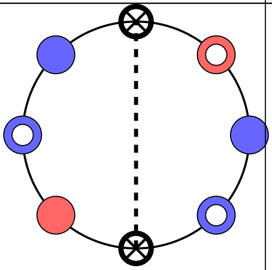	$\displaystyle\sum_{\substack{I_{P_1}\neq I_{P_2}\in G(A)\\ I_{R_1}\in G(B)}} \sum_{\substack{p_1\in I_{P_1}\, p_2\in I_{P_2}\\ r_1s_1\in I_{R_1}}} 2n_{p_1}c_{r_1}n_{p_2}c_{s_1}S_{p_1r_1}S_{p_2s_1}v^{p_1r_1}_{s_1p_2}$
(36)	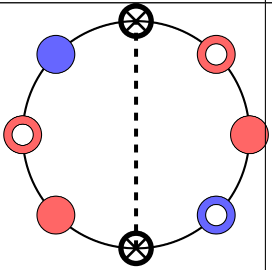	$\displaystyle\sum_{\substack{I_{P_1}\in G(A)\\ I_{R_1}\neq I_{R_2}\in G(B)}} \sum_{\substack{p_1q_1\in I_{P_1}\\ r_1\in I_{R_1}\, r_2\in I_{R_2}}} 2c_{p_1}n_{r_1}c_{q_1}n_{r_2}S_{p_1r_1}S_{q_1r_2}v^{p_1r_1}_{r_2q_1}$
(37)	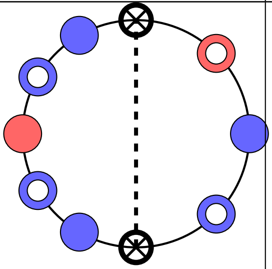	$\displaystyle\sum_{\substack{I_{P_1}\neq I_{P_2}\neq I_{P_3}\in G(A)\\ I_{R_1}\in G(B)}} \sum_{\substack{p_1\in I_{P_1}\, p_2\in I_{P_2}\, p_3\in I_{P_3}\\ r_1s_1\in I_{R_1}}} -2n_{p_1}c_{r_1}n_{p_2}c_{s_1}n_{p_3}S_{p_1r_1}S_{p_2s_1}S_{s_1p_3}v^{p_1r_1}_{p_3p_2}$
(38)	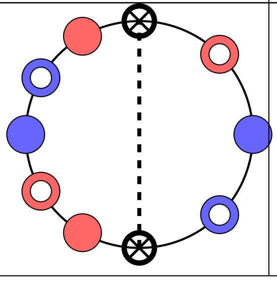	$\displaystyle\sum_{\substack{I_{P_1}\neq I_{P_2}\in G(A)\\ I_{R_1}\neq I_{R_2}\in G(B)}} \sum_{\substack{p_1\in I_{P_1}\, p_2\in I_{P_2}\\ r_1\in I_{R_1}\, r_2s_2\in I_{R_2}}} -2n_{p_1}c_{r_2}c_{s_2}n_{p_2}n_{r_1}S_{p_1r_2}S_{s_2p_2}S_{p_2r_1}v^{p_1r_2}_{r_1s_2}$

(39)	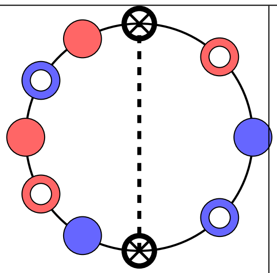	$\sum_{\substack{I_{P_1}\neq I_{P_2}\in G(A)\\ I_{R_1}\neq I_{R_2}\in G(B)}} \sum_{\substack{p_1\in I_{P_1}\, p_2q_2\in I_{P_2}\\ r_1\in I_{R_1}\, r_2s_2\in I_{R_2}}} -2n_{p_1}c_{r_2}c_{s_2}c_{p_2}n_{r_1}c_{q_2}S_{p_1r_2}S_{s_2p_2}S_{p_2r_1}S_{r_1q_2}v^{p_1r_2}_{q_2s_2}$
(40)	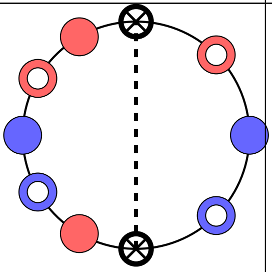	$\sum_{\substack{I_{P_1}\neq I_{P_2}\in G(A)\\ I_{R_1}\neq I_{R_2}\in G(B)}} \sum_{\substack{p_1\in I_{P_1}\, p_2\in I_{P_2}\\ r_1\in I_{R_1}\, r_2s_2\in I_{R_2}}} -2n_{p_1}c_{r_2}n_{r_1}n_{p_2}c_{s_2}S_{p_1r_2}S_{r_1p_2}S_{p_2s_2}v^{p_1r_2}_{s_2r_1}$
(41)	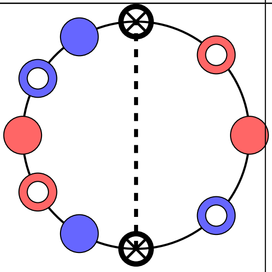	$\sum_{\substack{I_{P_1}\neq I_{P_2}\in G(A)\\ I_{R_1}\neq I_{R_2}\in G(B)}} \sum_{\substack{p_1\in I_{P_1}\, p_2q_2\in I_{P_2}\\ r_1\in I_{R_1}\, r_2\in I_{R_2}}} -2c_{p_2}n_{r_1}n_{p_1}n_{r_2}c_{q_2}S_{p_2r_1}S_{p_1r_2}S_{r_2q_2}v^{p_2r_1}_{q_2p_1}$
(42)	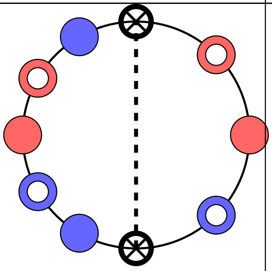	$\sum_{\substack{I_{P_1}\neq I_{P_2}\in G(A)\\ I_{R_1}\neq I_{R_2}\in G(B)}} \sum_{\substack{p_1\in I_{P_1}\, p_2q_2\in I_{P_2}\\ r_1\in I_{R_1}\, r_2\in I_{R_2}}} -2c_{p_2}n_{r_1}c_{q_2}n_{r_2}n_{p_1}S_{p_2r_1}S_{q_2r_2}S_{r_2p_1}v^{p_2r_1}_{p_1q_2}$
(43)	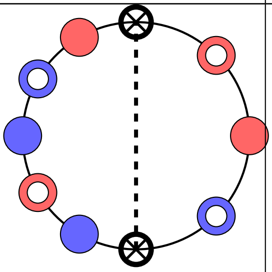	$\sum_{\substack{I_{P_1}\neq I_{P_2}\in G(A)\\ I_{R_1}\neq I_{R_2}\in G(B)}} \sum_{\substack{p_1\in I_{P_1}\, p_2q_2\in I_{P_2}\\ r_1\in I_{R_1}\, r_2s_2\in I_{R_2}}} -2c_{p_2}n_{r_1}c_{r_2}n_{p_1}c_{s_2}c_{q_2}S_{p_2r_1}S_{r_2p_1}S_{p_1s_2}S_{s_2q_2}v^{p_2r_1}_{q_2r_2}$
(44)	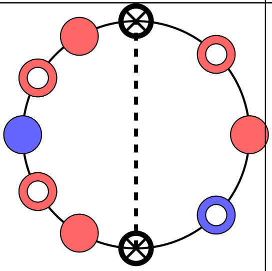	$\sum_{\substack{I_{P_1}\in G(A)\\ I_{R_1}\neq I_{R_2}\neq I_{R_3}\in G(B)}} \sum_{\substack{p_1q_1\in I_{P_1}\\ r_1\in I_{R_1}\, r_2\in I_{R_2}\, r_3\in I_{R_3}}} -2c_{p_1}n_{r_1}n_{r_2}c_{q_1}n_{r_3}S_{p_1r_1}S_{r_2q_1}S_{q_1r_3}v^{p_1r_1}_{r_3r_2}$
(45)	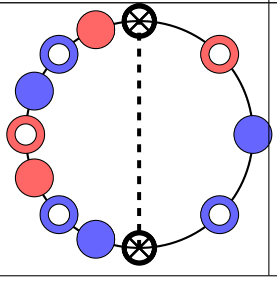	$\sum_{\substack{I_{P_1}\neq I_{P_2}\neq I_{P_3}\in G(A)\\ I_{R_1}\neq I_{R_2}\in G(B)}} \sum_{\substack{p_1\in I_{P_1}\, p_2\in I_{P_2}\, p_3\in I_{P_3}\\ r_1\in I_{R_1}\, r_2s_2\in I_{R_2}}} 2n_{p_1}c_{r_2}c_{s_2}n_{p_2}n_{r_1}n_{p_3}S_{p_1r_2}S_{s_2p_2}S_{p_2r_1}S_{r_1p_3}v^{p_1r_2}_{p_3s_2}$

(46)	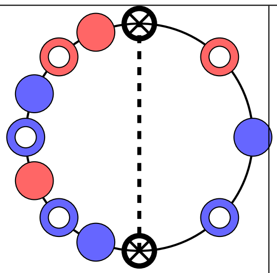	$\sum_{\substack{I_{P_1} \neq I_{P_2} \neq I_{P_3} \in G(A) \\ I_{R_1} \neq I_{R_2} \in G(B)}} \sum_{\substack{p_1 \in I_{P_1} p_2 \in I_{P_2} p_3 \in I_{P_3} \\ r_1 \in I_{R_1} r_2 s_2 \in I_{R_2}}} 2 n_{p_1} c_{r_2} n_{r_1} n_{p_2} c_{s_2} n_{p_3} S_{p_1 r_2} S_{r_1 p_2} S_{p_2 s_2} S_{s_2 p_3} v^{p_1 r_2}_{p_3 r_1}$
(47)	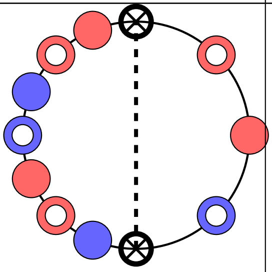	$\sum_{\substack{I_{P_1} \neq I_{P_2} \in G(A) \\ I_{R_1} \neq I_{R_2} \neq I_{R_3} \in G(B)}} \sum_{\substack{p_1 \in I_{P_1} p_2 q_2 \in I_{P_2} \\ r_1 \in I_{R_1} r_2 \in I_{R_2} r_3 \in I_{R_3}}} 2 c_{p_2} n_{r_1} n_{r_2} n_{p_1} n_{r_3} c_{q_2} S_{p_2 r_1} S_{r_2 p_1} S_{p_1 r_3} S_{r_3 q_2} v^{p_2 r_1}_{q_2 r_2}$
(48)	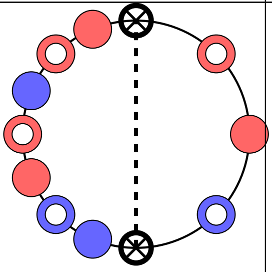	$\sum_{\substack{I_{P_1} \neq I_{P_2} \in G(A) \\ I_{R_1} \neq I_{R_2} \neq I_{R_3} \in G(B)}} \sum_{\substack{p_1 \in I_{P_1} p_2 q_2 \in I_{P_2} \\ r_1 \in I_{R_1} r_2 \in I_{R_2} r_3 \in I_{R_3}}} 2 c_{p_2} n_{r_1} n_{r_2} c_{q_2} n_{r_3} n_{p_1} S_{p_2 r_1} S_{r_2 q_2} S_{q_2 r_3} S_{r_3 p_1} v^{p_2 r_1}_{p_1 r_2}$
(49)	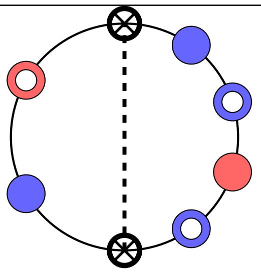	$\sum_{\substack{I_{P_1} \neq I_{P_2} \in G(A) \\ I_{R_1} \in G(B)}} \sum_{\substack{p_1 \in I_{P_1} p_2 q_2 \in I_{P_2} \\ r_1 s_1 \in I_{R_1}}} 2 c_{p_2} c_{r_1} n_{p_1} c_{s_1} c_{q_2} S_{p_2 r_1} S_{r_1 p_1} S_{s_1 q_2} v^{p_2 p_1}_{q_2 s_1}$
(50)	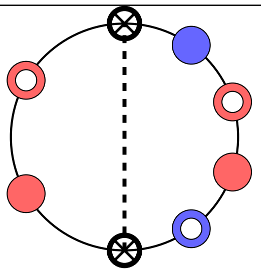	$\sum_{\substack{I_{P_1} \in G(A) \\ I_{R_1} \neq I_{R_2} \in G(B)}} \sum_{\substack{p_1 q_1 \in I_{P_1} \\ r_1 \in I_{R_1} r_2 \in I_{R_2}}} 2 c_{p_1} n_{r_1} c_{q_1} n_{r_2} S_{p_1 r_1} S_{r_1 q_1} v^{p_1 q_1}_{r_2 r_2}$
(51)	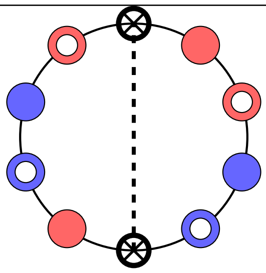	$\sum_{\substack{I_{P_1} \neq I_{P_2} \in G(A) \\ I_{R_1} \neq I_{R_2} \in G(B)}} \sum_{\substack{p_1 \in I_{P_1} p_2 \in I_{P_2} \\ r_1 \in I_{R_1} r_2 s_2 \in I_{R_2}}} -2 n_{p_1} n_{r_1} c_{r_2} n_{p_2} c_{s_2} S_{p_1 r_1} S_{r_2 p_2} S_{p_2 s_2} v^{p_1 r_1}_{s_2 r_2}$
(52)	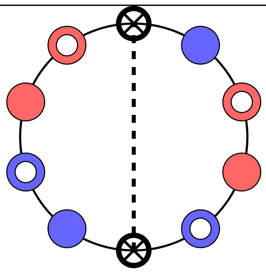	$\sum_{\substack{I_{P_1} \neq I_{P_2} \in G(A) \\ I_{R_1} \neq I_{R_2} \in G(B)}} \sum_{\substack{p_1 \in I_{P_1} p_2 q_2 \in I_{P_2} \\ r_1 \in I_{R_1} r_2 \in I_{R_2}}} -2 c_{p_2} n_{r_1} c_{q_2} n_{r_2} n_{p_1} S_{p_2 r_1} S_{r_1 q_2} S_{r_2 p_1} v^{p_2 q_2}_{p_1 r_2}$

(53)	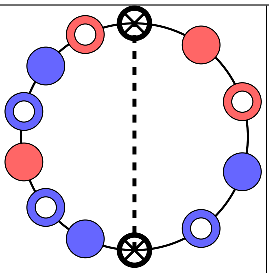	$\sum\limits_{\substack{I_{P_1}\neq I_{P_2}\neq I_{P_3}\in G(A)\\ I_{R_1}\neq I_{R_2}\in G(B)}} \sum\limits_{\substack{p_1\in I_{P_1}\, p_2\in I_{P_2}\, p_3\in I_{P_3}\\ r_1\in I_{R_1}\, r_2 s_2\in I_{R_2}}} 2n_{p_1}n_{r_1}c_{r_2}n_{p_2}c_{s_2}n_{p_3}S_{p_1r_1}S_{r_2p_2}S_{p_2s_2}S_{s_2p_3}v^{p_1r_1}_{p_3r_2}$
(54)	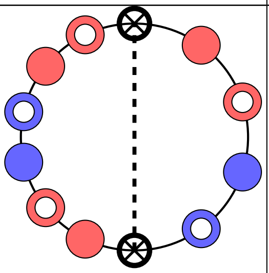	$\sum\limits_{\substack{I_{P_1}\neq I_{P_2}\in G(A)\\ I_{R_1}\neq I_{R_2}\neq I_{R_3}\in G(B)}} \sum\limits_{\substack{p_1\in I_{P_1}\, p_2\in I_{P_2}\\ r_1\in I_{R_1}\, r_2\in I_{R_2}\, r_3\in I_{R_3}}} 2n_{p_1}n_{r_1}n_{r_2}n_{p_2}n_{r_3}S_{p_1r_1}S_{r_2p_2}S_{p_2r_3}v^{p_1r_1}_{r_3r_2}$
(55)	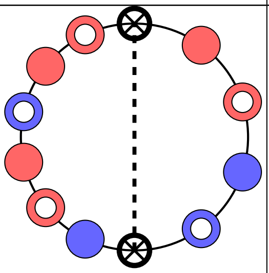	$\sum\limits_{\substack{I_{P_1}\neq I_{P_2}\in G(A)\\ I_{R_1}\neq I_{R_2}\neq I_{R_3}\in G(B)}} \sum\limits_{\substack{p_1\in I_{P_1}\, p_2 q_2\in I_{P_2}\\ r_1\in I_{R_1}\, r_2\in I_{R_2}\, r_3\in I_{R_3}}} 2n_{p_1}n_{r_1}n_{r_2}c_{p_2}n_{r_3}c_{q_2}S_{p_1r_1}S_{r_2p_2}S_{p_2r_3}S_{r_3q_2}v^{p_1r_1}_{q_2r_2}$
(56)	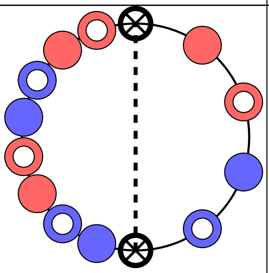	$\sum\limits_{\substack{I_{P_1}\neq I_{P_2}\neq I_{P_3}\in G(A)\\ I_{R_1}\neq I_{R_2}\neq I_{R_3}\in G(B)}} \sum\limits_{\substack{p_1\in I_{P_1}\, p_2\in I_{P_2}\, p_3\in I_{P_3}\\ r_1\in I_{R_1}\, r_2\in I_{R_2}\, r_3\in I_{R_3}}} -2n_{p_1}n_{r_1}n_{r_2}n_{p_2}n_{r_3}n_{p_3}S_{p_1r_1}S_{r_2p_2}S_{p_2r_3}S_{r_3p_3}v^{p_1r_1}_{p_3r_2}$
(57)	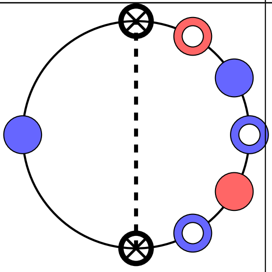	$\sum\limits_{\substack{I_{P_1}\neq I_{P_2}\in G(A)\\ I_{R_1}\in G(B)}} \sum\limits_{\substack{p_1\in I_{P_1}\, p_2 q_2\in I_{P_2}\\ r_1 s_1\in I_{R_1}}} 2c_{p_2}c_{r_1}n_{p_1}c_{s_1}c_{q_2}S_{p_2r_1}S_{r_1p_1}S_{p_1s_1}v^{p_2s_1}_{q_2q_2}$
(58)	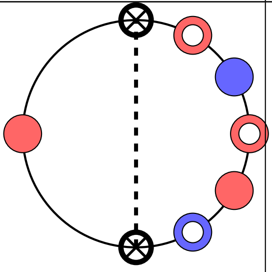	$\sum\limits_{\substack{I_{P_1}\in G(A)\\ I_{R_1}\neq I_{R_2}\in G(B)}} \sum\limits_{\substack{p_1 q_1\in I_{P_1}\\ r_1\in I_{R_1}\, r_2 s_2\in I_{R_2}}} 2c_{p_1}n_{r_1}c_{q_1}c_{r_2}c_{s_2}S_{p_1r_1}S_{r_1q_1}S_{q_1r_2}v^{p_1r_2}_{s_2s_2}$
(59)	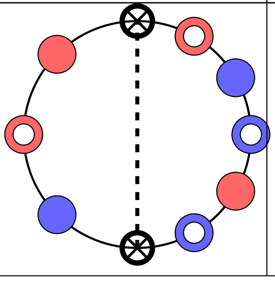	$\sum\limits_{\substack{I_{P_1}\neq I_{P_2}\in G(A)\\ I_{R_1}\neq I_{R_2}\in G(B)}} \sum\limits_{\substack{p_1\in I_{P_1}\, p_2 q_2\in I_{P_2}\\ r_1\in I_{R_1}\, r_2 s_2\in I_{R_2}}} -2c_{p_2}c_{r_2}n_{p_1}c_{s_2}n_{r_1}c_{q_2}S_{p_2r_2}S_{r_2p_1}S_{p_1s_2}S_{r_1q_2}v^{p_2s_2}_{q_2r_1}$

(60)	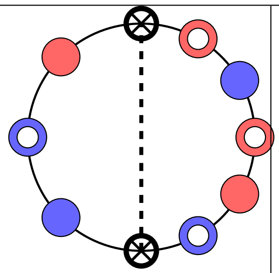	$\sum_{\substack{I_{P_1}\neq I_{P_2}\in G(A)\\ I_{R_1}\neq I_{R_2}\in G(B)}} \sum_{\substack{p_1\in I_{P_1}\, p_2q_2\in I_{P_2}\\ r_1\in I_{R_1}\, r_2s_2\in I_{R_2}}} -2c_{p_2}n_{r_1}c_{q_2}c_{r_2}c_{s_2}n_{p_1}S_{p_2r_1}S_{r_1q_2}S_{q_2r_2}S_{s_2p_1}v^{p_2r_2}_{p_1s_2}$
(61)	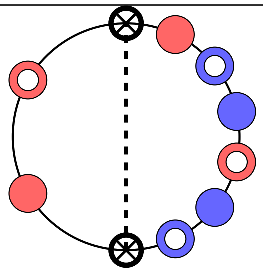	$\sum_{\substack{I_{P_1}\neq I_{P_2}\in G(A)\\ I_{R_1}\neq I_{R_2}\in G(B)}} \sum_{\substack{p_1\in I_{P_1}\, p_2\in I_{P_2}\\ r_1\in I_{R_1}\, r_2s_2\in I_{R_2}}} -2n_{p_1}c_{r_2}n_{p_2}c_{s_2}n_{r_1}S_{p_1r_2}S_{r_2p_2}S_{p_2s_2}v^{p_1s_2}_{r_1r_1}$
(62)	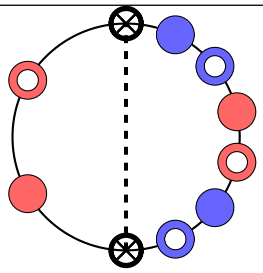	$\sum_{\substack{I_{P_1}\neq I_{P_2}\in G(A)\\ I_{R_1}\neq I_{R_2}\in G(B)}} \sum_{\substack{p_1\in I_{P_1}\, p_2\in I_{P_2}\\ r_1\in I_{R_1}\, r_2\in I_{R_2}}} -2n_{p_1}n_{r_1}n_{p_2}n_{r_2}S_{p_1r_1}S_{r_1p_2}v^{p_1p_2}_{r_2r_2}$
(63)	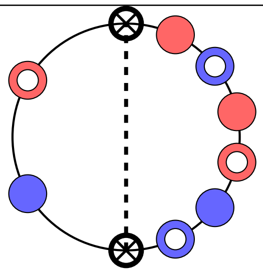	$\sum_{\substack{I_{P_1}\neq I_{P_2}\in G(A)\\ I_{R_1}\neq I_{R_2}\in G(B)}} \sum_{\substack{p_1\in I_{P_1}\, p_2q_2\in I_{P_2}\\ r_1\in I_{R_1}\, r_2s_2\in I_{R_2}}} -2n_{p_1}n_{r_1}c_{p_2}c_{r_2}c_{s_2}c_{q_2}S_{p_1r_1}S_{r_1p_2}S_{p_2r_2}S_{s_2q_2}v^{p_1r_2}_{q_2s_2}$
(64)	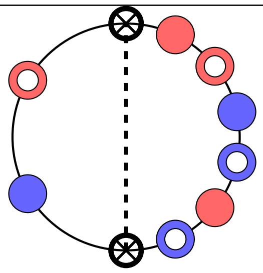	$\sum_{\substack{I_{P_1}\neq I_{P_2}\in G(A)\\ I_{R_1}\neq I_{R_2}\in G(B)}} \sum_{\substack{p_1\in I_{P_1}\, p_2q_2\in I_{P_2}\\ r_1\in I_{R_1}\, r_2s_2\in I_{R_2}}} -2c_{p_2}c_{r_2}n_{p_1}n_{r_1}c_{s_2}c_{q_2}S_{p_2r_2}S_{r_2p_1}S_{p_1r_1}S_{s_2q_2}v^{p_2r_1}_{q_2s_2}$
(65)	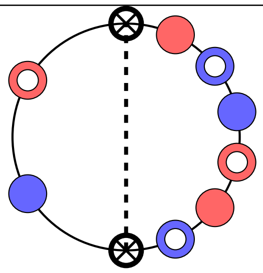	$\sum_{\substack{I_{P_1}\neq I_{P_2}\in G(A)\\ I_{R_1}\neq I_{R_2}\in G(B)}} \sum_{\substack{p_1\in I_{P_1}\, p_2q_2\in I_{P_2}\\ r_1\in I_{R_1}\, r_2s_2\in I_{R_2}}} -2c_{p_2}n_{r_1}n_{p_1}c_{r_2}c_{s_2}c_{q_2}S_{p_2r_1}S_{r_1p_1}S_{p_1r_2}S_{s_2q_2}v^{p_2r_2}_{q_2s_2}$
(66)	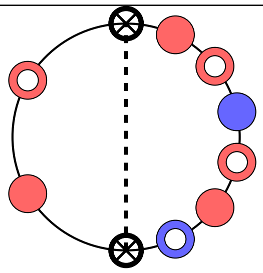	$\sum_{\substack{I_{P_1}\in G(A)\\ I_{R_1}\neq I_{R_2}\neq I_{R_3}\in G(B)}} \sum_{\substack{p_1q_1\in I_{P_1}\\ r_1\in I_{R_1}\, r_2\in I_{R_2}\, r_3\in I_{R_3}}} -2c_{p_1}n_{r_1}c_{q_1}n_{r_2}n_{r_3}S_{p_1r_1}S_{r_1q_1}S_{q_1r_2}v^{p_1r_2}_{r_3r_3}$

(67)	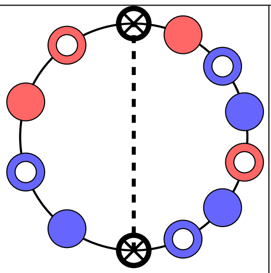	$\sum_{\substack{I_{P_1}\neq I_{P_2}\neq I_{P_3}\in G(A)\\ I_{R_1}\neq I_{R_2}\in G(B)}} \sum_{\substack{p_1\in I_{P_1}\, p_2\in I_{P_2}\, p_3\in I_{P_3}\\ r_1\in I_{R_1}\, r_2 s_2\in I_{R_2}}} 2n_{p_1}c_{r_2}n_{p_2}c_{s_2}n_{r_1}n_{p_3}S_{p_1r_2}S_{r_2p_2}S_{p_2s_2}S_{r_1p_3}v^{p_1s_2}_{p_3r_1}$
(68)	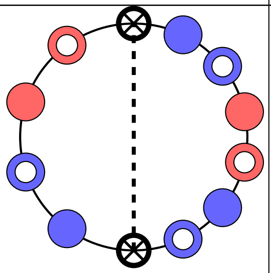	$\sum_{\substack{I_{P_1}\neq I_{P_2}\neq I_{P_3}\in G(A)\\ I_{R_1}\neq I_{R_2}\in G(B)}} \sum_{\substack{p_1\in I_{P_1}\, p_2\in I_{P_2}\, p_3\in I_{P_3}\\ r_1\in I_{R_1}\, r_2\in I_{R_2}}} 2n_{p_1}n_{r_1}n_{p_2}n_{r_2}n_{p_3}S_{p_1r_1}S_{r_1p_2}S_{r_2p_3}v^{p_1p_2}_{p_3r_2}$
(69)	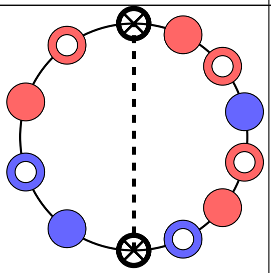	$\sum_{\substack{I_{P_1}\neq I_{P_2}\in G(A)\\ I_{R_1}\neq I_{R_2}\neq I_{R_3}\in G(B)}} \sum_{\substack{p_1\in I_{P_1}\, p_2 q_2\in I_{P_2}\\ r_1\in I_{R_1}\, r_2\in I_{R_2}\, r_3\in I_{R_3}}} 2c_{p_2}n_{r_1}c_{q_2}n_{r_2}n_{r_3}n_{p_1}S_{p_2r_1}S_{r_1q_2}S_{q_2r_2}S_{r_3p_1}v^{p_2r_2}_{p_1r_3}$
(70)	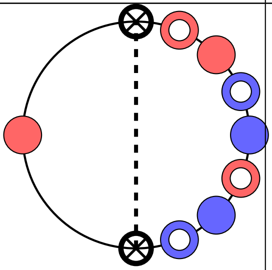	$\sum_{\substack{I_{P_1}\neq I_{P_2}\in G(A)\\ I_{R_1}\neq I_{R_2}\in G(B)}} \sum_{\substack{p_1\in I_{P_1}\, p_2\in I_{P_2}\\ r_1\in I_{R_1}\, r_2 s_2\in I_{R_2}}} -2n_{p_1}c_{r_2}n_{p_2}n_{r_1}c_{s_2}S_{p_1r_2}S_{r_2p_2}S_{p_2r_1}v^{p_1r_1}_{s_2s_2}$
(71)	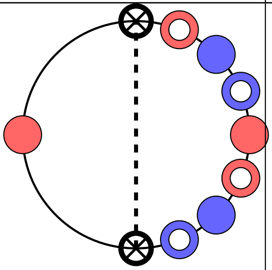	$\sum_{\substack{I_{P_1}\neq I_{P_2}\in G(A)\\ I_{R_1}\neq I_{R_2}\in G(B)}} \sum_{\substack{p_1\in I_{P_1}\, p_2\in I_{P_2}\\ r_1\in I_{R_1}\, r_2 s_2\in I_{R_2}}} -2n_{p_1}n_{r_1}n_{p_2}c_{r_2}c_{s_2}S_{p_1r_1}S_{r_1p_2}S_{p_2r_2}v^{p_1r_2}_{s_2s_2}$
(72)	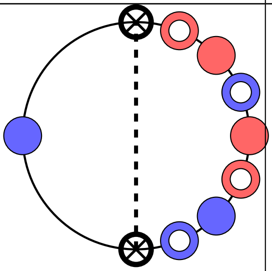	$\sum_{\substack{I_{P_1}\neq I_{P_2}\in G(A)\\ I_{R_1}\neq I_{R_2}\in G(B)}} \sum_{\substack{p_1\in I_{P_1}\, p_2 q_2\in I_{P_2}\\ r_1\in I_{R_1}\, r_2\in I_{R_2}}} -2n_{p_1}n_{r_1}c_{p_2}n_{r_2}c_{q_2}S_{p_1r_1}S_{r_1p_2}S_{p_2r_2}v^{p_1r_2}_{q_2q_2}$
(73)	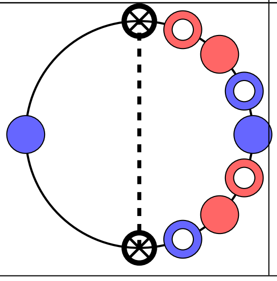	$\sum_{\substack{I_{P_1}\neq I_{P_2}\in G(A)\\ I_{R_1}\neq I_{R_2}\in G(B)}} \sum_{\substack{p_1\in I_{P_1}\, p_2 q_2\in I_{P_2}\\ r_1\in I_{R_1}\, r_2\in I_{R_2}}} -2c_{p_2}n_{r_1}n_{p_1}n_{r_2}c_{q_2}S_{p_2r_1}S_{r_1p_1}S_{p_1r_2}v^{p_2r_2}_{q_2q_2}$

(74)	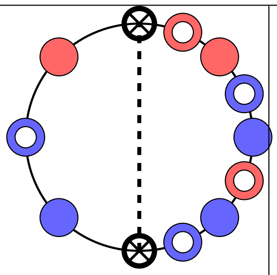	$\sum_{\substack{I_{P_1}\neq I_{P_2}\neq I_{P_3}\in G(A)\\ I_{R_1}\neq I_{R_2}\in G(B)}} \sum_{\substack{p_1\in I_{P_1} p_2\in I_{P_2} p_3\in I_{P_3}\\ r_1\in I_{R_1} r_2 s_2\in I_{R_2}}} 2n_{p_1}c_{r_2}n_{p_2}n_{r_1}c_{s_2}n_{p_3}S_{p_1r_2}S_{r_2p_2}S_{p_2r_1}S_{s_2p_3}v^{p_1r_1}_{p_3s_2}$
(75)	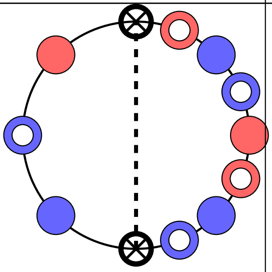	$\sum_{\substack{I_{P_1}\neq I_{P_2}\neq I_{P_3}\in G(A)\\ I_{R_1}\neq I_{R_2}\in G(B)}} \sum_{\substack{p_1\in I_{P_1} p_2\in I_{P_2} p_3\in I_{P_3}\\ r_1\in I_{R_1} r_2 s_2\in I_{R_2}}} 2n_{p_1}n_{r_1}n_{p_2}c_{r_2}c_{s_2}n_{p_3}S_{p_1r_1}S_{r_1p_2}S_{p_2r_2}S_{s_2p_3}v^{p_1r_2}_{p_3s_2}$
(76)	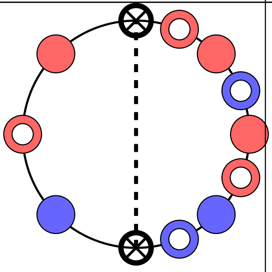	$\sum_{\substack{I_{P_1}\neq I_{P_2}\in G(A)\\ I_{R_1}\neq I_{R_2}\neq I_{R_3}\in G(B)}} \sum_{\substack{p_1\in I_{P_1} p_2 q_2\in I_{P_2}\\ r_1\in I_{R_1} r_2\in I_{R_2} r_3\in I_{R_3}}} 2n_{p_1}n_{r_1}c_{p_2}n_{r_2}n_{r_3}c_{q_2}S_{p_1r_1}S_{r_1p_2}S_{p_2r_2}S_{r_3q_2}v^{p_1r_2}_{q_2r_3}$
(77)	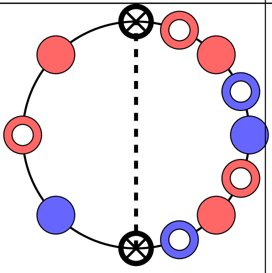	$\sum_{\substack{I_{P_1}\neq I_{P_2}\in G(A)\\ I_{R_1}\neq I_{R_2}\neq I_{R_3}\in G(B)}} \sum_{\substack{p_1\in I_{P_1} p_2 q_2\in I_{P_2}\\ r_1\in I_{R_1} r_2\in I_{R_2} r_3\in I_{R_3}}} 2c_{p_2}n_{r_1}n_{p_1}n_{r_2}n_{r_3}c_{q_2}S_{p_2r_1}S_{r_1p_1}S_{p_1r_2}S_{r_3q_2}v^{p_2r_2}_{q_2r_3}$
(78)	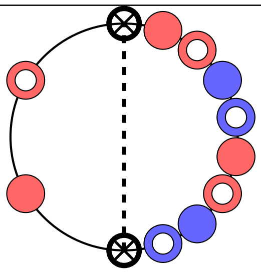	$\sum_{\substack{I_{P_1}\neq I_{P_2}\in G(A)\\ I_{R_1}\neq I_{R_2}\neq I_{R_3}\in G(B)}} \sum_{\substack{p_1\in I_{P_1} p_2\in I_{P_2}\\ r_1\in I_{R_1} r_2\in I_{R_2} r_3\in I_{R_3}}} 2n_{p_1}n_{r_1}n_{p_2}n_{r_2}n_{r_3}S_{p_1r_1}S_{r_1p_2}S_{p_2r_2}v^{p_1r_2}_{r_3r_3}$
(79)	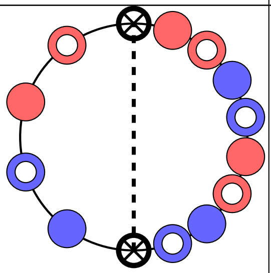	$\sum_{\substack{I_{P_1}\neq I_{P_2}\neq I_{P_3}\in G(A)\\ I_{R_1}\neq I_{R_2}\neq I_{R_3}\in G(B)}} \sum_{\substack{p_1\in I_{P_1} p_2\in I_{P_2} p_3\in I_{P_3}\\ r_1\in I_{R_1} r_2\in I_{R_2} r_3\in I_{R_3}}} -2n_{p_1}n_{r_1}n_{p_2}n_{r_2}n_{r_3}n_{p_3}S_{p_1r_1}S_{r_1p_2}S_{p_2r_2}S_{r_3p_3}v^{p_1r_2}_{p_3r_3}$

(80)	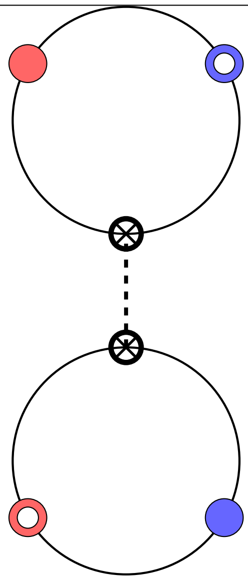	$\sum_{\substack{I_{P_1}\in G(A)\\ I_{R_1}\in G(B)}} \sum_{\substack{p_1q_1\in I_{P_1}\\ r_1s_1\in I_{R_1}}} 4c_{p_1}c_{r_1}c_{s_1}c_{q_1}S_{p_1r_1}S_{s_1q_1}v^{p_1s_1}_{r_1q_1}$
(81)	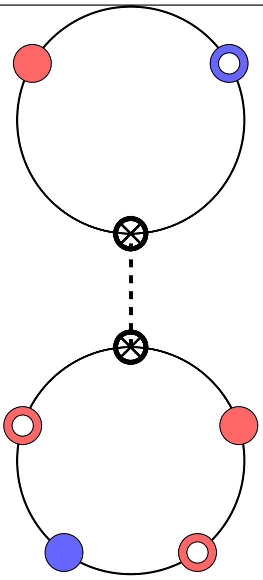	$\sum_{\substack{I_{P_1}\in G(A)\\ I_{R_1}\neq I_{R_2}\in G(B)}} \sum_{\substack{p_1q_1\in I_{P_1}\\ r_1\in I_{R_1}\, r_2s_2\in I_{R_2}}} -4c_{p_1}c_{r_2}c_{s_2}c_{q_1}n_{r_1}S_{p_1r_2}S_{s_2q_1}S_{q_1r_1}v^{p_1s_2}_{r_2r_1}$
(82)	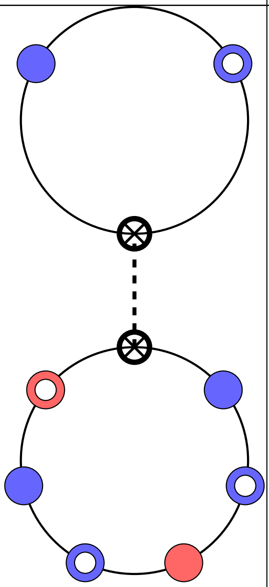	$\sum_{\substack{I_{P_1}\neq I_{P_2}\neq I_{P_3}\in G(A)\\ I_{R_1}\in G(B)}} \sum_{\substack{p_1\in I_{P_1}\, p_2\in I_{P_2}\, p_3\in I_{P_3}\\ r_1s_1\in I_{R_1}}} 4n_{p_1}c_{r_1}n_{p_2}c_{s_1}n_{p_3}S_{r_1p_2}S_{p_2s_1}S_{s_1p_3}v^{p_1r_1}_{p_1p_3}$

(83)	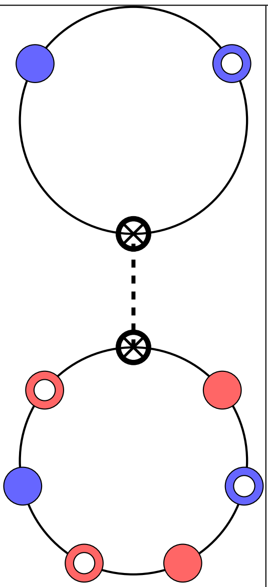	$\sum_{\substack{I_{P_1} \neq I_{P_2} \in G(A) \\ I_{R_1} \neq I_{R_2} \in G(B)}} \sum_{\substack{p_1 \in I_{P_1} p_2 q_2 \in I_{P_2} \\ r_1 \in I_{R_1} r_2 s_2 \in I_{R_2}}} 4 n_{p_1} c_{r_2} c_{p_2} n_{r_1} c_{q_2} c_{s_2} S_{r_2 p_2} S_{p_2 r_1} S_{r_1 q_2} S_{q_2 s_2} v_{p_1 s_2}^{p_1 r_2}$
(84)	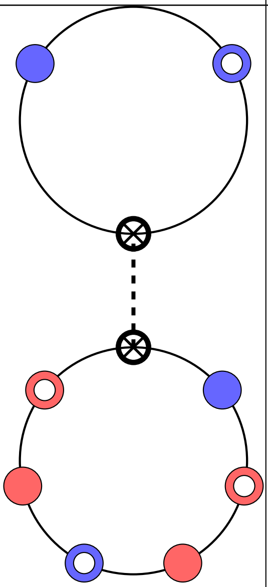	$\sum_{\substack{I_{P_1} \neq I_{P_2} \in G(A) \\ I_{R_1} \neq I_{R_2} \in G(B)}} \sum_{\substack{p_1 \in I_{P_1} p_2 q_2 \in I_{P_2} \\ r_1 \in I_{R_1} r_2 \in I_{R_2}}} 4 n_{p_1} n_{r_1} c_{p_2} n_{r_2} c_{q_2} S_{r_1 p_2} S_{p_2 r_2} S_{r_2 q_2} v_{p_1 q_2}^{p_1 r_1}$
(85)	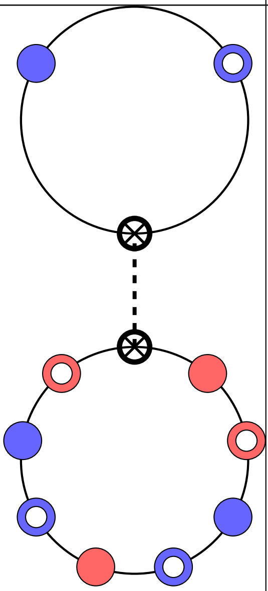	$\sum_{\substack{I_{P_1} \neq I_{P_2} \neq I_{P_3} \in G(A) \\ I_{R_1} \neq I_{R_2} \in G(B)}} \sum_{\substack{p_1 \in I_{P_1} p_2 \in I_{P_2} p_3 \in I_{P_3} \\ r_1 \in I_{R_1} r_2 s_2 \in I_{R_2}}} -4 n_{p_1} c_{r_2} n_{p_2} c_{s_2} n_{p_3} n_{r_1} S_{r_2 p_2} S_{p_2 s_2} S_{s_2 p_3} S_{p_3 r_1} v_{p_1 r_1}^{p_1 r_2}$

(86)	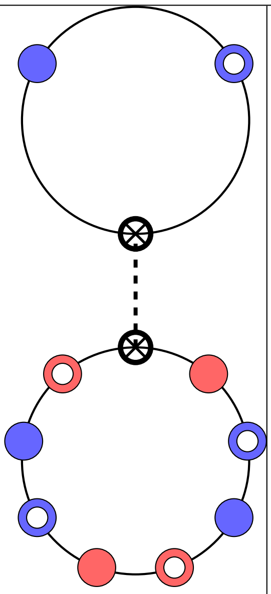	$\sum_{\substack{I_{P_1}\neq I_{P_2}\neq I_{P_3}\in G(A)\\ I_{R_1}\neq I_{R_2}\in G(B)}} \sum_{\substack{p_1\in I_{P_1} p_2\in I_{P_2} p_3\in I_{P_3}\\ r_1\in I_{R_1} r_2 s_2\in I_{R_2}}} -4n_{p_1}c_{r_2}n_{p_2}n_{r_1}n_{p_3}c_{s_2}S_{r_2p_2}S_{p_2r_1}S_{r_1p_3}S_{p_3s_2}v^{p_1r_2}_{p_1s_2}$
(87)	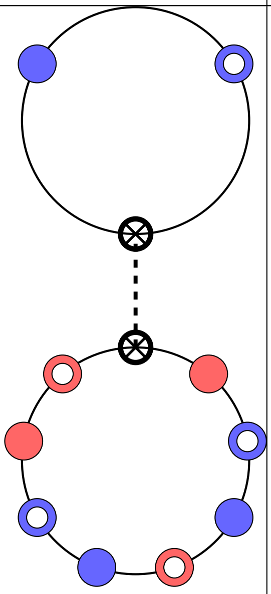	$\sum_{\substack{I_{P_1}\neq I_{P_2}\neq I_{P_3}\in G(A)\\ I_{R_1}\neq I_{R_2}\in G(B)}} \sum_{\substack{p_1\in I_{P_1} p_2\in I_{P_2} p_3\in I_{P_3}\\ r_1\in I_{R_1} r_2 s_2\in I_{R_2}}} -4n_{p_1}n_{r_1}n_{p_2}c_{r_2}n_{p_3}c_{s_2}S_{r_1p_2}S_{p_2r_2}S_{r_2p_3}S_{p_3s_2}v^{p_1r_1}_{p_1s_2}$
(88)	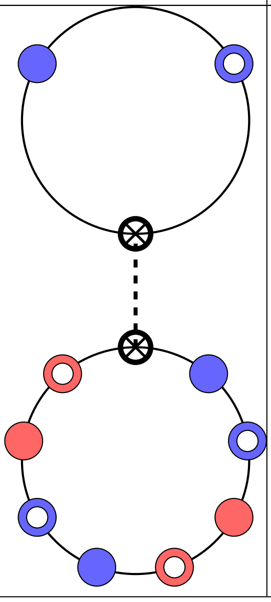	$\sum_{\substack{I_{P_1}\neq I_{P_2}\neq I_{P_3}\in G(A)\\ I_{R_1}\neq I_{R_2}\in G(B)}} \sum_{\substack{p_1\in I_{P_1} p_2\in I_{P_2} p_3\in I_{P_3}\\ r_1\in I_{R_1} r_2\in I_{R_2}}} -4n_{p_1}n_{r_1}n_{p_2}n_{r_2}n_{p_3}S_{r_1p_2}S_{p_2r_2}S_{r_2p_3}v^{p_1r_1}_{p_1p_3}$

(89)	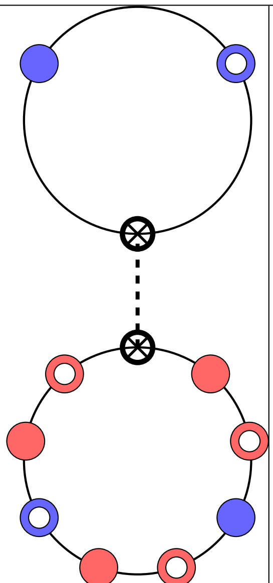	$\sum_{\substack{I_{P_1}\neq I_{P_2}\in G(A)\\ I_{R_1}\neq I_{R_2}\neq I_{R_3}\in G(B)}} \sum_{\substack{p_1\in I_{P_1}\, p_2q_2\in I_{P_2}\\ r_1\in I_{R_1}\, r_2\in I_{R_2}\, r_3\in I_{R_3}}} -4n_{p_1}n_{r_1}c_{p_2}n_{r_2}c_{q_2}n_{r_3}S_{r_1p_2}S_{p_2r_2}S_{r_2q_2}S_{q_2r_3}v^{p_1r_1}_{p_1r_3}$
(90)	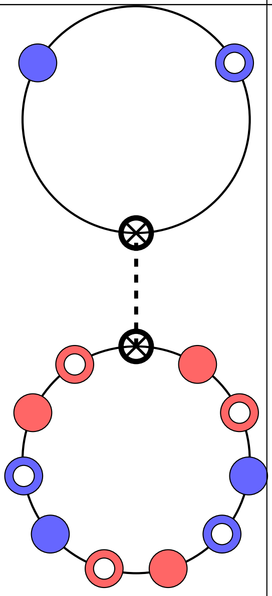	$\sum_{\substack{I_{P_1}\neq I_{P_2}\neq I_{P_3}\in G(A)\\ I_{R_1}\neq I_{R_2}\neq I_{R_3}\in G(B)}} \sum_{\substack{p_1\in I_{P_1}\, p_2\in I_{P_2}\, p_3\in I_{P_3}\\ r_1\in I_{R_1}\, r_2\in I_{R_2}\, r_3\in I_{R_3}}} 4n_{p_1}n_{r_1}n_{p_2}n_{r_2}n_{p_3}n_{r_3}S_{r_1p_2}S_{p_2r_2}S_{r_2p_3}S_{p_3r_3}v^{p_1r_1}_{p_1r_3}$
(91)	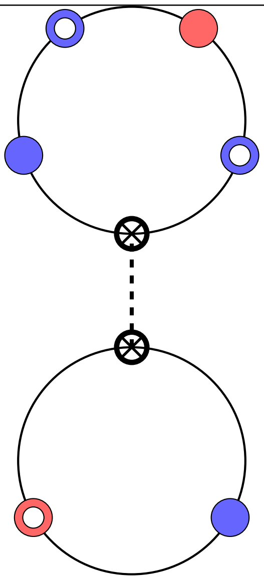	$\sum_{\substack{I_{P_1}\neq I_{P_2}\in G(A)\\ I_{R_1}\in G(B)}} \sum_{\substack{p_1\in I_{P_1}\, p_2q_2\in I_{P_2}\\ r_1s_1\in I_{R_1}}} -4c_{p_2}c_{r_1}n_{p_1}c_{s_1}c_{q_2}S_{p_2r_1}S_{r_1p_1}S_{s_1q_2}v^{p_2s_1}_{p_1q_2}$

(92)	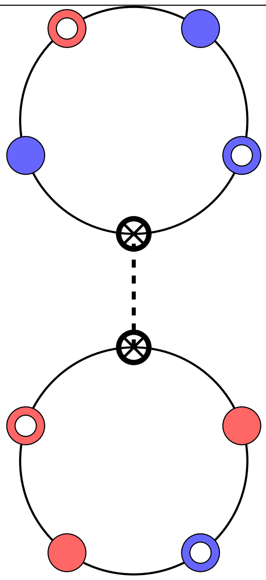	$$\sum_{\substack{I_{P_1}\neq I_{P_2}\in G(A)\\ I_{R_1}\neq I_{R_2}\in G(B)}} \sum_{\substack{p_1\in I_{P_1}\, p_2q_2\in I_{P_2}\\ r_1\in I_{R_1}\, r_2s_2\in I_{R_2}}} 4n_{p_1}c_{r_2}c_{p_2}n_{r_1}c_{q_2}c_{s_2}S_{p_1r_2}S_{r_2p_2}S_{r_1q_2}S_{q_2s_2}v^{p_1r_1}_{p_2s_2}$$
(93)	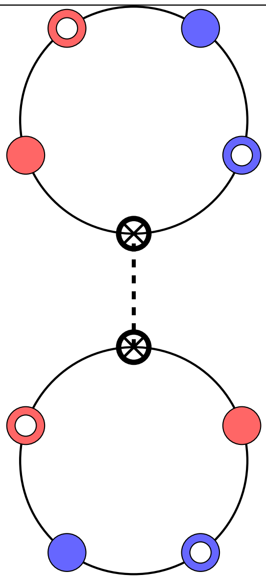	$$\sum_{\substack{I_{P_1}\neq I_{P_2}\in G(A)\\ I_{R_1}\neq I_{R_2}\in G(B)}} \sum_{\substack{p_1\in I_{P_1}\, p_2\in I_{P_2}\\ r_1\in I_{R_1}\, r_2s_2\in I_{R_2}}} 4n_{p_1}n_{r_1}c_{r_2}n_{p_2}c_{s_2}S_{p_1r_1}S_{r_2p_2}S_{p_2s_2}v^{p_1r_2}_{r_1s_2}$$
(94)	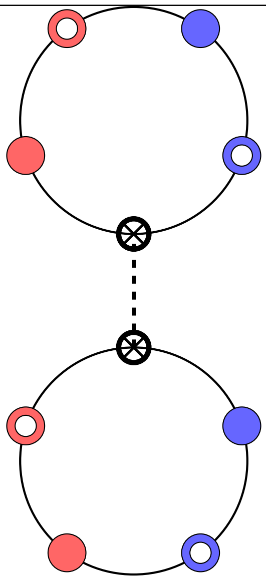	$$\sum_{\substack{I_{P_1}\neq I_{P_2}\in G(A)\\ I_{R_1}\neq I_{R_2}\in G(B)}} \sum_{\substack{p_1\in I_{P_1}\, p_2\in I_{P_2}\\ r_1\in I_{R_1}\, r_2\in I_{R_2}}} 4n_{p_1}n_{r_1}n_{r_2}n_{p_2}S_{p_1r_1}S_{r_2p_2}v^{p_1r_2}_{r_1p_2}$$

(95)	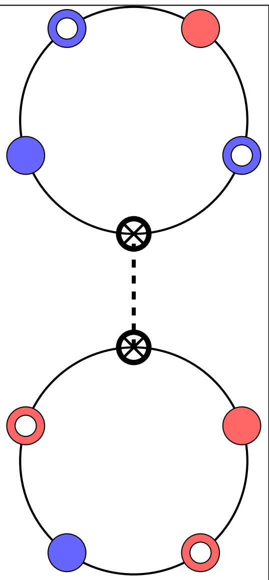	$\sum\limits_{\substack{I_{P_1}\neq I_{P_2}\in G(A)\\ I_{R_1}\neq I_{R_2}\in G(B)}} \sum\limits_{\substack{p_1\in I_{P_1}\, p_2q_2\in I_{P_2}\\ r_1\in I_{R_1}\, r_2s_2\in I_{R_2}}} 4c_{p_2}c_{r_2}n_{p_1}c_{s_2}c_{q_2}n_{r_1}S_{p_2r_2}S_{r_2p_1}S_{s_2q_2}S_{q_2r_1}v^{p_2s_2}_{p_1r_1}$
(96)	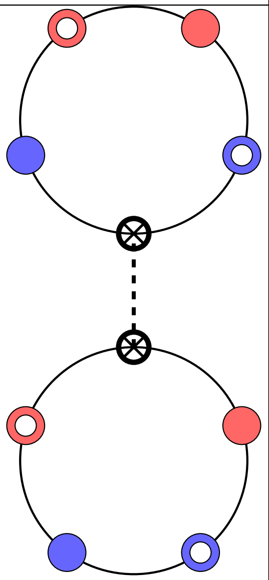	$\sum\limits_{\substack{I_{P_1}\neq I_{P_2}\in G(A)\\ I_{R_1}\neq I_{R_2}\in G(B)}} \sum\limits_{\substack{p_1\in I_{P_1}\, p_2q_2\in I_{P_2}\\ r_1\in I_{R_1}\, r_2s_2\in I_{R_2}}} 4c_{p_2}n_{r_1}c_{q_2}c_{r_2}n_{p_1}c_{s_2}S_{p_2r_1}S_{r_1q_2}S_{r_2p_1}S_{p_1s_2}v^{p_2r_2}_{q_2s_2}$
(97)	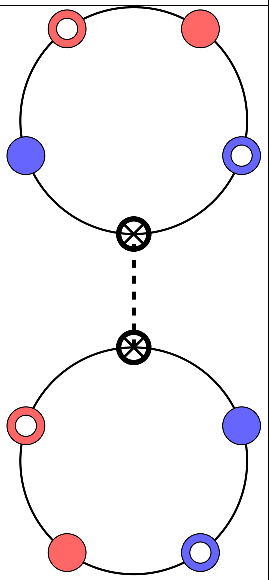	$\sum\limits_{\substack{I_{P_1}\neq I_{P_2}\in G(A)\\ I_{R_1}\neq I_{R_2}\in G(B)}} \sum\limits_{\substack{p_1\in I_{P_1}\, p_2q_2\in I_{P_2}\\ r_1\in I_{R_1}\, r_2\in I_{R_2}}} 4c_{p_2}n_{r_1}c_{q_2}n_{r_2}n_{p_1}S_{p_2r_1}S_{r_1q_2}S_{r_2p_1}v^{p_2r_2}_{q_2p_1}$

(98)	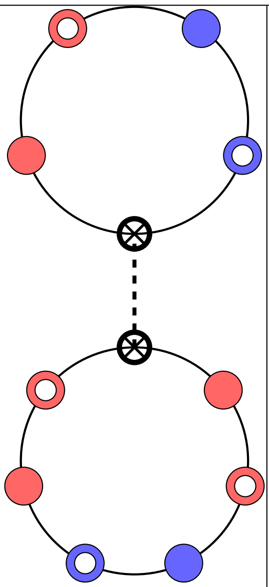	$\sum_{\substack{I_{P_1}\neq I_{P_2}\in G(A)\\ I_{R_1}\neq I_{R_2}\neq I_{R_3}\in G(B)}} \sum_{\substack{p_1\in I_{P_1} p_2\in I_{P_2}\\ r_1\in I_{R_1} r_2\in I_{R_2} r_3\in I_{R_3}}} -4 n_{p_1} n_{r_1} n_{r_2} n_{p_2} n_{r_3} S_{p_1 r_1} S_{r_2 p_2} S_{p_2 r_3} v^{p_1 r_2}_{r_1 r_3}$
(99)	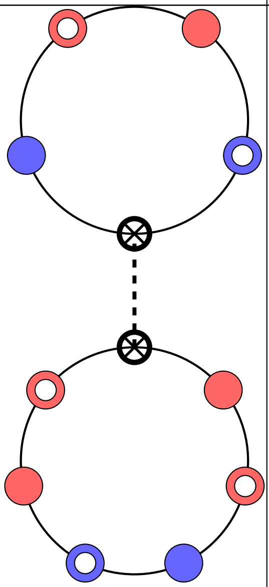	$\sum_{\substack{I_{P_1}\neq I_{P_2}\in G(A)\\ I_{R_1}\neq I_{R_2}\neq I_{R_3}\in G(B)}} \sum_{\substack{p_1\in I_{P_1} p_2 q_2\in I_{P_2}\\ r_1\in I_{R_1} r_2\in I_{R_2} r_3\in I_{R_3}}} -4 c_{p_2} n_{r_1} c_{q_2} n_{r_2} n_{p_1} n_{r_3} S_{p_2 r_1} S_{r_1 q_2} S_{r_2 p_1} S_{p_1 r_3} v^{p_2 r_2}_{q_2 r_3}$
(100)	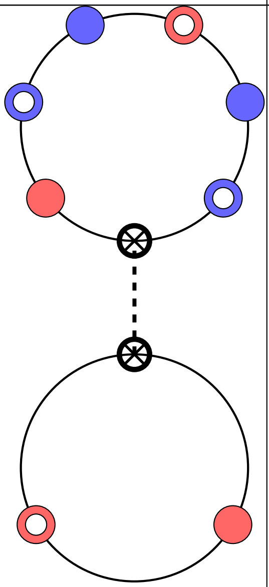	$\sum_{\substack{I_{P_1}\neq I_{P_2}\in G(A)\\ I_{R_1}\neq I_{R_2}\in G(B)}} \sum_{\substack{p_1\in I_{P_1} p_2\in I_{P_2}\\ r_1\in I_{R_1} r_2 s_2\in I_{R_2}}} 4 n_{p_1} c_{r_2} n_{p_2} c_{s_2} n_{r_1} S_{p_1 r_2} S_{r_2 p_2} S_{p_2 s_2} v^{p_1 r_1}_{s_2 r_1}$

(101)	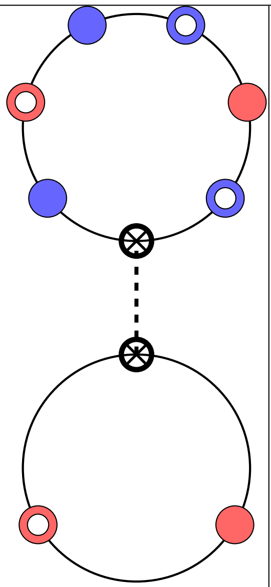	$\sum_{\substack{I_{P_1}\neq I_{P_2}\in G(A)\\ I_{R_1}\neq I_{R_2}\in G(B)}} \sum_{\substack{p_1\in I_{P_1}\, p_2q_2\in I_{P_2}\\ r_1\in I_{R_1}\, r_2s_2\in I_{R_2}}} 4c_{p_2}c_{r_2}n_{p_1}c_{s_2}c_{q_2}n_{r_1}S_{p_2r_2}S_{r_2p_1}S_{p_1s_2}S_{s_2q_2}v^{p_2r_1}_{q_2r_1}$
(102)	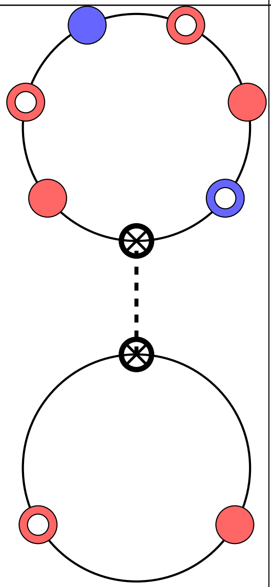	$\sum_{\substack{I_{P_1}\in G(A)\\ I_{R_1}\neq I_{R_2}\neq I_{R_3}\in G(B)}} \sum_{\substack{p_1q_1\in I_{P_1}\\ r_1\in I_{R_1}\, r_2\in I_{R_2}\, r_3\in I_{R_3}}} 4c_{p_1}n_{r_1}c_{q_1}n_{r_2}n_{r_3}S_{p_1r_1}S_{r_1q_1}S_{q_1r_2}v^{p_1r_3}_{r_2r_3}$
(103)	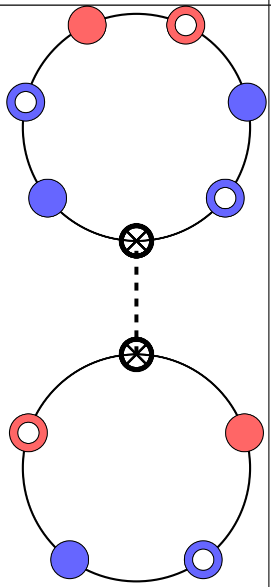	$\sum_{\substack{I_{P_1}\neq I_{P_2}\neq I_{P_3}\in G(A)\\ I_{R_1}\neq I_{R_2}\in G(B)}} \sum_{\substack{p_1\in I_{P_1}\, p_2\in I_{P_2}\, p_3\in I_{P_3}\\ r_1\in I_{R_1}\, r_2s_2\in I_{R_2}}} -4n_{p_1}n_{r_1}n_{p_2}c_{r_2}n_{p_3}c_{s_2}S_{p_1r_1}S_{r_1p_2}S_{r_2p_3}S_{p_3s_2}v^{p_1r_2}_{p_2s_2}$

(104)	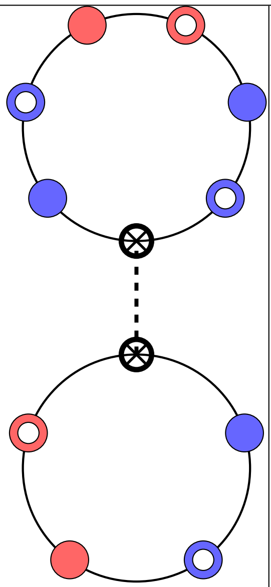	$\sum_{\substack{I_{P_1}\neq I_{P_2}\neq I_{P_3}\in G(A)\\ I_{R_1}\neq I_{R_2}\in G(B)}} \sum_{\substack{p_1\in I_{P_1}\, p_2\in I_{P_2}\, p_3\in I_{P_3}\\ r_1\in I_{R_1}\, r_2\in I_{R_2}}} -4n_{p_1}n_{r_1}n_{p_2}n_{r_2}n_{p_3}S_{p_1r_1}S_{r_1p_2}S_{r_2p_3}v^{p_1r_2}_{p_2p_3}$
(105)	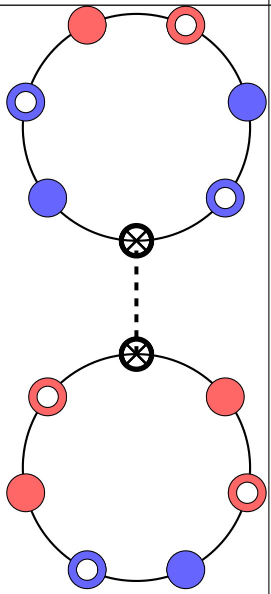	$\sum_{\substack{I_{P_1}\neq I_{P_2}\neq I_{P_3}\in G(A)\\ I_{R_1}\neq I_{R_2}\neq I_{R_3}\in G(B)}} \sum_{\substack{p_1\in I_{P_1}\, p_2\in I_{P_2}\, p_3\in I_{P_3}\\ r_1\in I_{R_1}\, r_2\in I_{R_2}\, r_3\in I_{R_3}}} 4n_{p_1}n_{r_1}n_{p_2}n_{r_2}n_{p_3}n_{r_3}S_{p_1r_1}S_{r_1p_2}S_{r_2p_3}S_{p_3r_3}v^{p_1r_2}_{p_2r_3}$
(106)	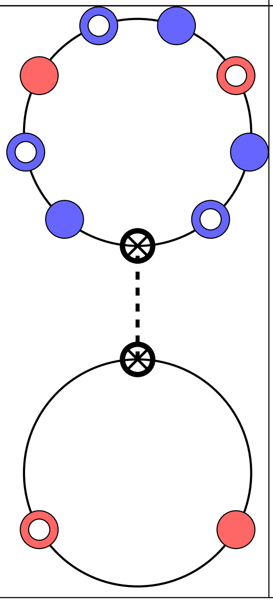	$\sum_{\substack{I_{P_1}\neq I_{P_2}\neq I_{P_3}\in G(A)\\ I_{R_1}\neq I_{R_2}\in G(B)}} \sum_{\substack{p_1\in I_{P_1}\, p_2\in I_{P_2}\, p_3\in I_{P_3}\\ r_1\in I_{R_1}\, r_2 s_2\in I_{R_2}}} -4n_{p_1}c_{r_2}n_{p_2}c_{s_2}n_{p_3}n_{r_1}S_{p_1r_2}S_{r_2p_2}S_{p_2s_2}S_{s_2p_3}v^{p_1r_1}_{p_3r_1}$

(107)	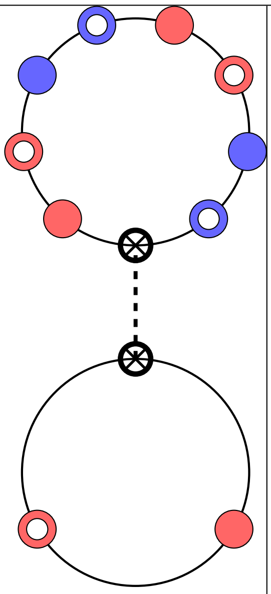	$\sum_{\substack{I_{P_1}\neq I_{P_2}\in G(A)\\ I_{R_1}\neq I_{R_2}\neq I_{R_3}\in G(B)}} \sum_{\substack{p_1\in I_{P_1}\, p_2\in I_{P_2}\\ r_1\in I_{R_1}\, r_2\in I_{R_2}\, r_3\in I_{R_3}}} -4 n_{p_1} n_{r_1} n_{p_2} n_{r_2} n_{r_3} S_{p_1 r_1} S_{r_1 p_2} S_{p_2 r_2} v^{p_1 r_3}_{r_2 r_3}$
(108)	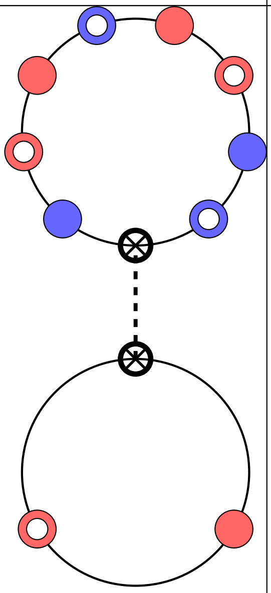	$\sum_{\substack{I_{P_1}\neq I_{P_2}\in G(A)\\ I_{R_1}\neq I_{R_2}\neq I_{R_3}\in G(B)}} \sum_{\substack{p_1\in I_{P_1}\, p_2 q_2\in I_{P_2}\\ r_1\in I_{R_1}\, r_2\in I_{R_2}\, r_3\in I_{R_3}}} -4 n_{p_1} n_{r_1} c_{p_2} n_{r_2} c_{q_2} n_{r_3} S_{p_1 r_1} S_{r_1 p_2} S_{p_2 r_2} S_{r_2 q_2} v^{p_1 r_3}_{q_2 r_3}$
(109)	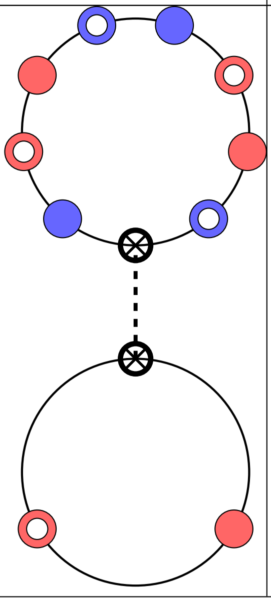	$\sum_{\substack{I_{P_1}\neq I_{P_2}\in G(A)\\ I_{R_1}\neq I_{R_2}\neq I_{R_3}\in G(B)}} \sum_{\substack{p_1\in I_{P_1}\, p_2 q_2\in I_{P_2}\\ r_1\in I_{R_1}\, r_2\in I_{R_2}\, r_3\in I_{R_3}}} -4 c_{p_2} n_{r_1} n_{p_1} n_{r_2} c_{q_2} n_{r_3} S_{p_2 r_1} S_{r_1 p_1} S_{p_1 r_2} S_{r_2 q_2} v^{p_2 r_3}_{q_2 r_3}$

(110)	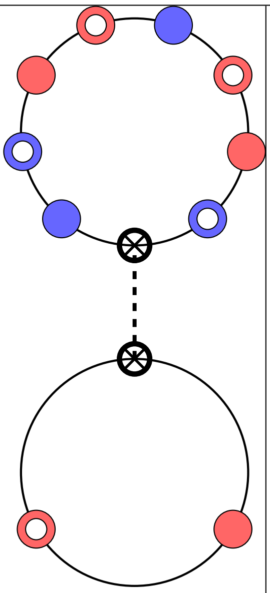	$\sum_{\substack{I_{P_1}\neq I_{P_2}\in G(A)\\ I_{R_1}\neq I_{R_2}\neq I_{R_3}\in G(B)}} \sum_{\substack{p_1\in I_{P_1}\, p_2 q_2\in I_{P_2}\\ r_1\in I_{R_1}\, r_2\in I_{R_2}\, r_3\in I_{R_3}}} -4 c_{p_2} n_{r_1} c_{q_2} n_{r_2} n_{p_1} n_{r_3} S_{p_2 r_1} S_{r_1 q_2} S_{q_2 r_2} S_{r_2 p_1} v^{p_2 r_3}_{p_1 r_3}$
(111)	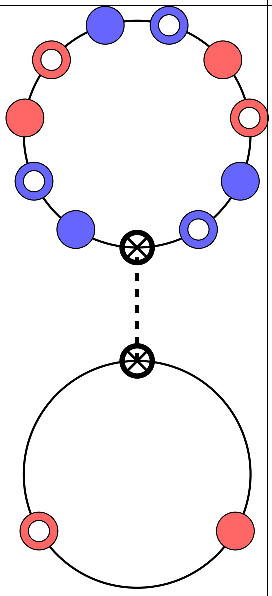	$\sum_{\substack{I_{P_1}\neq I_{P_2}\neq I_{P_3}\in G(A)\\ I_{R_1}\neq I_{R_2}\neq I_{R_3}\in G(B)}} \sum_{\substack{p_1\in I_{P_1}\, p_2\in I_{P_2}\, p_3\in I_{P_3}\\ r_1\in I_{R_1}\, r_2\in I_{R_2}\, r_3\in I_{R_3}}} 4 n_{p_1} n_{r_1} n_{p_2} n_{r_2} n_{p_3} n_{r_3} S_{p_1 r_1} S_{r_1 p_2} S_{p_2 r_2} S_{r_2 p_3} v^{p_1 r_3}_{p_3 r_3}$
(112)	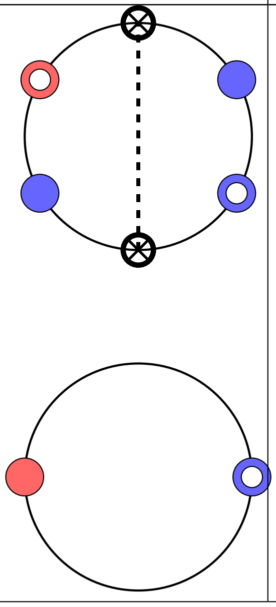	$\frac{1}{2} \sum_{\substack{I_{P_1}\neq I_{P_2}\in G(A)\\ I_{R_1}\in G(B)}} \sum_{\substack{p_1\in I_{P_1}\, p_2 q_2\in I_{P_2}\\ r_1 s_1\in I_{R_1}}} -4 n_{p_1} c_{r_1} c_{p_2} c_{q_2} c_{s_1} S_{r_1 p_2} v^{p_1 p_1}_{p_2 r_1} S_{q_2 s_1} S_{q_2 s_1}$

(113)	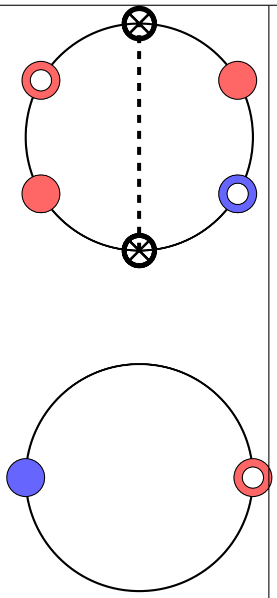	$\frac{1}{2}\sum_{\substack{I_{P_1}\in G(A)\\ I_{R_1}\neq I_{R_2}\in G(B)}}\ \sum_{\substack{p_1q_1\in I_{P_1}\\ r_1\in I_{R_1}\, r_2s_2\in I_{R_2}}} -4c_{p_1}c_{r_2}n_{r_1}c_{s_2}c_{q_1}S_{p_1r_2}v^{p_1r_2}_{r_1r_1}S_{s_2q_1}S_{q_1s_2}$
(114)	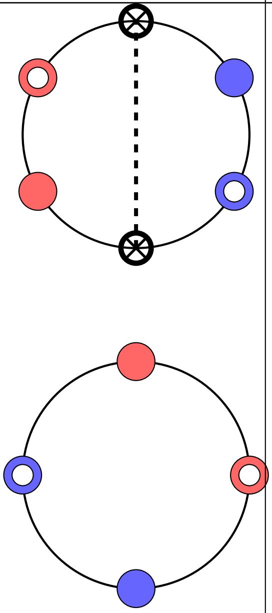	$\sum_{\substack{I_{P_1}\neq I_{P_2}\in G(A)\\ I_{R_1}\neq I_{R_2}\in G(B)}}\ \sum_{\substack{p_1\in I_{P_1}\, p_2\in I_{P_2}\\ r_1\in I_{R_1}\, r_2\in I_{R_2}}} 4n_{p_1}n_{r_1}n_{r_2}n_{p_2}v^{p_1p_1}_{r_1r_1}S_{r_2p_2}S_{p_2r_2}$
(115)	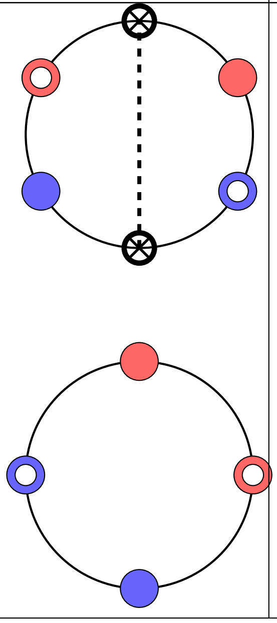	$\sum_{\substack{I_{P_1}\neq I_{P_2}\in G(A)\\ I_{R_1}\neq I_{R_2}\in G(B)}}\ \sum_{\substack{p_1\in I_{P_1}\, p_2q_2\in I_{P_2}\\ r_1\in I_{R_1}\, r_2s_2\in I_{R_2}}} 4c_{p_2}c_{r_2}c_{s_2}c_{q_2}n_{r_1}n_{p_1}S_{p_2r_2}S_{s_2q_2}v^{p_2r_2}_{q_2s_2}S_{r_1p_1}S_{p_1r_1}$

(116)	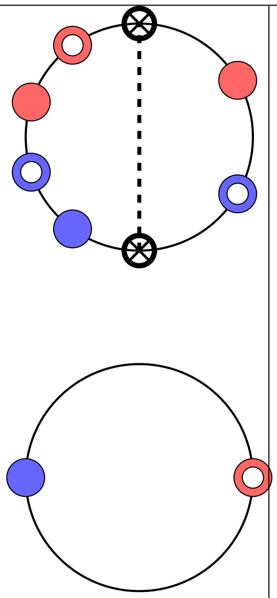	$\frac{1}{2}\sum_{\substack{I_{P_1}\neq I_{P_2}\in G(A)\\ I_{R_1}\neq I_{R_2}\in G(B)}}\ \sum_{\substack{p_1\in I_{P_1}\, p_2q_2\in I_{P_2}\\ r_1\in I_{R_1}\, r_2s_2\in I_{R_2}}} 4c_{p_2}c_{r_2}n_{r_1}n_{p_1}c_{s_2}c_{q_2}S_{p_2r_2}S_{r_1p_1}v^{p_2r_2}_{p_1r_1}S_{s_2q_2}S_{q_2s_2}$
(117)	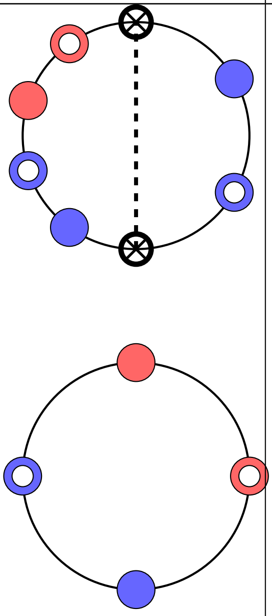	$\sum_{\substack{I_{P_1}\neq I_{P_2}\neq I_{P_3}\in G(A)\\ I_{R_1}\neq I_{R_2}\in G(B)}}\ \sum_{\substack{p_1\in I_{P_1}\, p_2\in I_{P_2}\, p_3\in I_{P_3}\\ r_1\in I_{R_1}\, r_2\in I_{R_2}}} -4n_{p_1}n_{r_1}n_{p_2}n_{r_2}n_{p_3}S_{r_1p_2}v^{p_1p_1}_{p_2r_1}S_{r_2p_3}S_{p_3r_2}$
(118)	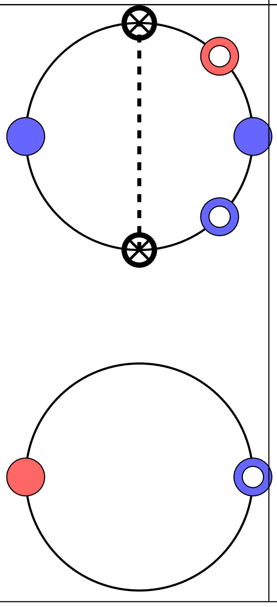	$\frac{1}{2}\sum_{\substack{I_{P_1}\neq I_{P_2}\in G(A)\\ I_{R_1}\in G(B)}}\ \sum_{\substack{p_1\in I_{P_1}\, p_2q_2\in I_{P_2}\\ r_1s_1\in I_{R_1}}} -4n_{p_1}c_{r_1}c_{p_2}c_{q_2}c_{s_1}S_{p_1r_1}v^{p_1r_1}_{p_2p_2}S_{q_2s_1}S_{q_2s_1}$

(119)	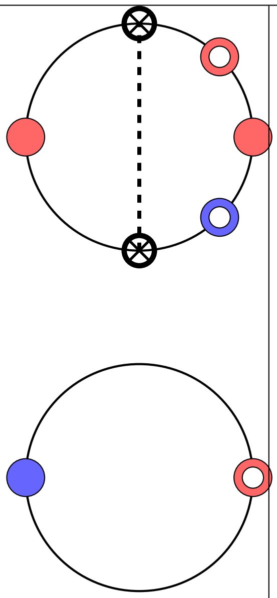	$\frac{1}{2} \sum_{\substack{I_{P_1} \in G(A) \\ I_{R_1} \neq I_{R_2} \in G(B)}} \sum_{\substack{p_1 q_1 \in I_{P_1} \\ r_1 \in I_{R_1}\, r_2 s_2 \in I_{R_2}}} -4 c_{p_1} n_{r_1} c_{r_2} c_{s_2} c_{q_1} S_{p_1 r_1} v^{p_1 r_1}_{r_2 r_2} S_{s_2 q_1} S_{q_1 s_2}$
(120)	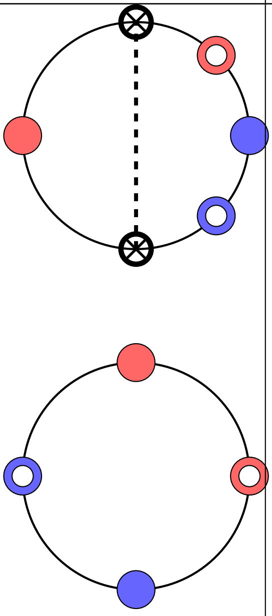	$\sum_{\substack{I_{P_1} \neq I_{P_2} \in G(A) \\ I_{R_1} \neq I_{R_2} \in G(B)}} \sum_{\substack{p_1 \in I_{P_1}\, p_2 \in I_{P_2} \\ r_1 \in I_{R_1}\, r_2 s_2 \in I_{R_2}}} 4 n_{p_1} c_{r_2} c_{s_2} n_{r_1} n_{p_2} S_{p_1 r_2} v^{p_1 r_2}_{s_2 s_2} S_{r_1 p_2} S_{p_2 r_1}$
(121)	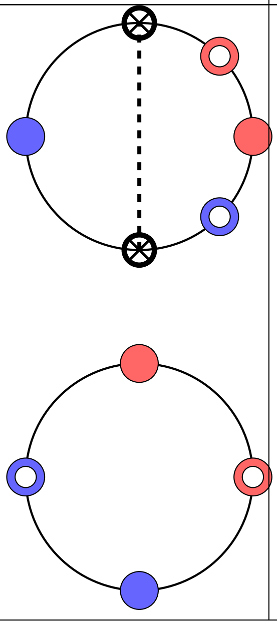	$\sum_{\substack{I_{P_1} \neq I_{P_2} \in G(A) \\ I_{R_1} \neq I_{R_2} \in G(B)}} \sum_{\substack{p_1 \in I_{P_1}\, p_2 q_2 \in I_{P_2} \\ r_1 \in I_{R_1}\, r_2 \in I_{R_2}}} 4 c_{p_2} n_{r_1} c_{q_2} n_{r_2} n_{p_1} S_{p_2 r_1} v^{p_2 r_1}_{q_2 q_2} S_{r_2 p_1} S_{p_1 r_2}$

(122)	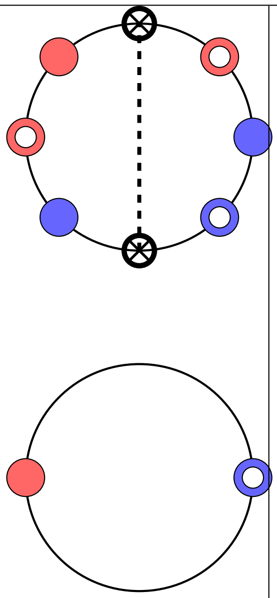	$\frac{1}{2}\sum_{\substack{I_{P_1}\neq I_{P_2}\in G(A)\\ I_{R_1}\neq I_{R_2}\in G(B)}}\sum_{\substack{p_1\in I_{P_1}\, p_2 q_2\in I_{P_2}\\ r_1\in I_{R_1}\, r_2 s_2\in I_{R_2}}} 4n_{p_1}c_{r_2}n_{r_1}c_{p_2}c_{q_2}c_{s_2}S_{p_1r_2}S_{r_1p_2}v_{p_2r_1}^{p_1r_2}S_{q_2s_2}S_{q_2s_2}$
(123)	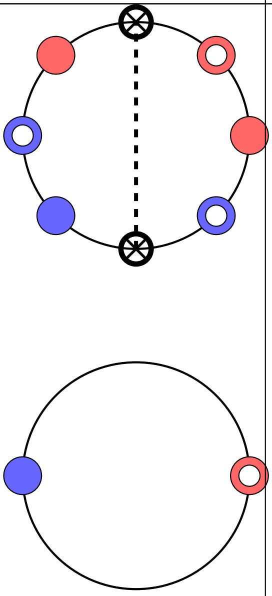	$\frac{1}{2}\sum_{\substack{I_{P_1}\neq I_{P_2}\in G(A)\\ I_{R_1}\neq I_{R_2}\in G(B)}}\sum_{\substack{p_1\in I_{P_1}\, p_2 q_2\in I_{P_2}\\ r_1\in I_{R_1}\, r_2 s_2\in I_{R_2}}} 4c_{p_2}n_{r_1}c_{r_2}n_{p_1}c_{s_2}c_{q_2}S_{p_2r_1}S_{r_2p_1}v_{p_1r_2}^{p_2r_1}S_{s_2q_2}S_{q_2s_2}$
(124)	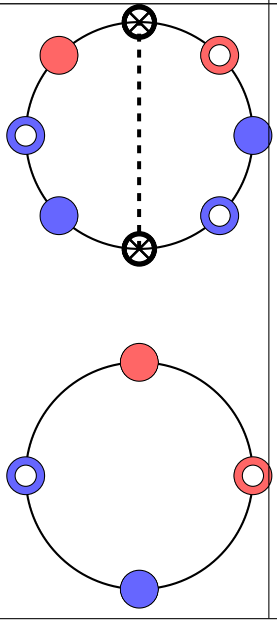	$\sum_{\substack{I_{P_1}\neq I_{P_2}\neq I_{P_3}\in G(A)\\ I_{R_1}\neq I_{R_2}\in G(B)}}\sum_{\substack{p_1\in I_{P_1}\, p_2\in I_{P_2}\, p_3\in I_{P_3}\\ r_1\in I_{R_1}\, r_2 s_2\in I_{R_2}}} -4n_{p_1}c_{r_2}c_{s_2}n_{p_2}n_{r_1}n_{p_3}S_{p_1r_2}S_{s_2p_2}v_{p_2s_2}^{p_1r_2}S_{r_1p_3}S_{p_3r_1}$

(125)	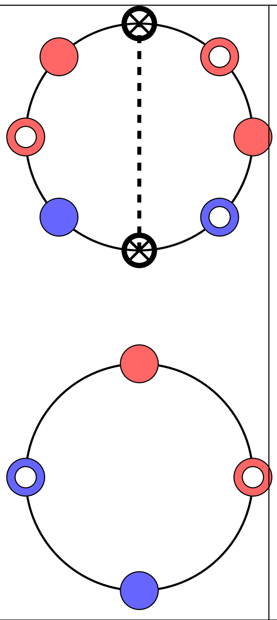	$\sum_{\substack{I_{P_1}\neq I_{P_2}\in G(A)\\ I_{R_1}\neq I_{R_2}\neq I_{R_3}\in G(B)}} \sum_{\substack{p_1\in I_{P_1}\, p_2 q_2\in I_{P_2}\\ r_1\in I_{R_1}\, r_2\in I_{R_2}\, r_3\in I_{R_3}}} -4 c_{p_2} n_{r_1} n_{r_2} c_{q_2} n_{r_3} n_{p_1} S_{p_2 r_1} S_{r_2 q_2} v^{p_2 r_1}_{q_2 r_2} S_{r_3 p_1} S_{p_1 r_3}$
(126)	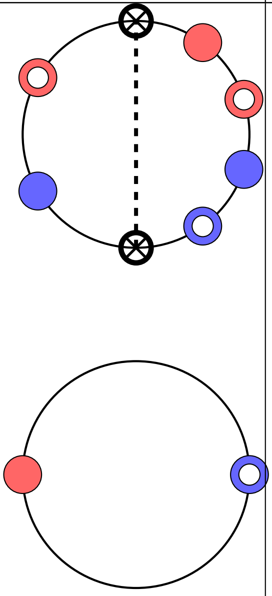	$\frac{1}{2} \sum_{\substack{I_{P_1}\neq I_{P_2}\in G(A)\\ I_{R_1}\neq I_{R_2}\in G(B)}} \sum_{\substack{p_1\in I_{P_1}\, p_2 q_2\in I_{P_2}\\ r_1\in I_{R_1}\, r_2 s_2\in I_{R_2}}} 4 n_{p_1} n_{r_1} c_{r_2} c_{p_2} c_{q_2} c_{s_2} S_{p_1 r_1} S_{r_2 p_2} v^{p_1 r_1}_{p_2 r_2} S_{q_2 s_2} S_{q_2 s_2}$
(127)	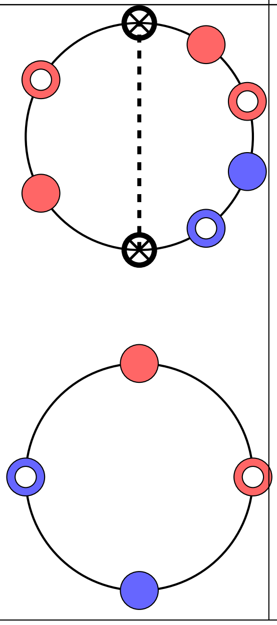	$\sum_{\substack{I_{P_1}\neq I_{P_2}\in G(A)\\ I_{R_1}\neq I_{R_2}\neq I_{R_3}\in G(B)}} \sum_{\substack{p_1\in I_{P_1}\, p_2\in I_{P_2}\\ r_1\in I_{R_1}\, r_2\in I_{R_2}\, r_3\in I_{R_3}}} -4 n_{p_1} n_{r_1} n_{r_2} n_{r_3} n_{p_2} S_{p_1 r_1} v^{p_1 r_1}_{r_2 r_2} S_{r_3 p_2} S_{p_2 r_3}$

(128)	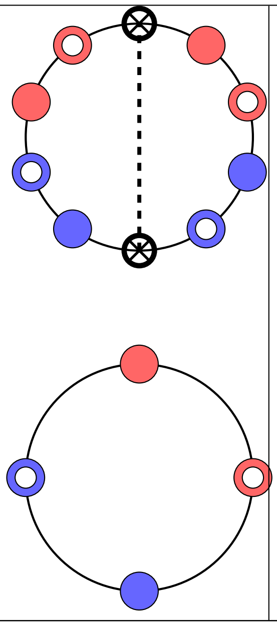	$\sum_{\substack{I_{P_1} \neq I_{P_2} \neq I_{P_3} \in G(A) \\ I_{R_1} \neq I_{R_2} \neq I_{R_3} \in G(B)}} \sum_{\substack{p_1 \in I_{P_1} p_2 \in I_{P_2} p_3 \in I_{P_3} \\ r_1 \in I_{R_1} r_2 \in I_{R_2} r_3 \in I_{R_3}}} 4 n_{p_1} n_{r_1} n_{r_2} n_{p_2} n_{r_3} n_{p_3} S_{p_1 r_1} S_{r_2 p_2} v_{p_2 r_2}^{p_1 r_1} S_{r_3 p_3} S_{p_3 r_3}$

(129)	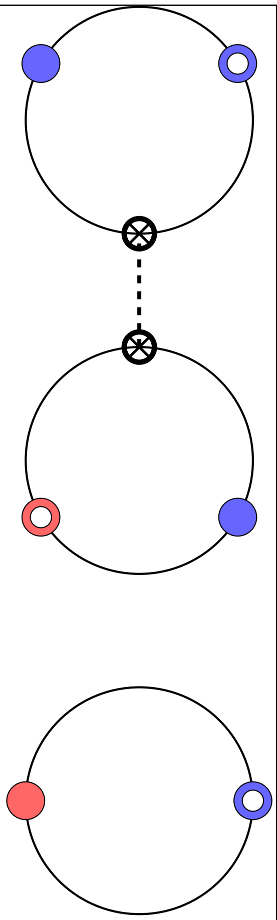	$\frac{1}{2} \sum_{\substack{I_{P_1} \neq I_{P_2} \in G(A) \\ I_{R_1} \in G(B)}} \sum_{\substack{p_1 \in I_{P_1}\, p_2 q_2 \in I_{P_2} \\ r_1 s_1 \in I_{R_1}}} 8 n_{p_1} c_{r_1} c_{p_2} c_{q_2} c_{s_1} S_{r_1 p_2} v_{p_1 p_2}^{p_1 r_1} S_{q_2 s_1} S_{q_2 s_1}$
(130)	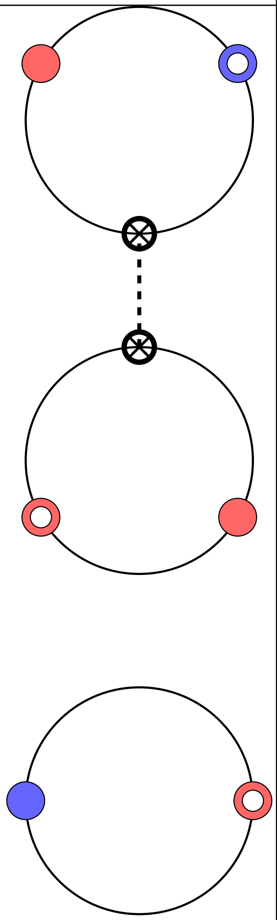	$\frac{1}{2} \sum_{\substack{I_{P_1} \in G(A) \\ I_{R_1} \neq I_{R_2} \in G(B)}} \sum_{\substack{p_1 q_1 \in I_{P_1} \\ r_1 \in I_{R_1}\, r_2 s_2 \in I_{R_2}}} 8 c_{p_1} c_{r_2} n_{r_1} c_{s_2} c_{q_1} S_{p_1 r_2} v_{r_2 r_1}^{p_1 r_1} S_{s_2 q_1} S_{q_1 s_2}$

(131)	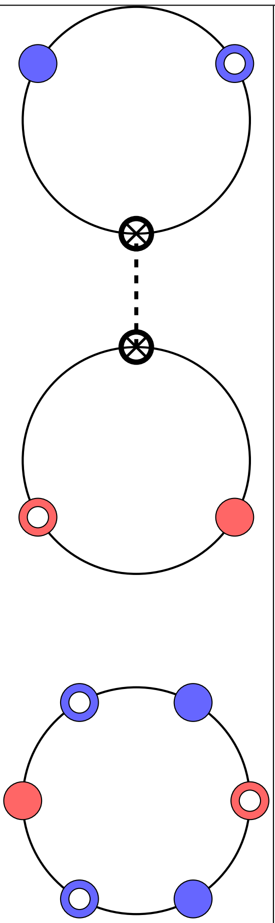	$\frac{1}{2} \sum_{\substack{I_{P_1} \neq I_{P_2} \neq I_{P_3} \in G(A) \\ I_{R_1} \neq I_{R_2} \in G(B)}} \sum_{\substack{p_1 \in I_{P_1} p_2 \in I_{P_2} p_3 \in I_{P_3} \\ r_1 \in I_{R_1} r_2 s_2 \in I_{R_2}}} 8 n_{p_1} n_{r_1} c_{r_2} n_{p_2} c_{s_2} n_{p_3} v^{p_1 r_1}_{p_1 r_1} S_{r_2 p_2} S_{p_2 s_2} S_{s_2 p_3} S_{p_3 r_2}$
(132)	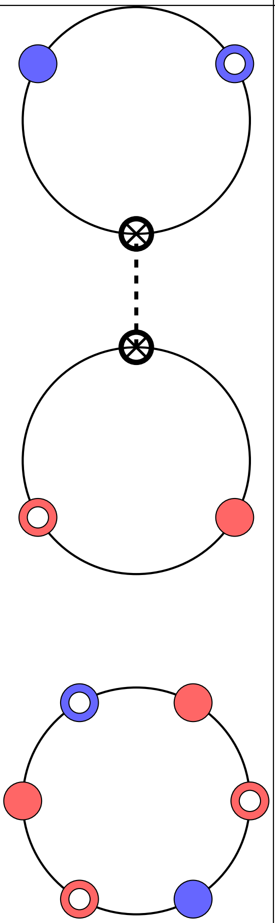	$\frac{1}{2} \sum_{\substack{I_{P_1} \neq I_{P_2} \in G(A) \\ I_{R_1} \neq I_{R_2} \neq I_{R_3} \in G(B)}} \sum_{\substack{p_1 \in I_{P_1} p_2 q_2 \in I_{P_2} \\ r_1 \in I_{R_1} r_2 \in I_{R_2} r_3 \in I_{R_3}}} 8 n_{p_1} n_{r_1} n_{r_2} c_{p_2} n_{r_3} c_{q_2} v^{p_1 r_1}_{p_1 r_1} S_{r_2 p_2} S_{p_2 r_3} S_{r_3 q_2} S_{q_2 r_2}$

(133)	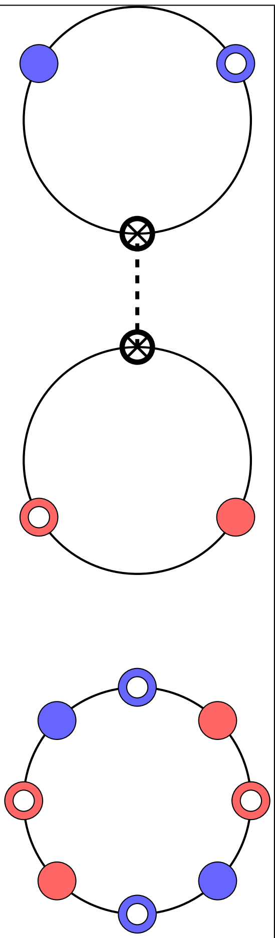	$\frac{1}{2} \sum_{\substack{I_{P_1} \neq I_{P_2} \neq I_{P_3} \in G(A) \\ I_{R_1} \neq I_{R_2} \neq I_{R_3} \in G(B)}} \sum_{\substack{p_1 \in I_{P_1} p_2 \in I_{P_2} p_3 \in I_{P_3} \\ r_1 \in I_{R_1} r_2 \in I_{R_2} r_3 \in I_{R_3}}} -8 n_{p_1} n_{r_1} n_{r_2} n_{p_2} n_{r_3} n_{p_3} v_{p_1 r_1}^{p_1 r_1} S_{r_2 p_2} S_{p_2 r_3} S_{r_3 p_3} S_{p_3 r_2}$
(134)	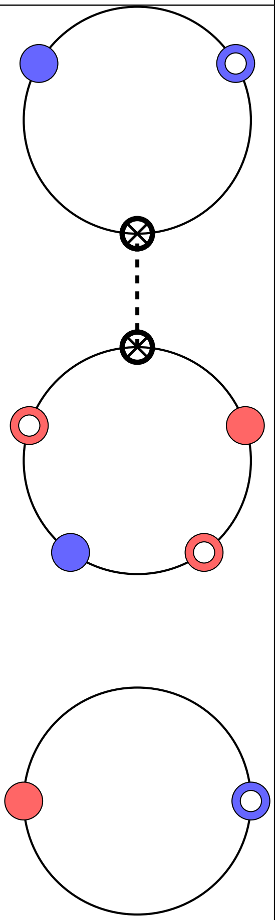	$\frac{1}{2} \sum_{\substack{I_{P_1} \neq I_{P_2} \in G(A) \\ I_{R_1} \neq I_{R_2} \in G(B)}} \sum_{\substack{p_1 \in I_{P_1} p_2 q_2 \in I_{P_2} \\ r_1 \in I_{R_1} r_2 s_2 \in I_{R_2}}} -8 n_{p_1} c_{r_2} c_{p_2} n_{r_1} c_{q_2} c_{s_2} S_{r_2 p_2} S_{p_2 r_1} v_{p_1 r_1}^{p_1 r_2} S_{q_2 s_2} S_{q_2 s_2}$

(135)	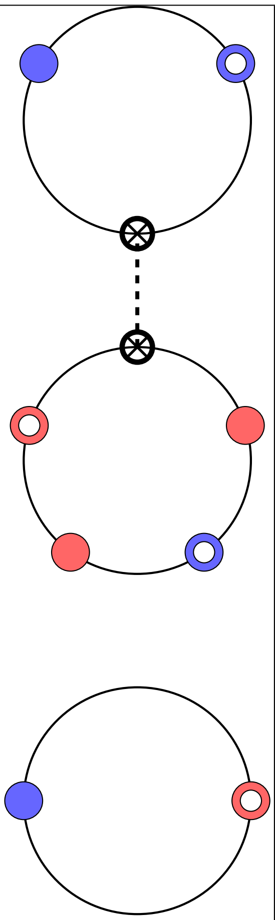	$\frac{1}{2} \sum_{\substack{I_{P_1} \neq I_{P_2} \in G(A) \\ I_{R_1} \neq I_{R_2} \in G(B)}} \sum_{\substack{p_1 \in I_{P_1} \, p_2 q_2 \in I_{P_2} \\ r_1 \in I_{R_1} \, r_2 s_2 \in I_{R_2}}} -8 n_{p_1} n_{r_1} c_{p_2} c_{r_2} c_{s_2} c_{q_2} S_{r_1 p_2} S_{p_2 r_2} v_{p_1 r_2}^{p_1 r_1} S_{s_2 q_2} S_{q_2 s_2}$
(136)	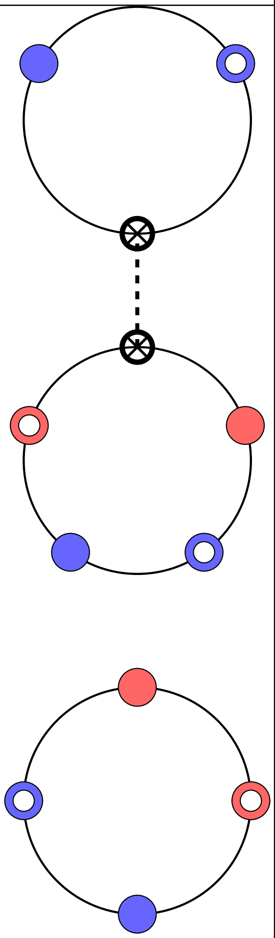	$\sum_{\substack{I_{P_1} \neq I_{P_2} \neq I_{P_3} \in G(A) \\ I_{R_1} \neq I_{R_2} \in G(B)}} \sum_{\substack{p_1 \in I_{P_1} \, p_2 \in I_{P_2} \, p_3 \in I_{P_3} \\ r_1 \in I_{R_1} \, r_2 s_2 \in I_{R_2}}} 8 n_{p_1} c_{r_2} n_{p_2} c_{s_2} n_{r_1} n_{p_3} S_{r_2 p_2} S_{p_2 s_2} v_{p_1 s_2}^{p_1 r_2} S_{r_1 p_3} S_{p_3 r_1}$

(137)	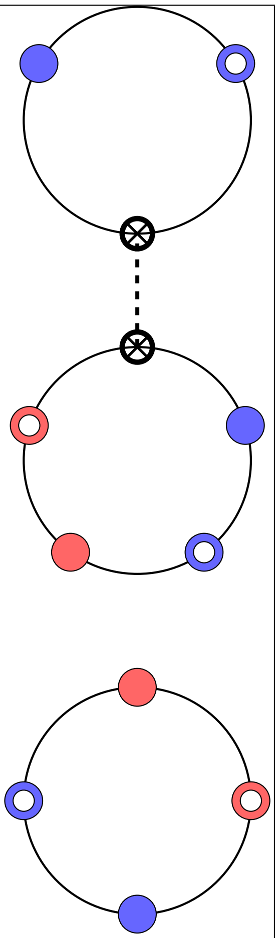	$\sum_{\substack{I_{P_1}\neq I_{P_2}\neq I_{P_3}\in G(A)\\ I_{R_1}\neq I_{R_2}\in G(B)}} \sum_{\substack{p_1\in I_{P_1}\, p_2\in I_{P_2}\, p_3\in I_{P_3}\\ r_1\in I_{R_1}\, r_2\in I_{R_2}}} 8 n_{p_1} n_{r_1} n_{p_2} n_{r_2} n_{p_3} S_{r_1 p_2} v_{p_1 p_2}^{p_1 r_1} S_{r_2 p_3} S_{p_3 r_2}$
(138)	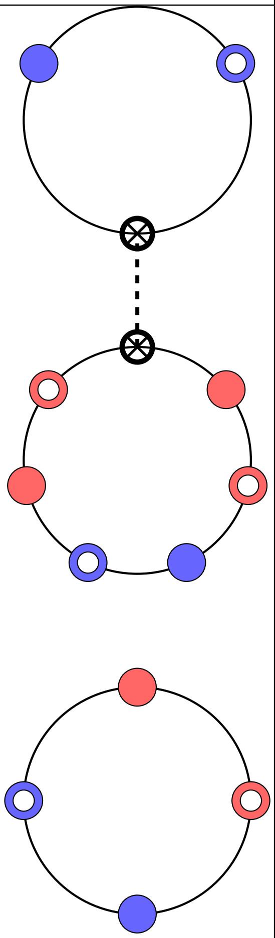	$\sum_{\substack{I_{P_1}\neq I_{P_2}\neq I_{P_3}\in G(A)\\ I_{R_1}\neq I_{R_2}\neq I_{R_3}\in G(B)}} \sum_{\substack{p_1\in I_{P_1}\, p_2\in I_{P_2}\, p_3\in I_{P_3}\\ r_1\in I_{R_1}\, r_2\in I_{R_2}\, r_3\in I_{R_3}}} -8 n_{p_1} n_{r_1} n_{p_2} n_{r_2} n_{r_3} n_{p_3} S_{r_1 p_2} S_{p_2 r_2} v_{p_1 r_2}^{p_1 r_1} S_{r_3 p_3} S_{p_3 r_3}$

(139)	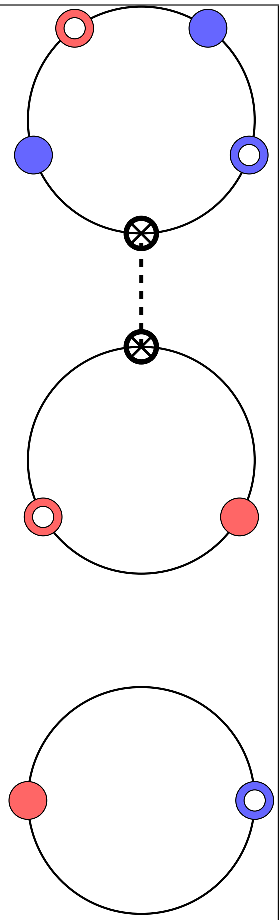	$\frac{1}{2}\sum_{\substack{I_{P_1}\neq I_{P_2}\in G(A)\\ I_{R_1}\neq I_{R_2}\in G(B)}}\sum_{\substack{p_1\in I_{P_1}\, p_2q_2\in I_{P_2}\\ r_1\in I_{R_1}\, r_2s_2\in I_{R_2}}} -8n_{p_1}c_{r_2}c_{p_2}n_{r_1}c_{q_2}c_{s_2}S_{p_1r_2}S_{r_2p_2}v^{p_1r_1}_{p_2r_1}S_{q_2s_2}S_{q_2s_2}$
(140)	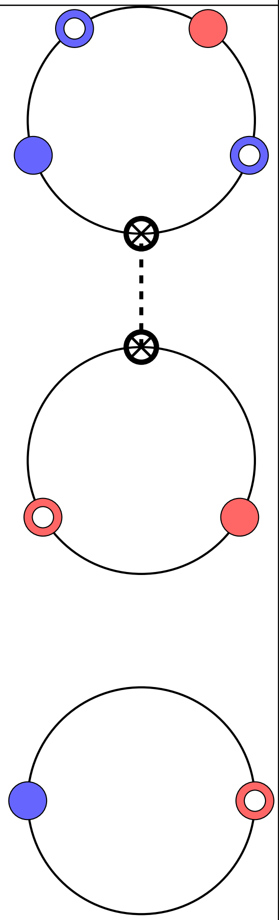	$\frac{1}{2}\sum_{\substack{I_{P_1}\neq I_{P_2}\in G(A)\\ I_{R_1}\neq I_{R_2}\in G(B)}}\sum_{\substack{p_1\in I_{P_1}\, p_2q_2\in I_{P_2}\\ r_1\in I_{R_1}\, r_2s_2\in I_{R_2}}} -8c_{p_2}c_{r_2}n_{p_1}n_{r_1}c_{s_2}c_{q_2}S_{p_2r_2}S_{r_2p_1}v^{p_2r_1}_{p_1r_1}S_{s_2q_2}S_{q_2s_2}$

(141)	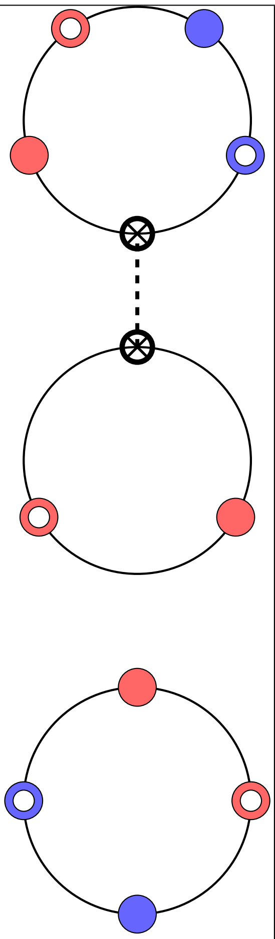	$\sum_{\substack{I_{P_1}\neq I_{P_2}\in G(A)\\ I_{R_1}\neq I_{R_2}\neq I_{R_3}\in G(B)}} \sum_{\substack{p_1\in I_{P_1} p_2\in I_{P_2}\\ r_1\in I_{R_1} r_2\in I_{R_2} r_3\in I_{R_3}}} 8n_{p_1}n_{r_1}n_{r_2}n_{r_3}n_{p_2}S_{p_1r_1}v^{p_1r_2}_{r_1r_2}S_{r_3p_2}S_{p_2r_3}$
(142)	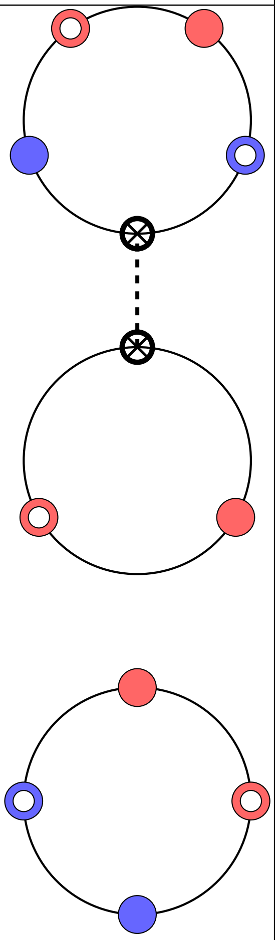	$\sum_{\substack{I_{P_1}\neq I_{P_2}\in G(A)\\ I_{R_1}\neq I_{R_2}\neq I_{R_3}\in G(B)}} \sum_{\substack{p_1\in I_{P_1} p_2q_2\in I_{P_2}\\ r_1\in I_{R_1} r_2\in I_{R_2} r_3\in I_{R_3}}} 8c_{p_2}n_{r_1}c_{q_2}n_{r_2}n_{r_3}n_{p_1}S_{p_2r_1}S_{r_1q_2}v^{p_2r_2}_{q_2r_2}S_{r_3p_1}S_{p_1r_3}$

(143)	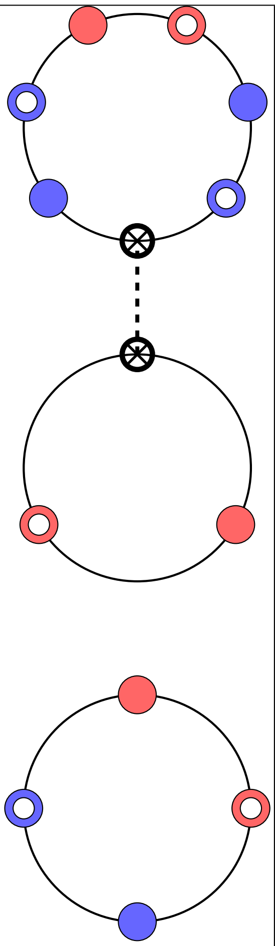	$\sum_{\substack{I_{P_1}\neq I_{P_2}\neq I_{P_3}\in G(A)\\ I_{R_1}\neq I_{R_2}\neq I_{R_3}\in G(B)}} \sum_{\substack{p_1\in I_{P_1} p_2\in I_{P_2} p_3\in I_{P_3}\\ r_1\in I_{R_1} r_2\in I_{R_2} r_3\in I_{R_3}}} -8 n_{p_1} n_{r_1} n_{p_2} n_{r_2} n_{r_3} n_{p_3} S_{p_1 r_1} S_{r_1 p_2} v^{p_1 r_2}_{p_2 r_2} S_{r_3 p_3} S_{p_3 r_3}$

(144)	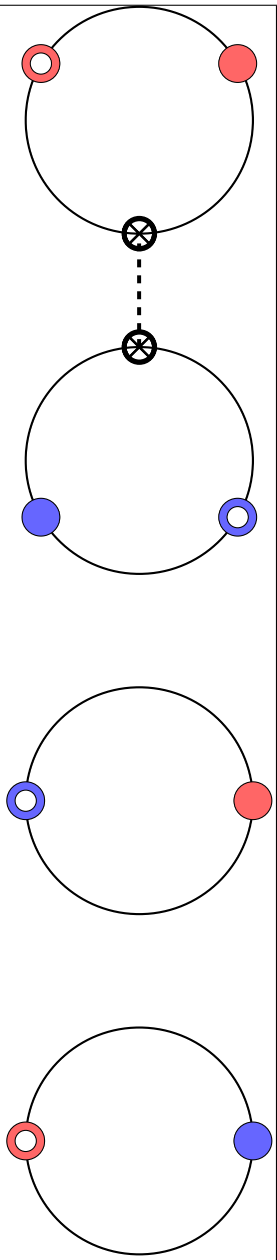	$\frac{1}{2} \cdot \frac{1}{2} \sum_{\substack{I_{P_1} \neq I_{P_2} \in G(A) \\ I_{R_1} \neq I_{R_2} \in G(B)}} \sum_{\substack{p_1 q_1 \in I_{P_1} p_2 \in I_{P_2} \\ r_1 s_1 \in I_{R_1} r_2 \in I_{R_2}}} 16 c_{r_1} c_{p_1} c_{q_1} c_{s_1} S_{r_1 p_1} S_{q_1 s_1} S_{p_1 r_1} S_{q_1 s_1} n_{p_2} n_{r_2} v^{p_2 r_2}_{p_2 r_2}$

(145)	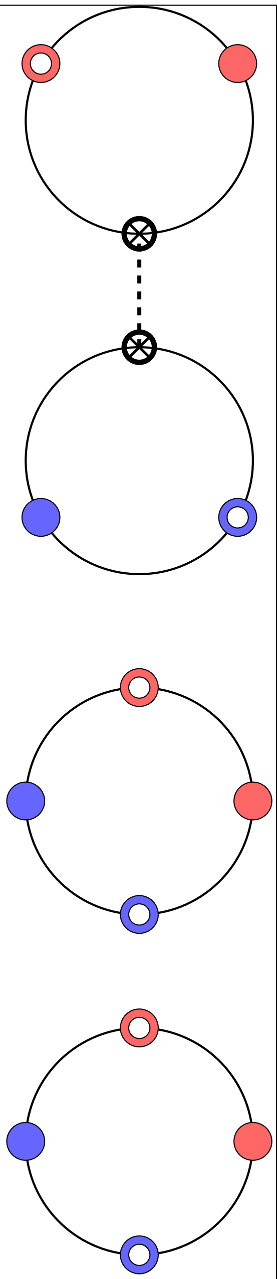	$\frac{1}{2} \sum_{\substack{I_{P_1} \neq I_{P_2} \neq I_{P_3} \in G(A) \\ I_{R_1} \neq I_{R_2} \neq I_{R_3} \in G(B)}} \sum_{\substack{p_1 \in I_{P_1} p_2 \in I_{P_2} p_3 \in I_{P_3} \\ r_1 \in I_{R_1} r_2 \in I_{R_2} r_3 \in I_{R_3}}} 16 n_{r_1} n_{p_1} n_{r_2} n_{p_2} S_{r_1 p_1} S_{r_2 p_2} S_{p_1 r_1} S_{p_2 r_2} n_{p_3} n_{r_3} v_{p_3 r_3}^{p_3 r_3}$

$\langle V_A \mathcal{P}_4 \rangle$

(147)	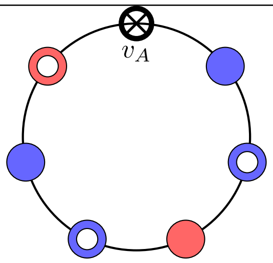	$\sum_{\substack{I_{P_1}\neq I_{P_2}\in G(A)\\ I_{R_1}\in G(B)}} \sum_{\substack{p_1\in I_{P_1}\, p_2\in I_{P_2}\\ r_1 s_1\in I_{R_1}}} 2c_{r_1}n_{p_1}c_{s_1}n_{p_2}S_{r_1p_1}S_{p_1s_1}S_{s_1p_2}(v_A)_{r_1p_2}$
(148)	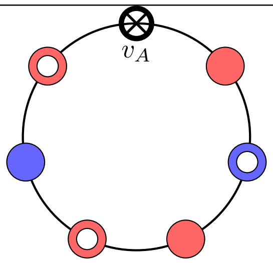	$\sum_{\substack{I_{P_1}\in G(A)\\ I_{R_1}\neq I_{R_2}\in G(B)}} \sum_{\substack{p_1 q_1\in I_{P_1}\\ r_1\in I_{R_1}\, r_2 s_2\in I_{R_2}}} 2c_{r_2}c_{p_1}n_{r_1}c_{q_1}c_{s_2}S_{r_2p_1}S_{p_1r_1}S_{r_1q_1}S_{q_1s_2}(v_A)_{r_2s_2}$
(149)	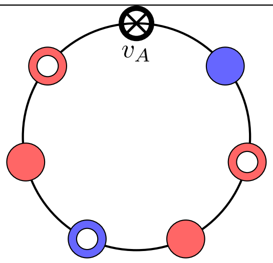	$\sum_{\substack{I_{P_1}\in G(A)\\ I_{R_1}\neq I_{R_2}\in G(B)}} \sum_{\substack{p_1 q_1\in I_{P_1}\\ r_1\in I_{R_1}\, r_2\in I_{R_2}}} 2n_{r_1}c_{p_1}n_{r_2}c_{q_1}S_{r_1p_1}S_{p_1r_2}S_{r_2q_1}(v_A)_{r_1q_1}$
(150)	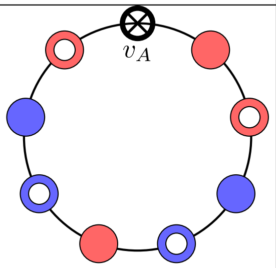	$\sum_{\substack{I_{P_1}\neq I_{P_2}\in G(A)\\ I_{R_1}\neq I_{R_2}\in G(B)}} \sum_{\substack{p_1\in I_{P_1}\, p_2\in I_{P_2}\\ r_1\in I_{R_1}\, r_2 s_2\in I_{R_2}}} -2c_{r_2}n_{p_1}c_{s_2}n_{p_2}n_{r_1}S_{r_2p_1}S_{p_1s_2}S_{s_2p_2}S_{p_2r_1}(v_A)_{r_2r_1}$
(151)	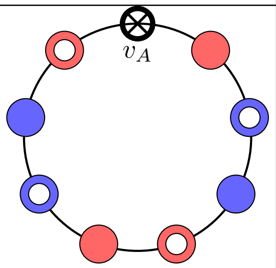	$\sum_{\substack{I_{P_1}\neq I_{P_2}\in G(A)\\ I_{R_1}\neq I_{R_2}\in G(B)}} \sum_{\substack{p_1\in I_{P_1}\, p_2\in I_{P_2}\\ r_1\in I_{R_1}\, r_2 s_2\in I_{R_2}}} -2c_{r_2}n_{p_1}n_{r_1}n_{p_2}c_{s_2}S_{r_2p_1}S_{p_1r_1}S_{r_1p_2}S_{p_2s_2}(v_A)_{r_2s_2}$
(152)	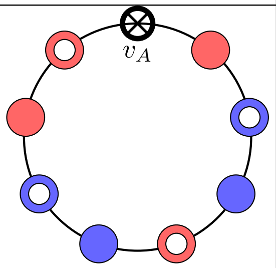	$\sum_{\substack{I_{P_1}\neq I_{P_2}\in G(A)\\ I_{R_1}\neq I_{R_2}\in G(B)}} \sum_{\substack{p_1\in I_{P_1}\, p_2\in I_{P_2}\\ r_1\in I_{R_1}\, r_2 s_2\in I_{R_2}}} -2n_{r_1}n_{p_1}c_{r_2}n_{p_2}c_{s_2}S_{r_1p_1}S_{p_1r_2}S_{r_2p_2}S_{p_2s_2}(v_A)_{r_1s_2}$

(153)	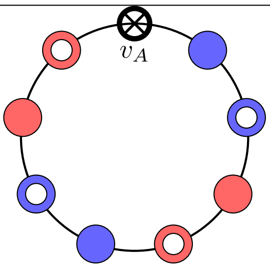	$\sum_{\substack{I_{P_1}\neq I_{P_2}\in G(A)\\ I_{R_1}\neq I_{R_2}\in G(B)}} \sum_{\substack{p_1\in I_{P_1}\, p_2\in I_{P_2}\\ r_1\in I_{R_1}\, r_2\in I_{R_2}}} -2n_{r_1}n_{p_1}n_{r_2}n_{p_2}S_{r_1p_1}S_{p_1r_2}S_{r_2p_2}(v_A)_{r_1p_2}$
(154)	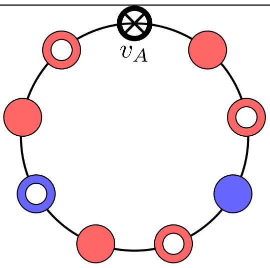	$\sum_{\substack{I_{P_1}\in G(A)\\ I_{R_1}\neq I_{R_2}\neq I_{R_3}\in G(B)}} \sum_{\substack{p_1 q_1\in I_{P_1}\\ r_1\in I_{R_1}\, r_2\in I_{R_2}\, r_3\in I_{R_3}}} -2n_{r_1}c_{p_1}n_{r_2}c_{q_1}n_{r_3}S_{r_1p_1}S_{p_1r_2}S_{r_2q_1}S_{q_1r_3}(v_A)_{r_1r_3}$
(155)	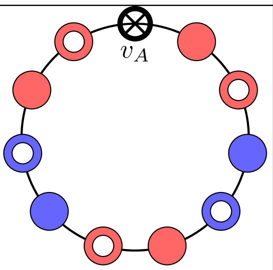	$\sum_{\substack{I_{P_1}\neq I_{P_2}\in G(A)\\ I_{R_1}\neq I_{R_2}\neq I_{R_3}\in G(B)}} \sum_{\substack{p_1\in I_{P_1}\, p_2\in I_{P_2}\\ r_1\in I_{R_1}\, r_2\in I_{R_2}\, r_3\in I_{R_3}}} 2n_{r_1}n_{p_1}n_{r_2}n_{p_2}n_{r_3}S_{r_1p_1}S_{p_1r_2}S_{r_2p_2}S_{p_2r_3}(v_A)_{r_1r_3}$
(156)	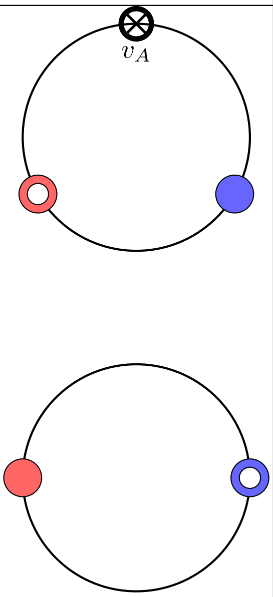	$\frac{1}{2}\sum_{\substack{I_{P_1}\in G(A)\\ I_{R_1}\in G(B)}} \sum_{\substack{p_1 q_1\in I_{P_1}\\ r_1 s_1\in I_{R_1}}} 4c_{r_1}c_{p_1}c_{q_1}c_{s_1}S_{r_1p_1}S_{q_1s_1}(v_A)_{r_1p_1}S_{q_1s_1}$

(157)	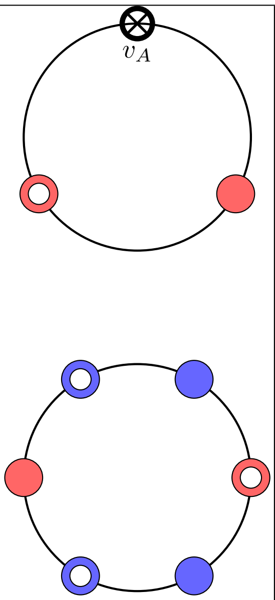	$\frac{1}{2} \sum_{\substack{I_{P_1}\neq I_{P_2}\in G(A)\\ I_{R_1}\neq I_{R_2}\in G(B)}} \sum_{\substack{p_1\in I_{P_1} p_2\in I_{P_2}\\ r_1\in I_{R_1} r_2 s_2\in I_{R_2}}} 4n_{r_1}c_{r_2}n_{p_1}c_{s_2}n_{p_2}S_{r_2p_1}S_{p_1s_2}S_{s_2p_2}(v_A)_{r_1r_1}S_{p_2r_2}$
(158)	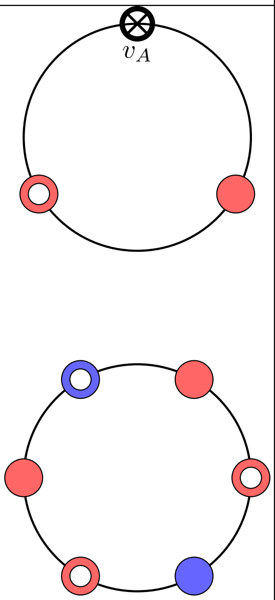	$\frac{1}{2} \sum_{\substack{I_{P_1}\in G(A)\\ I_{R_1}\neq I_{R_2}\neq I_{R_3}\in G(B)}} \sum_{\substack{p_1 q_1\in I_{P_1}\\ r_1\in I_{R_1} r_2\in I_{R_2} r_3\in I_{R_3}}} 4n_{r_1}n_{r_2}c_{p_1}n_{r_3}c_{q_1}S_{r_2p_1}S_{p_1r_3}S_{r_3q_1}(v_A)_{r_1r_1}S_{q_1r_2}$
(159)	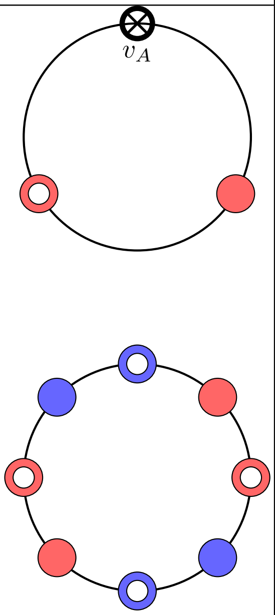	$\frac{1}{2} \sum_{\substack{I_{P_1}\neq I_{P_2}\in G(A)\\ I_{R_1}\neq I_{R_2}\neq I_{R_3}\in G(B)}} \sum_{\substack{p_1\in I_{P_1} p_2\in I_{P_2}\\ r_1\in I_{R_1} r_2\in I_{R_2} r_3\in I_{R_3}}} -4n_{r_1}n_{r_2}n_{p_1}n_{r_3}n_{p_2}S_{r_2p_1}S_{p_1r_3}S_{r_3p_2}(v_A)_{r_1r_1}S_{p_2r_2}$

(160)	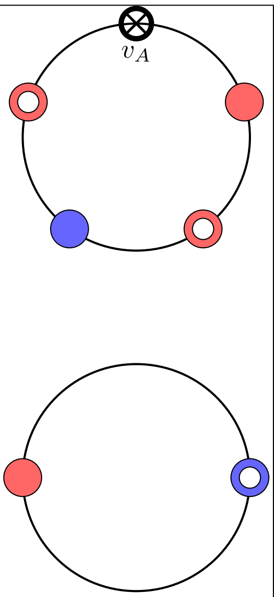	$\frac{1}{2} \sum_{\substack{I_{P_1}\in G(A) \\ I_{R_1}\neq I_{R_2}\in G(B)}} \sum_{\substack{p_1 q_1\in I_{P_1} \\ r_1\in I_{R_1}\, r_2 s_2\in I_{R_2}}} -4 c_{r_2} c_{p_1} n_{r_1} c_{q_1} c_{s_2} S_{r_2 p_1} S_{p_1 r_1} S_{q_1 s_2} (v_A)_{r_2 r_1} S_{q_1 s_2}$
(161)	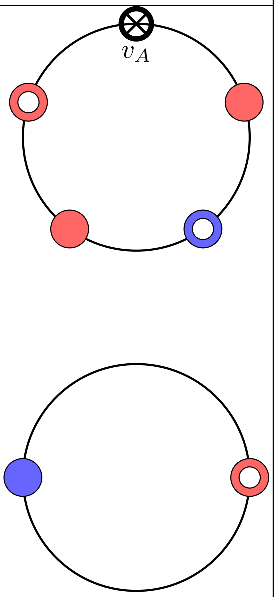	$\frac{1}{2} \sum_{\substack{I_{P_1}\in G(A) \\ I_{R_1}\neq I_{R_2}\in G(B)}} \sum_{\substack{p_1 q_1\in I_{P_1} \\ r_1\in I_{R_1}\, r_2 s_2\in I_{R_2}}} -4 n_{r_1} c_{p_1} c_{r_2} c_{s_2} c_{q_1} S_{r_1 p_1} S_{p_1 r_2} S_{s_2 q_1} (v_A)_{r_1 r_2} S_{q_1 s_2}$
(162)	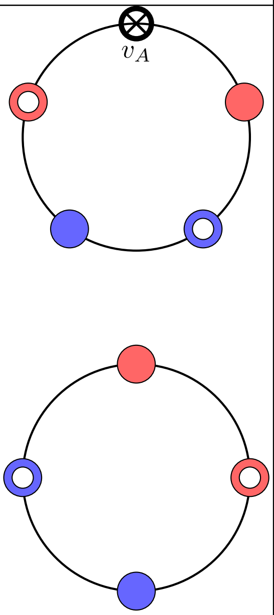	$\sum_{\substack{I_{P_1}\neq I_{P_2}\in G(A) \\ I_{R_1}\neq I_{R_2}\in G(B)}} \sum_{\substack{p_1\in I_{P_1}\, p_2\in I_{P_2} \\ r_1\in I_{R_1}\, r_2 s_2\in I_{R_2}}} 4 c_{r_2} n_{p_1} c_{s_2} n_{r_1} n_{p_2} S_{r_2 p_1} S_{p_1 s_2} S_{r_1 p_2} (v_A)_{r_2 s_2} S_{p_2 r_1}$

(163)	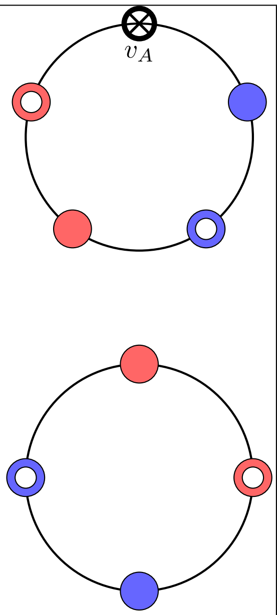	$\sum_{\substack{I_{P_1}\neq I_{P_2}\in G(A)\\ I_{R_1}\neq I_{R_2}\in G(B)}} \sum_{\substack{p_1\in I_{P_1}\, p_2\in I_{P_2}\\ r_1\in I_{R_1}\, r_2\in I_{R_2}}} 4n_{r_1}n_{p_1}n_{r_2}n_{p_2}S_{r_1p_1}S_{r_2p_2}(v_A)_{r_1p_1}S_{p_2r_2}$
(164)	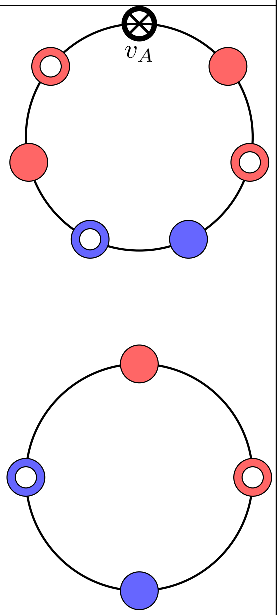	$\sum_{\substack{I_{P_1}\neq I_{P_2}\in G(A)\\ I_{R_1}\neq I_{R_2}\neq I_{R_3}\in G(B)}} \sum_{\substack{p_1\in I_{P_1}\, p_2\in I_{P_2}\\ r_1\in I_{R_1}\, r_2\in I_{R_2}\, r_3\in I_{R_3}}} -4n_{r_1}n_{p_1}n_{r_2}n_{r_3}n_{p_2}S_{r_1p_1}S_{p_1r_2}S_{r_3p_2}(v_A)_{r_1r_2}S_{p_2r_3}$

(165)	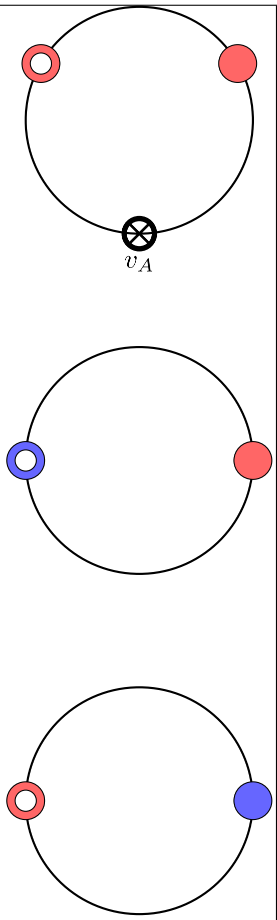	$\frac{1}{2}\cdot\frac{1}{2}\sum_{\substack{I_{P_1}\in G(A)\\ I_{R_1}\neq I_{R_2}\in G(B)}}\sum_{\substack{p_1q_1\in I_{P_1}\\ r_1s_1\in I_{R_1}r_2\in I_{R_2}}} 8c_{r_1}c_{p_1}c_{q_1}c_{s_1}S_{r_1p_1}S_{q_1s_1}S_{p_1r_1}S_{q_1s_1}n_{r_2}(v_A)_{r_2r_2}$
(166)	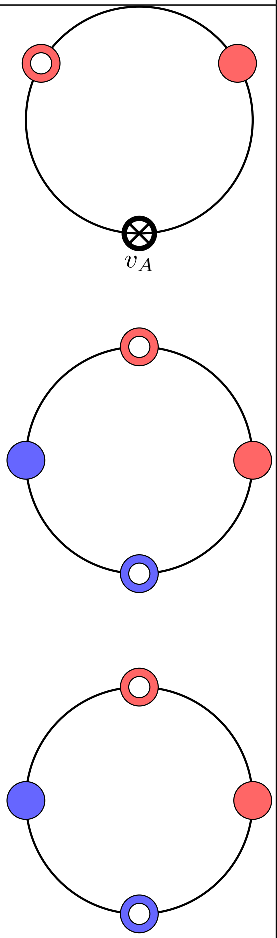	$\frac{1}{2}\sum_{\substack{I_{P_1}\neq I_{P_2}\in G(A)\\ I_{R_1}\neq I_{R_2}\neq I_{R_3}\in G(B)}}\sum_{\substack{p_1\in I_{P_1}p_2\in I_{P_2}\\ r_1\in I_{R_1}r_2\in I_{R_2}r_3\in I_{R_3}}} 8n_{r_1}n_{p_1}n_{r_2}n_{p_2}S_{r_1p_1}S_{r_2p_2}S_{p_1r_1}S_{p_2r_2}n_{r_3}(v_A)_{r_3r_3}$

$\langle V_B \mathcal{P}_4 \rangle$

(167)	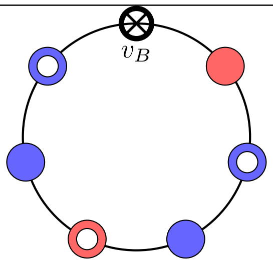	$\sum_{\substack{I_{P_1}\neq I_{P_2}\in G(A)\\ I_{R_1}\in G(B)}} \sum_{\substack{p_1\in I_{P_1}\, p_2\in I_{P_2}\\ r_1 s_1\in I_{R_1}}} 2n_{p_1}c_{r_1}n_{p_2}c_{s_1}S_{p_1r_1}S_{r_1p_2}S_{p_2s_1}(v_B)_{p_1s_1}$
(168)	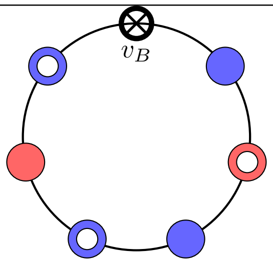	$\sum_{\substack{I_{P_1}\neq I_{P_2}\in G(A)\\ I_{R_1}\in G(B)}} \sum_{\substack{p_1\in I_{P_1}\, p_2 q_2\in I_{P_2}\\ r_1 s_1\in I_{R_1}}} 2c_{p_2}c_{r_1}n_{p_1}c_{s_1}c_{q_2}S_{p_2r_1}S_{r_1p_1}S_{p_1s_1}S_{s_1q_2}(v_B)_{p_2q_2}$
(169)	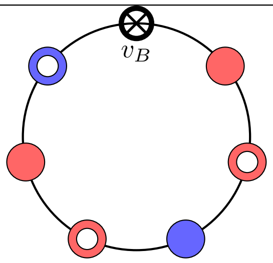	$\sum_{\substack{I_{P_1}\in G(A)\\ I_{R_1}\neq I_{R_2}\in G(B)}} \sum_{\substack{p_1 q_1\in I_{P_1}\\ r_1\in I_{R_1}\, r_2\in I_{R_2}}} 2c_{p_1}n_{r_1}c_{q_1}n_{r_2}S_{p_1r_1}S_{r_1q_1}S_{q_1r_2}(v_B)_{p_1r_2}$
(170)	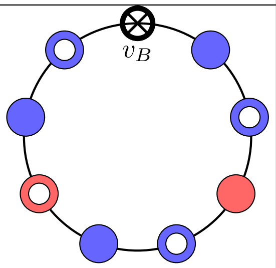	$\sum_{\substack{I_{P_1}\neq I_{P_2}\neq I_{P_3}\in G(A)\\ I_{R_1}\in G(B)}} \sum_{\substack{p_1\in I_{P_1}\, p_2\in I_{P_2}\, p_3\in I_{P_3}\\ r_1 s_1\in I_{R_1}}} -2n_{p_1}c_{r_1}n_{p_2}c_{s_1}n_{p_3}S_{p_1r_1}S_{r_1p_2}S_{p_2s_1}S_{s_1p_3}(v_B)_{p_1p_3}$
(171)	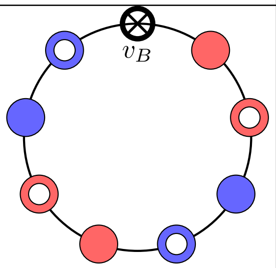	$\sum_{\substack{I_{P_1}\neq I_{P_2}\in G(A)\\ I_{R_1}\neq I_{R_2}\in G(B)}} \sum_{\substack{p_1\in I_{P_1}\, p_2\in I_{P_2}\\ r_1\in I_{R_1}\, r_2\in I_{R_2}}} -2n_{p_1}n_{r_1}n_{p_2}n_{r_2}S_{p_1r_1}S_{r_1p_2}S_{p_2r_2}(v_B)_{p_1r_2}$
(172)	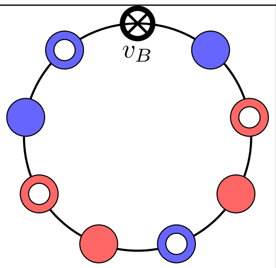	$\sum_{\substack{I_{P_1}\neq I_{P_2}\in G(A)\\ I_{R_1}\neq I_{R_2}\in G(B)}} \sum_{\substack{p_1\in I_{P_1}\, p_2 q_2\in I_{P_2}\\ r_1\in I_{R_1}\, r_2\in I_{R_2}}} -2n_{p_1}n_{r_1}c_{p_2}n_{r_2}c_{q_2}S_{p_1r_1}S_{r_1p_2}S_{p_2r_2}S_{r_2q_2}(v_B)_{p_1q_2}$

(173)	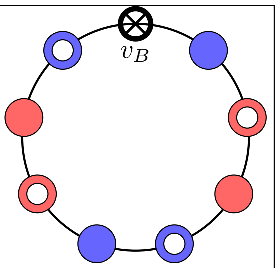	$\sum_{\substack{I_{P_1}\neq I_{P_2}\in G(A)\\ I_{R_1}\neq I_{R_2}\in G(B)}} \sum_{\substack{p_1\in I_{P_1}\, p_2 q_2\in I_{P_2}\\ r_1\in I_{R_1}\, r_2\in I_{R_2}}} -2c_{p_2}n_{r_1}n_{p_1}n_{r_2}c_{q_2}S_{p_2r_1}S_{r_1p_1}S_{p_1r_2}S_{r_2q_2}(v_B)_{p_2q_2}$
(174)	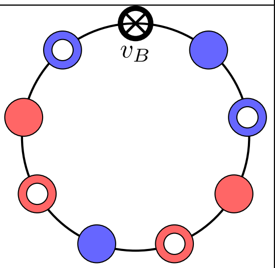	$\sum_{\substack{I_{P_1}\neq I_{P_2}\in G(A)\\ I_{R_1}\neq I_{R_2}\in G(B)}} \sum_{\substack{p_1\in I_{P_1}\, p_2 q_2\in I_{P_2}\\ r_1\in I_{R_1}\, r_2\in I_{R_2}}} -2c_{p_2}n_{r_1}c_{q_2}n_{r_2}n_{p_1}S_{p_2r_1}S_{r_1q_2}S_{q_2r_2}S_{r_2p_1}(v_B)_{p_2p_1}$
(175)	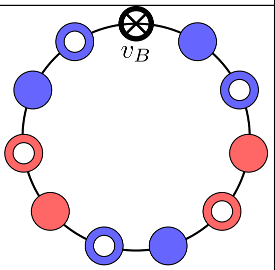	$\sum_{\substack{I_{P_1}\neq I_{P_2}\neq I_{P_3}\in G(A)\\ I_{R_1}\neq I_{R_2}\in G(B)}} \sum_{\substack{p_1\in I_{P_1}\, p_2\in I_{P_2}\, p_3\in I_{P_3}\\ r_1\in I_{R_1}\, r_2\in I_{R_2}}} 2n_{p_1}n_{r_1}n_{p_2}n_{r_2}n_{p_3}S_{p_1r_1}S_{r_1p_2}S_{p_2r_2}S_{r_2p_3}(v_B)_{p_1p_3}$
(176)	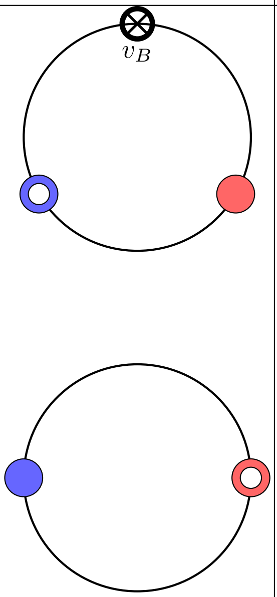	$\frac{1}{2}\sum_{\substack{I_{P_1}\in G(A)\\ I_{R_1}\in G(B)}} \sum_{\substack{p_1 q_1\in I_{P_1}\\ r_1 s_1\in I_{R_1}}} 4c_{p_1}c_{r_1}c_{s_1}c_{q_1}S_{p_1r_1}S_{s_1q_1}(v_B)_{p_1r_1}S_{q_1s_1}$

(177)	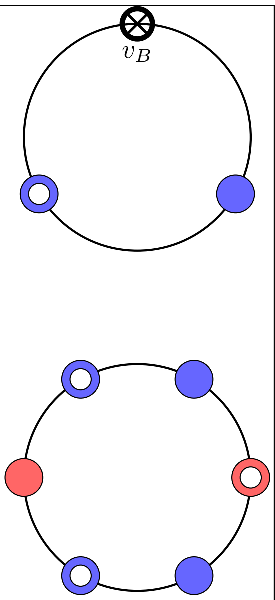	$\frac{1}{2} \sum_{\substack{I_{P_1} \neq I_{P_2} \neq I_{P_3} \in G(A) \\ I_{R_1} \in G(B)}} \sum_{\substack{p_1 \in I_{P_1} p_2 \in I_{P_2} p_3 \in I_{P_3} \\ r_1 s_1 \in I_{R_1}}} 4 n_{p_1} c_{r_1} n_{p_2} c_{s_1} n_{p_3} S_{r_1 p_2} S_{p_2 s_1} S_{s_1 p_3} (v_B)_{p_1 p_1} S_{p_3 r_1}$
(178)	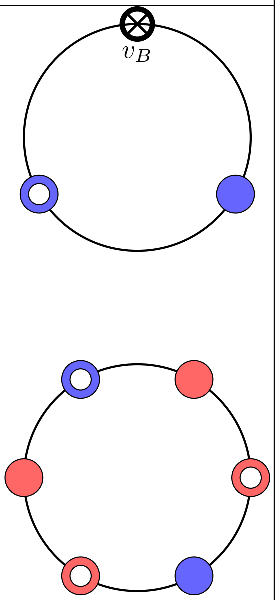	$\frac{1}{2} \sum_{\substack{I_{P_1} \neq I_{P_2} \in G(A) \\ I_{R_1} \neq I_{R_2} \in G(B)}} \sum_{\substack{p_1 \in I_{P_1} p_2 q_2 \in I_{P_2} \\ r_1 \in I_{R_1} r_2 \in I_{R_2}}} 4 n_{p_1} n_{r_1} c_{p_2} n_{r_2} c_{q_2} S_{r_1 p_2} S_{p_2 r_2} S_{r_2 q_2} (v_B)_{p_1 p_1} S_{q_2 r_1}$
(179)	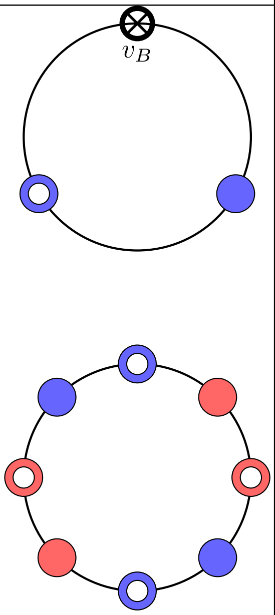	$\frac{1}{2} \sum_{\substack{I_{P_1} \neq I_{P_2} \neq I_{P_3} \in G(A) \\ I_{R_1} \neq I_{R_2} \in G(B)}} \sum_{\substack{p_1 \in I_{P_1} p_2 \in I_{P_2} p_3 \in I_{P_3} \\ r_1 \in I_{R_1} r_2 \in I_{R_2}}} -4 n_{p_1} n_{r_1} n_{p_2} n_{r_2} n_{p_3} S_{r_1 p_2} S_{p_2 r_2} S_{r_2 p_3} (v_B)_{p_1 p_1} S_{p_3 r_1}$

(180)	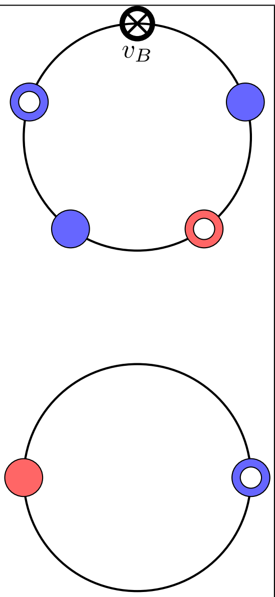	$\frac{1}{2} \sum_{\substack{I_{P_1} \neq I_{P_2} \in G(A) \\ I_{R_1} \in G(B)}} \sum_{\substack{p_1 \in I_{P_1} p_2 q_2 \in I_{P_2} \\ r_1 s_1 \in I_{R_1}}} -4 n_{p_1} c_{r_1} c_{p_2} c_{q_2} c_{s_1} S_{p_1 r_1} S_{r_1 p_2} S_{q_2 s_1} (v_B)_{p_1 p_2} S_{q_2 s_1}$
(181)	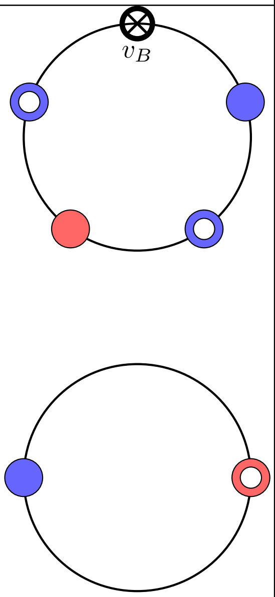	$\frac{1}{2} \sum_{\substack{I_{P_1} \neq I_{P_2} \in G(A) \\ I_{R_1} \in G(B)}} \sum_{\substack{p_1 \in I_{P_1} p_2 q_2 \in I_{P_2} \\ r_1 s_1 \in I_{R_1}}} -4 c_{p_2} c_{r_1} n_{p_1} c_{s_1} c_{q_2} S_{p_2 r_1} S_{r_1 p_1} S_{s_1 q_2} (v_B)_{p_2 p_1} S_{q_2 s_1}$
(182)	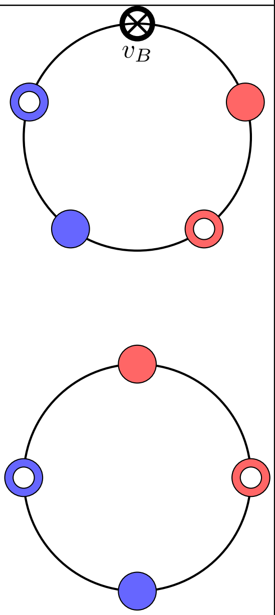	$\sum_{\substack{I_{P_1} \neq I_{P_2} \in G(A) \\ I_{R_1} \neq I_{R_2} \in G(B)}} \sum_{\substack{p_1 \in I_{P_1} p_2 \in I_{P_2} \\ r_1 \in I_{R_1} r_2 \in I_{R_2}}} 4 n_{p_1} n_{r_1} n_{r_2} n_{p_2} S_{p_1 r_1} S_{r_2 p_2} (v_B)_{p_1 r_1} S_{p_2 r_2}$

(183)	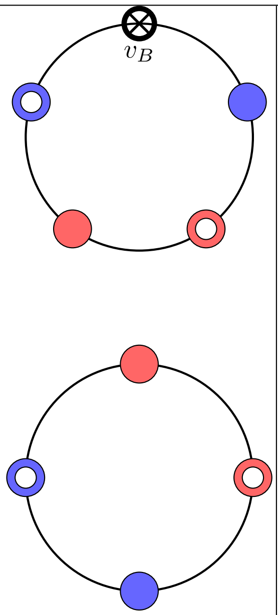	$\sum_{\substack{I_{P_1}\neq I_{P_2}\in G(A)\\ I_{R_1}\neq I_{R_2}\in G(B)}} \sum_{\substack{p_1\in I_{P_1}\, p_2 q_2\in I_{P_2}\\ r_1\in I_{R_1}\, r_2\in I_{R_2}}} 4c_{p_2}n_{r_1}c_{q_2}n_{r_2}n_{p_1}S_{p_2r_1}S_{r_1q_2}S_{r_2p_1}(v_B)_{p_2q_2}S_{p_1r_2}$
(184)	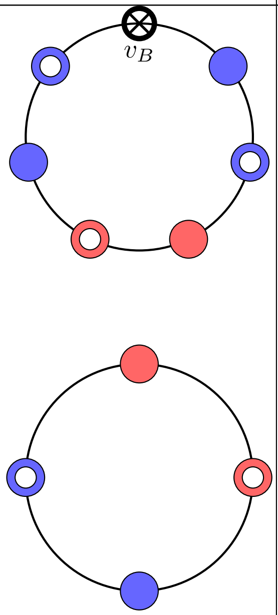	$\sum_{\substack{I_{P_1}\neq I_{P_2}\neq I_{P_3}\in G(A)\\ I_{R_1}\neq I_{R_2}\in G(B)}} \sum_{\substack{p_1\in I_{P_1}\, p_2\in I_{P_2}\, p_3\in I_{P_3}\\ r_1\in I_{R_1}\, r_2\in I_{R_2}}} -4n_{p_1}n_{r_1}n_{p_2}n_{r_2}n_{p_3}S_{p_1r_1}S_{r_1p_2}S_{r_2p_3}(v_B)_{p_1p_2}S_{p_3r_2}$

(185)	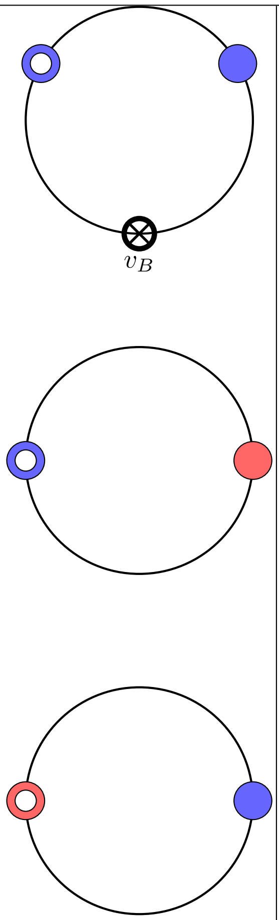	$\frac{1}{2}\cdot\frac{1}{2}\sum_{\substack{I_{P_1}\neq I_{P_2}\in G(A)\\ I_{R_1}\in G(B)}}\sum_{\substack{p_1q_1\in I_{P_1}\,p_2\in I_{P_2}\\ r_1s_1\in I_{R_1}}} 8c_{r_1}c_{p_1}c_{q_1}c_{s_1}S_{r_1p_1}S_{q_1s_1}S_{p_1r_1}S_{q_1s_1}n_{p_2}(v_B)_{p_2p_2}$
(186)	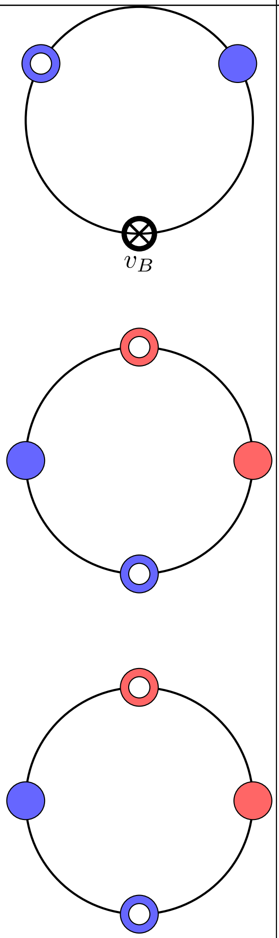	$\frac{1}{2}\sum_{\substack{I_{P_1}\neq I_{P_2}\neq I_{P_3}\in G(A)\\ I_{R_1}\neq I_{R_2}\in G(B)}}\sum_{\substack{p_1\in I_{P_1}\,p_2\in I_{P_2}\,p_3\in I_{P_3}\\ r_1\in I_{R_1}\,r_2\in I_{R_2}}} 8n_{r_1}n_{p_1}n_{r_2}n_{p_2}S_{r_1p_1}S_{r_2p_2}S_{p_1r_1}S_{p_2r_2}n_{p_3}(v_B)_{p_3p_3}$

$\langle \mathcal{P}_4 \rangle$

(187)	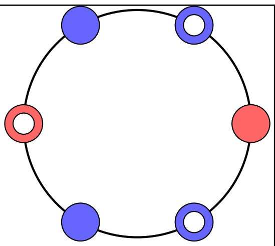	$\frac{1}{2} \sum_{\substack{I_{P_1} \neq I_{P_2} \in G(A) \\ I_{R_1} \in G(B)}} \sum_{\substack{p_1 \in I_{P_1} p_2 \in I_{P_2} \\ r_1 s_1 \in I_{R_1}}} 2 c_{r_1} n_{p_1} c_{s_1} n_{p_2} S_{p_1 r_1} S_{p_1 s_1} S_{p_2 s_1} S_{p_2 r_1}$
(188)	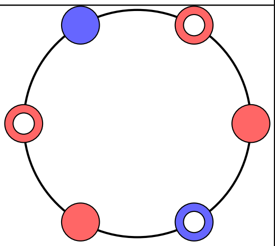	$\frac{1}{2} \sum_{\substack{I_{P_1} \in G(A) \\ I_{R_1} \neq I_{R_2} \in G(B)}} \sum_{\substack{p_1 q_1 \in I_{P_1} \\ r_1 \in I_{R_1} r_2 \in I_{R_2}}} 2 n_{r_1} c_{p_1} n_{r_2} c_{q_1} S_{p_1 r_1} S_{p_1 r_2} S_{q_1 r_2} S_{q_1 r_1}$
(189)	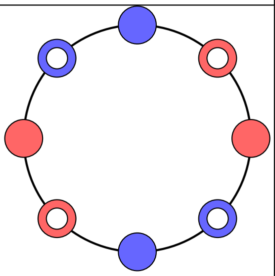	$\frac{1}{2} \sum_{\substack{I_{P_1} \neq I_{P_2} \in G(A) \\ I_{R_1} \neq I_{R_2} \in G(B)}} \sum_{\substack{p_1 \in I_{P_1} p_2 \in I_{P_2} \\ r_1 \in I_{R_1} r_2 \in I_{R_2}}} -2 n_{r_1} n_{p_1} n_{r_2} n_{p_2} S_{p_1 r_1} S_{p_1 r_2} S_{p_2 r_2} S_{p_2 r_1}$
(190)	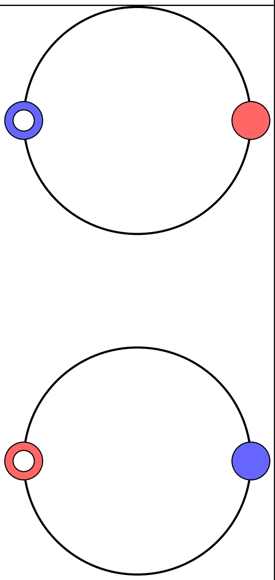	$\frac{1}{2} \cdot \frac{1}{2} \sum_{\substack{I_{P_1} \in G(A) \\ I_{R_1} \in G(B)}} \sum_{\substack{p_1 q_1 \in I_{P_1} \\ r_1 s_1 \in I_{R_1}}} 4 c_{r_1} c_{p_1} c_{q_1} c_{s_1} S_{r_1 p_1} S_{q_1 s_1} S_{p_1 r_1} S_{q_1 s_1}$

(191)	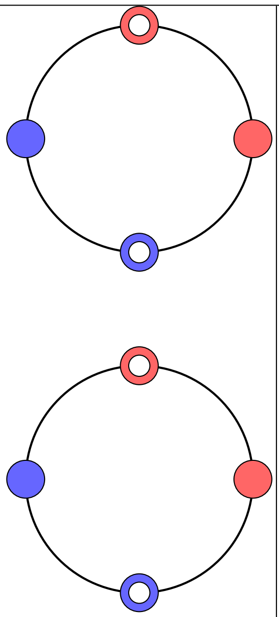	$\frac{1}{2} \sum_{\substack{I_{P_1} \neq I_{P_2} \in G(A) \\ I_{R_1} \neq I_{R_2} \in G(B)}} \sum_{\substack{p_1 \in I_{P_1} p_2 \in I_{P_2} \\ r_1 \in I_{R_1} r_2 \in I_{R_2}}} 4 n_{r_1} n_{p_1} n_{r_2} n_{p_2} S_{r_1 p_1} S_{r_2 p_2} S_{p_1 r_1} S_{p_2 r_2}$
